# Selective inference for k-means clustering

**Published:** 2023-05

**Authors:** Yiqun T. Chen, Daniela M. Witten

**Affiliations:** Data Science Institute and Department of Biomedical Data Science, Stanford University, Stanford, CA 94305, USA; Departments of Statistics and Biostatistics, University of Washington, Seattle, WA 98195-4322, USA

**Keywords:** Post-selection inference, Unsupervised learning, Hypothesis testing, Type I error, RNA-sequencing

## Abstract

We consider the problem of testing for a difference in means between clusters of observations identified via k-means clustering. In this setting, classical hypothesis tests lead to an inflated Type I error rate. In recent work, Gao et al. (2022) considered a related problem in the context of hierarchical clustering. Unfortunately, their solution is highly-tailored to the context of hierarchical clustering, and thus cannot be applied in the setting of k-means clustering. In this paper, we propose a p-value that conditions on all of the intermediate clustering assignments in the k-means algorithm. We show that the p-value controls the selective Type I error for a test of the difference in means between a pair of clusters obtained using k-means clustering in finite samples, and can be efficiently computed. We apply our proposal on hand-written digits data and on single-cell RNA-sequencing data.

## 1. Introduction

Testing for a difference in means between two groups is one of the most fundamental tasks in statistics, with numerous applications. If the groups under investigation are *pre-specified*, i.e., not a function of the observed data, then classical hypothesis tests will control the Type I error rate. However, it is increasingly common to want to test for a difference in means between groups that are *defined through the observed data*, e.g., via the output of a clustering algorithm. For instance, in single-cell RNA-sequencing analysis, researchers often first cluster the cells, and then test for a difference in the expected gene expression levels between the clusters to quantify up- or down-regulation of genes, annotate known cell types, and identify new cell types (Grün et al., 2015; Aizarani et al., 2019; Lähnemann et al., 2020; Zhang et al., 2019; Doughty and Kerkhoven, 2020). In fact, the inferential challenges resulting from testing data-guided hypotheses have been described as a “grand challenge” in the field of genomics (Lähnemann et al., 2020), and papers in the field continue to overlook this issue: as an example, seurat (Stuart et al., 2019), the state-of-the-art single-cell RNA sequencing analysis tool, tests for differential gene expression between groups obtained via clustering, with a note that “p-values [from these hypotheses] should be interpreted cautiously, as the genes used for clustering are the same genes tested for differential expression.” Testing data-guided hypothesis also arises in the field of neuroscience (Kriegeskorte et al., 2009; Button, 2019), social psychology (Hung and Fithian, 2020), and physical sciences (Friederich et al., 2020; Pollice et al., 2021). When the null hypothesis is a function of the data, classical tests that do not account for this will fail to control the Type I error.

**Figure 1:**
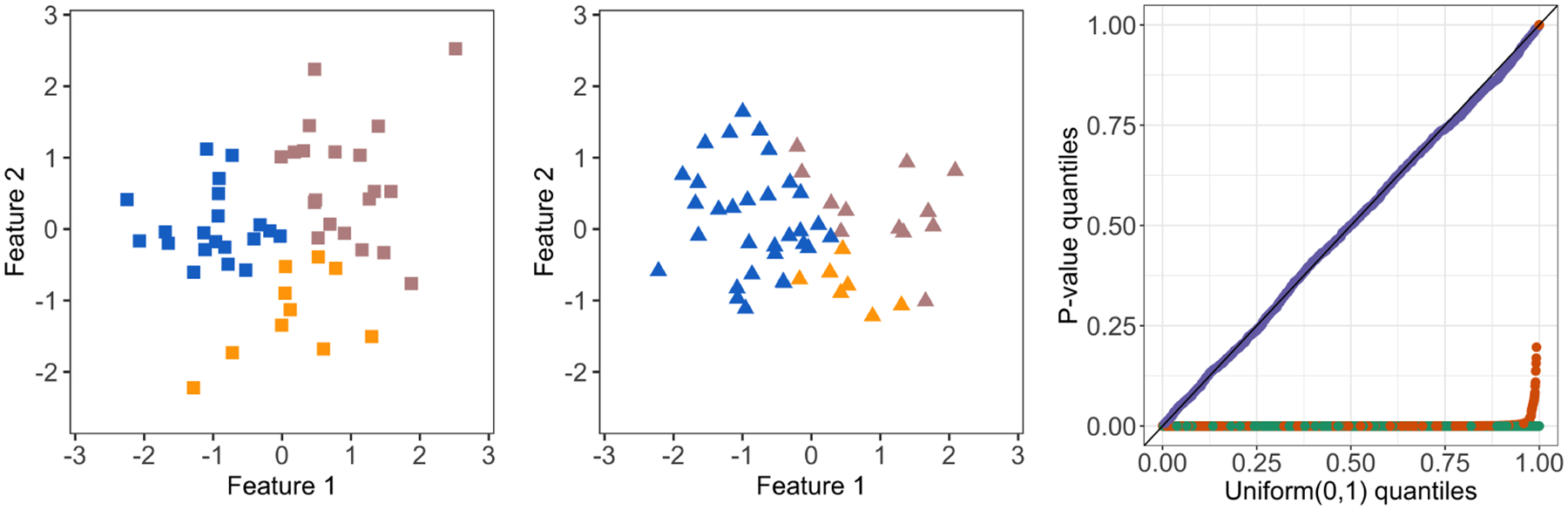
We simulated one dataset from (1) with $\mu =0_{100\times 2}$ and σ=1. We split the data into training (*left*) and test sets (*middle*). *Left*: We apply k-means clustering on the training set to obtain three clusters. *Center*: We apply the training set clusters to the test set using a 3-nearest neighbors classifier. *Right*: Quantile-quantile plot of the naive p-values (4) applied to the training set (green) and the test set (orange), aggregated over 2,000 simulated datasets; as well as our proposed p-values (in (9); displayed in purple) applied to the training set.

In this paper, we develop a test for a difference in means between two clusters estimated from applying k-means clustering (Lloyd, 1982; MacQueen et al., 1967), an extremely popular clustering algorithm with numerous applications (Xu and Wunsch, 2008). In recent work, Gao et al. (2022) tackled a similar problem for hierarchical clustering. While the two papers share similar notation and setup, our solutions and algorithms are tailored to the iterative and centroid-based nature of k-means clustering, leading to fundamentally different solutions and algorithms than those proposed in Gao et al. (2022). We consider the following simple and well-studied model (Gao et al., 2022; Löffler et al., 2021; Lu and Zhou, 2016) for n observations and q features:

(1) $$X\sim ℳ{𝒩}_{n\times q}\left(\mu ,{\text{I}}_{n},\sigma^{2}{\text{I}}_{q}\right),$$

where ℳ𝒩 denotes the matrix normal distribution (Bilodeau and Brenner, 1999), $\mu \in {\mathbb{R}}^{n\times q}$. has unknown rows $\mu_{i}$, and $\sigma^{2}>0$ is known. Given a realization $x\in {\mathbb{R}}^{n\times q}$ of X, we first apply the k-means clustering algorithm to obtain 𝒞(x), a partition of the samples {1, … ,n}. We might then consider testing the null hypothesis that the mean is the same across two *estimated* clusters, i.e.,

(2) $$H_{0}:\sum_{i\in {\hat{𝒞}}_{1}}\mu_{i}/\left|{\hat{𝒞}}_{1}\right|=\sum_{i\in {\hat{𝒞}}_{2}}\mu_{i}/\left|{\hat{𝒞}}_{2}\right|\text{ versus }H_{1}:\sum_{i\in {\hat{𝒞}}_{1}}\mu_{i}/\left|{\hat{𝒞}}_{1}\right|\neq \sum_{i\in {\hat{𝒞}}_{2}}\mu_{i}/\left|{\hat{𝒞}}_{2}\right|,$$

where ${\hat{𝒞}}_{1}$, ${\hat{𝒞}}_{2}\in 𝒞\left(x\right)$ are estimated clusters with cardinality $\left|{\hat{𝒞}}_{1}\right|$ and $\left|{\hat{𝒞}}_{2}\right|$. This is equivalent to testing $H_{0}:\mu^{⊤}\nu =0_{q}$ versus $H_{1}:\mu^{⊤}\nu \neq 0_{q}$, where

(3) $$\nu_{i}=1\left\{i\in {\hat{𝒞}}_{1}\right\}/\left|{\hat{𝒞}}_{1}\right|-1\left\{i\in {\hat{𝒞}}_{2}\right\}/\left|{\hat{𝒞}}_{2}\right|,\;i=1,\;\ldots \;,n,$$

and 1{A} equals 1 if the event A holds, and 0 otherwise. Gao et al. (2022) demonstrates that the p-value given by

(4) $$p_{\text{Naive}}={\text{pr}}_{H_{0}}\left({\left\|X^{⊤}\nu \right\|}_{2}\geq {\left\|x^{⊤}\nu \right\|}_{2}\right),$$

where ${\left\|X^{⊤}\nu \right\|}_{2}\sim \left(\sigma \|\nu {\|}_{2}\right)\chi_{q}$ under $H_{0}$, leads to an extremely anti-conservative test. In particular, we constructed the contrast vector in (3) because ${\hat{𝒞}}_{1}$ and ${\hat{𝒞}}_{2}$ were obtained by clustering. Therefore, we will observe substantial differences between the cluster centroids $\sum_{i\in {\hat{𝒞}}_{1}}x_{i}/\left|{\hat{𝒞}}_{1}\right|$ and $\sum_{i\in {\hat{𝒞}}_{2}}x_{i}/\left|{\hat{𝒞}}_{2}\right|$, even in the absence of true differences in their population means (left panel Figure 1).

Notably, the problem of testing for a difference in means between two groups obtained via clustering cannot be easily overcome by sample splitting, as pointed out in Gao et al. (2022) and Zhang et al. (2019). To see why, we divide the observations into a training and a test set. We apply k-means clustering on only the training set (left panel of Figure 1), and then assign the test set observations to those clusters (to obtain the center panel of Figure 1, we applied a 3-nearest neighbor classifier). Finally, we compute the naive p-values (4) *only* on the test set. Unfortunately, this approach does not work: while we clustered only the training data, we still used the test data to label the test observations, and consequently to construct the contrast vector ν in (3). Therefore, the Wald test based on sample-splitting remains extremely anti-conservative, as shown in the right panel of Figure 1, and does not lead to a valid test of $H_{0}$ in (2). We refer the readers to Gao et al. (2022) for further discussion of this point.

In this paper, we develop a test of $H_{0}$ that controls the selective Type I error. That is, we wish to ensure that the probability of rejecting $H_{0}$ at level α, given that $H_{0}$ holds and we decided to test it, is no greater than α:

(5) $${\text{pr}}_{H_{0}}\left(\text{reject }H_{0}\text{ at level }\alpha |H_{0}\text{ is tested}\right)\leq \alpha ,\;\forall \alpha \in \left(0,1\right).$$

To develop the test, we leverage the selective inference framework, which has been applied extensively in high-dimensional linear modeling (Lee et al., 2016; Tibshirani et al., 2016; Fithian et al., 2014; Rügamer et al., 2022; Schultheiss et al., 2021; Taylor and Tibshirani, 2018; Charkhi and Claeskens, 2018; Yang et al., 2016; Loftus and Taylor, 2015), changepoint detection (Jewell et al., 2022; Hyun et al., 2021, 2018; Chen et al., 2021; Le Duy and Takeuchi, 2021; Duy et al., 2020; Benjamini et al., 2019), and clustering (Zhang et al., 2019; Gao et al., 2022; Watanabe and Suzuki, 2021). The key insight behind selective inference is as follows: naive p-values such as (4) lead to anti-conservative tests because the hypothesis $H_{0}$ is generated by the same data used for testing. Therefore, to obtain a valid test of $H_{0}$, we need to condition on the aspect of the data that led us to test $H_{0}$. In our case, we chose to test the null hypothesis in (2) because ${\hat{𝒞}}_{1}$ and ${\hat{𝒞}}_{2}$ were obtained via k-means clustering. Therefore, we compute a p-value conditional on the event that k-means clustering yields ${\hat{𝒞}}_{1}$ and ${\hat{𝒞}}_{2}$. This results in selective Type I error control (5), as seen in the right panel of Figure 1.

There is a rich literature on estimating and quantifying the uncertainty in the number of clusters (Li and Chen, 2010; Chen and Li, 2009; Chen et al., 2004; McLachlan et al., 2019; Dobriban, 2020), as well as assessing cluster stability and heterogeneity (Suzuki and Shimodaira, 2006; Kerr and Churchill, 2001; Kimes et al., 2017; Chung, 2020; Jin and Wang, 2016; Aw et al., 2021; Chung and Storey, 2015). Others have examined the asymptotic properties of clustering models from a Bayesian perspective (Guha et al., 2019; Nobile, 2004; Cai et al., 2020). In addition, k-means clustering is a special case of the expectation-maximization algorithm, which allows us to tap into an active line of research on the statistical guarantees of the expectation-maximization algorithm (Zhang and Zhang, 2014; Wang et al., 2015; Cai et al., 2019; Yi and Caramanis, 2015; Balakrishnan et al., 2017). However, most prior work focused the setting with one or more “true” clusters. By contrast, we are interested in a correctly-sized test for the null hypothesis (2), even when ${\hat{𝒞}}_{1}$, ${\hat{𝒞}}_{2}$ do not correspond to “true” clusters, and even in the absence of “true” clusters in the data. In addition, existing work often relies on asymptotic approximations and bootstrap resampling. Two recent exceptions include Zhang et al. (2019) and Gao et al. (2022), who took a selective inference approach and computed finite-sample p-values for testing the difference in means between estimated clusters obtained via linear classification rules and hierarchical clustering, respectively. Our work is closest to Gao et al. (2022), and extends their framework to k-means clustering. We provide an exact, finite-sample test of the difference in means between a pair of clusters estimated via k-means clustering under model (1), without the need for sample splitting.

The rest of this paper is organized as follows. In Section 2, we briefly review the work of Gao et al. (2022), and outline our proposed test of a difference in means after k-means clustering. It is worth highlighting that while our proposal is inspired by the work of Gao et al. (2022), our solution is *not* simply a minor modification: computing the conditioning set for the p-value in (9) is the key technical challenge of this paper, and the computational insights in Gao et al. (2022) are only applicable to hierarchical clustering. In Section 3, we provide a computationally-efficient approach to compute the p-values corresponding to our proposed test, for a difference in means after k-means clustering. Section 4 outlines some extensions, and we evaluate our proposal in a simulation study in Section 5. We apply our proposal to three real datasets in Section 6, and discuss future work in Section 7. Proofs and additional results are relegated to the Appendix.

Throughout this paper, we will use the following notational conventions. For a matrix A, $A_{i}$ denotes the ith row and $A_{ij}$ denotes the (i,j)th entry. For a vector $\nu \in {\mathbb{R}}^{n}$, $\|\nu {\|}_{2}$ denotes its $\ell_{2}$ norm, and $\Pi_{\nu }^{⊥}$ is the projection matrix onto the orthogonal complement of ν, i.e., $\Pi_{\nu }^{⊥}=I_{n}-\nu \nu^{⊤}/\|\nu {\|}_{2}^{2}$, where $I_{n}$ is the n-dimensional identity matrix. Moreover, $\text{dir}\left(\nu \right)=\nu /\|\nu {\|}_{2}$ if $\nu \neq 0_{n}$ and $0_{n}$ otherwise, where $0_{n}$ is the n-vector of zeros. We let 〈⋅,⋅〉 and 1{⋅} denote the inner product of two vectors and the indicator function, respectively.

## 2. Selective inference for k-means clustering

### 2.1. A brief review of k-means clustering

In this section, we review the k-means clustering algorithm. Given samples $x_{1},\;\ldots \;,x_{n}\in {\mathbb{R}}^{q}$ and a positive integer K, k-means clustering partitions the n samples into disjoint subsets ${\hat{𝒞}}_{1},\ldots ,{\hat{𝒞}}_{K}$ by solving the following optimization problem:

(6) $$\underset{{𝒞}_{1},\ldots ,{𝒞}_{K}}{\text{minimize}}\left\{\overset{K}{\underset{k=1}{\sum }}\underset{i\in {𝒞}_{k}}{\sum }{\left\|x_{i}-\underset{i\in {𝒞}_{k}}{\sum }x_{i}/\left|{𝒞}_{k}\right|\right\|}_{2}^{2}\right\}\;\text{subject to}\overset{K}{\underset{k=1}{\cup }}{𝒞}_{k}=\left\{1,\;\ldots \;,n\right\},{𝒞}_{k}\cap {𝒞}_{k^{\prime }}=\emptyset ,\forall k\neq k^{\prime }.$$

It is not typically possible to solve for the global optimum in (6) (Aloise et al., 2009). A number of algorithms are available to find a local optimum (Hartigan and Wong, 1979; Zha et al., 2002; Arthur and Vassilvitskii, 2007); one such approach is Lloyd’s algorithm (Lloyd, 1982), given in Algorithm 1. We first sample K out of n observations as initial centroids (step 1 in Algorithm 1). We then assign each observation to the closest centroid (step 2). Next, we iterate between re-computing the centroids and updating the cluster assignments (steps 3a. and 3b.) until the cluster assignments stop changing. The algorithm is guaranteed to converge to a local optimum (Hastie et al., 2001).

In what follows, we will sometimes use $c_{i}^{\left(t\right)}\left(x\right)$ and $m_{k}^{\left(t\right)}\left(x\right)$ rather than $c_{i}^{\left(t\right)}$ and $m_{k}^{\left(t\right)}$ to emphasize the dependence of the cluster labels and centroids on the data x.


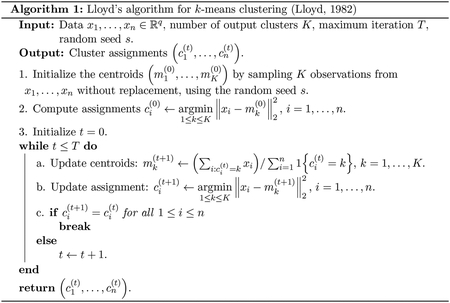


### 2.2. A test of (2) for clusters obtained via k-means clustering

Here, we briefly review the proposal of Gao et al. (2022) for selective inference for hierarchical clustering, and outline a selective test for (2) for k-means clustering.

Gao et al. (2022) proposed a selective inference framework for testing hypotheses based on the output of a clustering algorithm. Let 𝒞(⋅) denote the clustering operator, i.e., a partition of the observations resulting from a clustering algorithm. Since $H_{0}$ in (2) is chosen because $\left\{{\hat{𝒞}}_{1},{\hat{𝒞}}_{2}\in 𝒞\left(x\right)\right\}$, where ${\hat{𝒞}}_{1}$ , ${\hat{𝒞}}_{2}$ are the two estimated clusters under consideration in (2), Gao et al. (2022) proposed to reject $H_{0}$ if

(7) $${\text{pr}}_{H_{0}}\left\{{\left\|X^{⊤}\nu \right\|}_{2}\geq {\left\|x^{⊤}\nu \right\|}_{2}|{\hat{𝒞}}_{1},{\hat{𝒞}}_{2}\in 𝒞\left(X\right),{\text{Π}}_{\nu }^{⊥}X={\text{Π}}_{\nu }^{⊥}x,\text{ dir}\left(X^{⊤}\nu \right)=\text{dir}\left(x^{⊤}\nu \right)\right\}$$

is small. In (7), conditioning on $\{\Pi_{\nu }^{⊥}X=\Pi_{\nu }^{⊥}x,\;\text{dir}\left(X^{⊤}\nu \right)=\text{dir}\left(x^{⊤}\nu \right)\}$ eliminates the nuisance parameters $\Pi_{\nu }^{⊥}\mu$ and $\text{dir}\left(\mu^{⊤}\nu \right)$, where $\Pi_{\nu }^{⊥}={\text{I}}_{n}-\nu \nu^{⊤}/\|\nu {\|}_{2}$ and $\text{dir}\left(\mu^{⊤}\nu \right)=\mu^{⊤}\nu /\mu^{⊤}\nu_{2}$ (see, e.g., Section 3.1 of Fithian et al. (2014)). Gao et al. (2022) showed that the test that rejects $H_{0}$ when (7) is below α controls the selective Type I error at level α, in the sense of (5). Furthermore, under (1), the conditional distribution of ${\left\|X^{⊤}\nu \right\|}_{2}$ in (7) is $\left(\sigma \|\nu {\|}_{2}\right)\chi_{q}$, truncated to a set. When the operator 𝒞(⋅) denotes hierarchical clustering, this set can be analytically characterized and efficiently computed, leading to an efficient algorithm for computing (7).

We now extend these ideas to k-means clustering (6). Since the k-means algorithm partitions all n observations, it is natural to condition on the cluster assignments of *all* observations rather than just on $\left\{{\hat{𝒞}}_{1},{\hat{𝒞}}_{2}\in 𝒞\left(X\right)\right\}$. This leads to the p-value

(8) $${\text{pr}}_{H_{0}}\left[{\left\|X^{⊤}\nu \right\|}_{2}\geq {\left\|x^{⊤}\nu \right\|}_{2}|\overset{n}{\underset{i=1}{\cap }}\left\{c_{i}^{\left(T\right)}\left(X\right)=c_{i}^{\left(T\right)}\left(x\right)\right\},\Pi_{\nu }^{⊥}X=\Pi_{\nu }^{⊥}x,\text{ dir}\left(X^{⊤}\nu \right)=\text{dir}\left(x^{⊤}\nu \right)\right],$$

where $c_{i}^{\left(T\right)}\left(X\right)$ is the cluster assigned to the ith observation at the final iteration of Algorithm 1. However, computing (8) requires characterizing $\cap_{i=1}^{n}\left\{c_{i}^{\left(T\right)}\left(X\right)=c_{i}^{\left(T\right)}\left(x\right)\right\}$, which is not straightforward, and may necessitate enumerating over possibly an exponential number of intermediate cluster assignments $c_{i}^{\left(t\right)}\left(\cdot \right)$ for t=1, … ,T−1. Hence, we also condition on *all of the intermediate clustering assignments* in Algorithm 1:

(9) $$p_{\text{selective}}={\text{pr}}_{H_{0}}\left[{\left\|X^{⊤}\nu \right\|}_{2}\geq {\left\|x^{⊤}\nu \right\|}_{2}|\overset{T}{\underset{t=0}{\cap }}\overset{n}{\underset{i=1}{\cap }}\left\{c_{i}^{\left(t\right)}\left(X\right)=c_{i}^{\left(t\right)}\left(x\right)\right\},\Pi_{\nu }^{⊥}X=\Pi_{\nu }^{⊥}x,\text{dir}\left(X^{⊤}\nu \right)=\text{dir}\left(x^{⊤}\nu \right)\right].$$

In (9) $c_{i}^{\left(t\right)}\left(X\right)$ is the cluster assigned to the ith observation at the tth iteration of Algorithm 1. Roughly speaking, this p-value answers the question:

Assuming that there is no difference between the population means of ${\hat{𝒞}}_{1}$ and ${\hat{𝒞}}_{2}$, what is the probability of observing such a large difference between their centroids, among all the realizations of X that yield identical results in every iteration of the k-means algorithm?

The p-value in (9) is the focus of this paper. We establish its key properties below.

**Proposition 1** *Suppose that* x *is a realization from* (1), *and let* $\phi \sim \left(\sigma \|\nu {\|}_{2}\right)\chi_{q}$. *Then*, *under* $H_{0}:\mu^{⊤}\nu =0$ *with* ν *defined in* (3),

(10) $$p_{\text{selective}}=\text{pr}\left[\phi \geq {\left\|x^{⊤}\nu \right\|}_{2}|\overset{T}{\underset{t=0}{\cap }}\overset{n}{\underset{i=1}{\cap }}\left\{c_{i}^{\left(t\right)}\left(x^{\prime }\left(\phi \right)\right)=c_{i}^{\left(t\right)}\left(x\right)\right\}\right],$$

*where $p_{\text{selective }}$ is defined in* (9), *and*

(11) $$x^{\prime }\left(\phi \right)=x+\left(\phi -{\left\|x^{⊤}\nu \right\|}_{2}\right)\left(\nu /\|\nu {\|}_{2}^{2}\right){\left\{\text{dir}\left(x^{⊤}\nu \right)\right\}}^{⊤}.$$

*Moreover*, *the test that rejects* $H_{0}:\mu^{⊤}\nu =0$ *when* $p_{\text{selective}}\leq \alpha$ *controls the selective Type I error at level* α, *in the sense of* (5).

**Figure 2:**
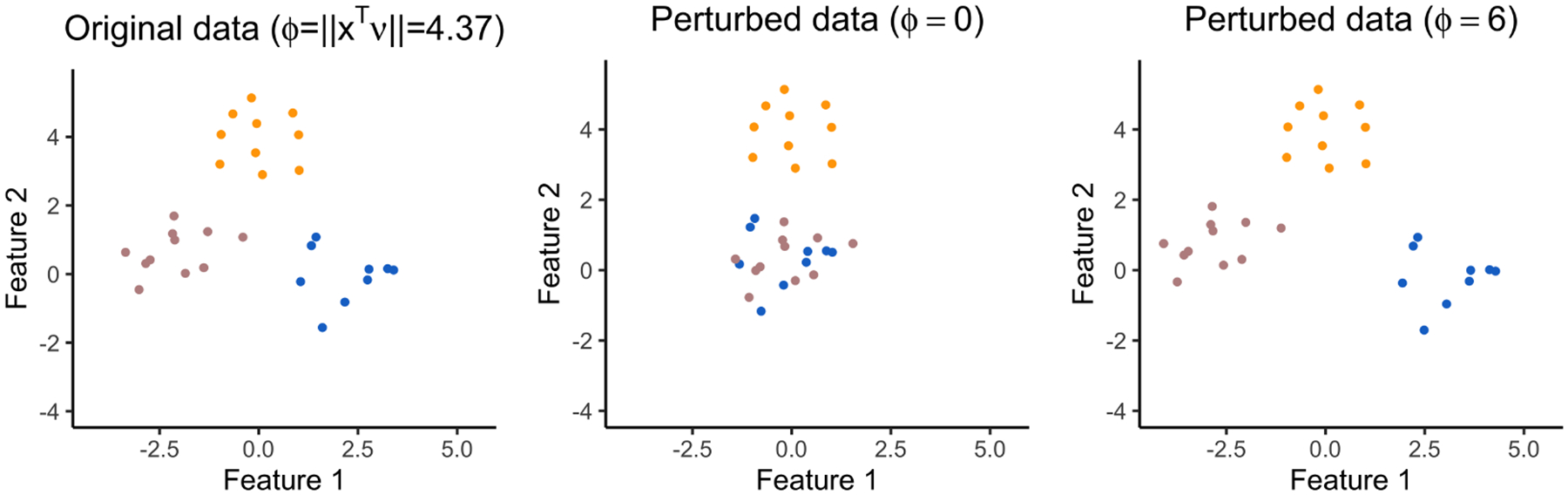
One simulated dataset generated from model (1) with $\mu_{i}=1\left\{1\leq i\leq 10\right\}{\left[2.5,0\right]}^{⊤}+1\left\{11\leq i\leq 20\right\}{\left[0,-2.5\right]}^{⊤}+1\left\{21\leq i\leq 30\right\}{\left[\sqrt{18.75},0\right]}^{⊤}$ and σ=1. *Left*: The original data x corresponds to $\phi ={\left\|x^{⊤}\nu \right\|}_{2}=4.37$. Applying k-means clustering with K=3 yields three clusters, displayed in rosy brown, blue, and orange. Here, ν is chosen to test for a difference in means between ${\hat{𝒞}}_{1}$ (rosy brown) and ${\hat{𝒞}}_{2}$ (blue). *Center*: The perturbed data $x^{\prime }\left(\phi \right)$ with ϕ=0. Applying k-means clustering with K=3 does not yield the same set of clusters as in the left panel. *Right*: The perturbed data $x^{\prime }\left(\phi \right)$ with ϕ=6. Applying k-means clustering with K=3 yields the same set of clusters as in the left panel.

Proposition 1 states that $p_{\text{selective}}$ can be recast as the survival function of a scaled $\chi_{q}$ random variable, truncated to the set

(12) $${𝒮}_{T}=\left\{\phi \in \mathbb{R}:\overset{T}{\underset{t=0}{\cap }}\overset{n}{\underset{i=1}{\cap }}\left\{c_{i}^{\left(t\right)}\left(x^{\prime }\left(\phi \right)\right)=c_{i}^{\left(t\right)}\left(x\right)\right\}\right\},$$

where $x^{\prime }\left(\phi \right)$ is defined in (11). Therefore, to compute $p_{\text{selective}}$, it suffices to characterize the set ${𝒮}_{T}$. In (11), $x^{\prime }\left(\phi \right)$ results from applying a perturbation to the observed data x, along the direction of $x^{⊤}\nu$, the difference between the two cluster centroids of interest. Figure 2 illustrates a realization of (1) for k-means clustering with K=3. The left panel displays the observed data x, which corresponds to $x^{\prime }\left(\phi \right)$ with $\phi ={\left\|x^{⊤}\nu \right\|}_{2}=4.37$. Here, ν defined in (3) was chosen to test the difference between ${\hat{𝒞}}_{1}$ (shown in rosy brown) and ${\hat{𝒞}}_{2}$ (shown in blue). The center and right panels of Figure 2 display $x^{\prime }\left(\phi \right)$ with ϕ=0 and ϕ=6, respectively. In the center panel, with ϕ=0, the blue and rosy brown clusters are “pushed together”, resulting in ${\left\|x^{\prime }{\left(\phi \right)}^{⊤}\nu \right\|}_{2}=0$; that is, there is no difference in empirical means between the two clusters under consideration. Applying k-means clustering no longer results in these clusters. By contrast, in the right panel, with ϕ=6, the blue and rosy brown clusters are “pulled apart” along the direction of $x^{⊤}\nu$, which results in an increased distance between the centroids of the blue and rosy brown clusters, and k-means clustering does yield the same clusters as on the original data. In this example, ${𝒮}_{T}=\left(3.59,\infty \right)$.

## 3. Computation of the selective p-value

In Section 2, we have shown that the p-value $p_{\text{selective}}$ (9) involves the set ${𝒮}_{T}$ in (12). Indeed, a computationally-efficient characterization of ${𝒮}_{T}$ is the key technical challenge and contribution of our paper. Here, we start with a high-level summary of our approach to characterizing ${𝒮}_{T}$ in (12). First, we rewrite

(13) $${𝒮}_{T}=\left\{\phi \in \mathbb{R}:\overset{n}{\underset{i=1}{\cap }}\left\{c_{i}^{\left(0\right)}\left(x^{\prime }\left(\phi \right)\right)=c_{i}^{\left(0\right)}\left(x\right)\right\}\right\}\cap \left\{\phi \in \mathbb{R}:\overset{T}{\underset{t=1}{\cap }}\overset{n}{\underset{i=1}{\cap }}\left\{c_{i}^{\left(t\right)}\left(x^{\prime }\left(\phi \right)\right)=c_{i}^{\left(t\right)}\left(x\right)\right\}\right\}.$$

Next, we consider the first term in (13): according to step 2. of Algorithm 1, for i=1, … ,n, $c_{i}^{\left(0\right)}\left(x^{\prime }\left(\phi \right)\right)=c_{i}^{\left(0\right)}\left(x\right)$ if and only if for i=1, … ,n, the initial randomly-sampled centroid to which ${\left[x^{\prime }\left(\phi \right)\right]}_{i}$ is closest coincides with the initial centroid to which $x_{i}$ is closest. This condition can be expressed using K−1 inequalities. Furthermore, the same intuition holds for the second term in (13), except that the centroids are a function of the cluster assignments in the previous iteration. We formalize this intuition in Proposition 2, proven in Appendix A.2.

**Proposition 2** *Suppose that we apply the k-means clustering algorithm* (Algorithm 1) *to a matrix* $x\in {\mathbb{R}}^{n\times q}$, *to obtain* K *clusters in at most* T *steps*. *Define*

(14) $$w_{i}^{\left(t\right)}\left(k\right)=1\left\{c_{i}^{\left(t\right)}\left(x\right)=k\right\}/\overset{n}{\underset{i^{\prime }=1}{\sum }}1\left\{c_{i^{\prime }}^{\left(t\right)}\left(x\right)=k\right\}.$$

*Then*, *for the set* ${𝒮}_{T}$ *defined in* (12), *we have that*

(15) $${𝒮}_{T}=\left(\overset{n}{\underset{i=1}{\cap }}\overset{K}{\underset{k=1}{\cap }}\left\{\phi :{\left\|{\left[x^{\prime }\left(\phi \right)\right]}_{i}-m_{c_{i}^{\left(0\right)}\left(x\right)}^{\left(0\right)}\left(x^{\prime }\left(\phi \right)\right)\right\|}_{2}^{2}\leq {\left\|{\left[x^{\prime }\left(\phi \right)\right]}_{i}-m_{k}^{\left(0\right)}\left(x^{\prime }\left(\phi \right)\right)\right\|}_{2}^{2}\right\}\right)\cap$$

(16) $$\left(\overset{T}{\underset{t=1}{\cap }}\overset{n}{\underset{i=1}{\cap }}\overset{K}{\underset{k=1}{\cap }}\left\{\phi :{\left\|{\left[x^{\prime }(\phi )\right]}_{i}-\sum_{i^{\prime }=1}^{n}w_{i^{\prime }}^{\left(t-1\right)}\left(c_{i}^{\left(t\right)}(x)\right){\left[x^{\prime }(\phi )\right]}_{i^{\prime }}\right\|}_{2}^{2}\leq {\left\|{\left[x^{\prime }(\phi )\right]}_{i}-\sum_{i^{\prime }=1}^{n}w_{i^{\prime }}^{\left(t-1\right)}(k){\left[x^{\prime }(\phi )\right]}_{i^{\prime }}\right\|}_{2}^{2}\right\}\right).$$

Recall that $c_{i}^{\left(t\right)}\left(x\right)$ denotes the cluster to which the ith observation is assigned in step 3b. of Algorithm 1 during the tth iteration, and that $m_{k}^{\left(0\right)}\left(x\right)$ denotes the kth centroid sampled from the data x during step 1 of Algorithm 1. In words, Proposition 2 says that ${𝒮}_{T}$ can be expressed as the intersection of 𝒪(nKT) sets. Therefore, it suffices to characterize the sets in (15) and (16). We now present two lemmas.

**Lemma 3 (Lemma 2 in** Gao et al. (2022)) *For* ν *in* (3) *and* $x^{\prime }\left(\phi \right)$ *in* (11), *we have that* ${\left\|{\left[x^{\prime }\left(\phi \right)\right]}_{i}-{\left[x^{\prime }\left(\phi \right)\right]}_{j}\right\|}_{2}^{2}=a\phi^{2}+b\phi +\gamma$, *where* $a={\left\{\left(\nu_{i}-\nu_{j}\right)/\|\nu {\|}_{2}^{2}\right\}}^{2},b=2[\left(\nu_{i}-\nu_{j}\right)/\|\nu {\|}_{2}^{2}x_{i}-x_{j},\text{dir}\left(x^{⊤}\nu \right)-{\left\{\left(\nu_{i}-\nu_{j}\right)/\|\nu {\|}_{2}^{2}\right\}}^{2}{\left\|x^{⊤}\nu \right\|}_{2}]$, *and* $\gamma ={\left\|x_{i}-x_{j}-\left(\nu_{i}-\nu_{j}\right)\left(x^{⊤}\nu \right)/\left\|\nu \right\|{\;}_{2}^{2}\right\|}_{2}^{2}$.

**Lemma 4** *For* ν *in* (3), $x^{\prime }\left(\phi \right)$ *in* (11), *and* $w_{i}^{\left(t\right)}\left(k\right)$ *in* (14), *we have that* ${\left\|{\left[x^{\prime }(\phi )\right]}_{i}-\sum_{i^{\prime }=1}^{n}w_{i^{\prime }}^{\left(t-1\right)}(k){\left[x^{\prime }(\phi )\right]}_{i^{\prime }}\right\|}_{2}^{2}=\tilde{a}\phi^{2}+\tilde{b}\phi +\tilde{\gamma }$ , *where* $\tilde{a}={\left(\nu_{i}-\sum_{i^{\prime }=1}^{n}w_{i^{\prime }}^{\left(t-1\right)}(k)\nu_{i^{\prime }}\right)}^{2}/\|\nu {\|}_{2}^{4}$ , $\tilde{b}=\left(2/\|\nu {\|}_{2}^{2}\right)\left\{\left(\nu_{i}-\sum_{i^{\prime }=1}^{n}w_{i^{\prime }}^{\left(t-1\right)}(k)\nu_{i^{\prime }}\right)\langle x_{i}-\sum_{i^{\prime }=1}^{n}w_{i^{\prime }}^{\left(t-1\right)}(k)x_{i^{\prime }},\text{ dir}\left(x^{⊤}\nu \right)\rangle -{\left(\nu_{i}-\sum_{i^{\prime }=1}^{n}w_{i^{\prime }}^{\left(t-1\right)}(k)\nu_{i^{\prime }}\right)}^{2}\left(x^{⊤}\nu_{2}\right)/\|\nu {\|}_{2}^{4}\right\}$ , $\tilde{\gamma }={\left\|x_{i}-\sum_{i^{\prime }=1}^{n}w_{i^{\prime }}^{\left(t-1\right)}(k)x_{i^{\prime }}-\left(\nu_{i}-\sum_{i^{\prime }=1}^{n}w_{i^{\prime }}^{\left(t-1\right)}(k)\nu_{i^{\prime }}\right)\left(x^{⊤}\nu \right)/{\left\|\nu \right\|}_{2}^{2}\right\|}_{2}^{2}$.

It follows from Lemmas 3 and 4 that all of the inequalities in (15) and (16) are in fact *quadratic* in ϕ, with coefficients that can be analytically computed. Therefore, computing the set ${𝒮}_{T}$ requires solving 𝒪(nKT) quadratic inequalities of ϕ.

**Proposition 5** *Suppose that we apply the k-means clustering algorithm* (Algorithm 1) *to a matrix* $x\in {\mathbb{R}}^{n\times q}$, *to obtain* K *clusters in at most* T *steps*. *Then*, *the set* ${𝒮}_{T}$ *defined in* (12) *can be computed in* 𝒪(KT(n+q)+nKT log(nKT)) *operations*.

## 4. Extensions

### 4.1 Non-spherical covariance matrix

Thus far, we have assumed that the observed data x is a realization of (1), which implies that $\text{cov}\left(X_{i}\right)=\sigma^{2}I_{q}$. However, this assumption is often violated in practice. For example, expression levels of genes are highly correlated, and neighbouring pixels in an image tend to be more similar. For a known positive definite matrix Σ, we now let

(17) $$X\sim ℳ{𝒩}_{n\times q}\left(\mu ,{\text{I}}_{n},\Sigma \right).$$

Under (17), we can whiten the data by applying the transformation $x_{i}\to \Sigma^{-\frac{1}{2}}x_{i}$ (Bell and Sejnowski, 1997), where $\Sigma^{-\frac{1}{2}}$ is the unique symmetric positive definite square root of $\Sigma^{-1}$ (Horn and Johnson, 2012). Note that $\Sigma^{-\frac{1}{2}}X_{i}\sim 𝒩\left(\Sigma^{-\frac{1}{2}}\mu_{i},{\text{I}}_{q}\right)$. Moreover, as $\Sigma^{-\frac{1}{2}}≻0$, testing the null hypothesis in (2) is equivalent to testing

(18) $$H_{0}:\underset{i\in {\hat{𝒞}}_{1}}{\sum }\Sigma^{-\frac{1}{2}}\mu_{i}/\left|{\hat{𝒞}}_{1}\right|=\underset{i\in {\hat{𝒞}}_{2}}{\sum }\Sigma^{-\frac{1}{2}}\mu_{i}/\left|{\hat{𝒞}}_{2}\right|\text{ versus }H_{1}:\underset{i\in {\hat{𝒞}}_{1}}{\sum }\Sigma^{-\frac{1}{2}}\mu_{i}/\left|{\hat{𝒞}}_{1}\right|\neq \underset{i\in {\hat{𝒞}}_{2}}{\sum }\Sigma^{-\frac{1}{2}}\mu_{i}/\left|{\hat{𝒞}}_{2}\right|.$$

Therefore, to get a correctly-sized test under model (17), we can simply carry out our proposal in Section 2 on the transformed data $\Sigma^{-\frac{1}{2}}x_{i}$ instead of the original data $x_{i}$.

Instead of applying the whitening transformation, we can directly accommodate a known covariance matrix Σ by considering the following extension of $p_{\text{selective}}$ in (9):

(19) $$p_{\Sigma ,\text{selective}}={\text{pr}}_{H_{0}}\left[{\left\|\Sigma^{-\frac{1}{2}}X^{⊤}\nu \right\|}_{2}\geq {\left\|\Sigma^{-\frac{1}{2}}x^{⊤}\nu \right\|}_{2}|\overset{T}{\underset{t=0}{\cap }}\overset{n}{\underset{i=1}{\cap }}\left\{c_{i}^{\left(t\right)}\left(X\right)=c_{i}^{\left(t\right)}\left(x\right)\right\},\Pi_{\nu }^{⊥}X=\Pi_{\nu }^{⊥}x,\text{dir}\left(\Sigma^{-\frac{1}{2}}X^{⊤}\nu \right)=\text{dir}\left(\Sigma^{-\frac{1}{2}}x^{⊤}\nu \right)\right].$$

**Proposition 6** *Suppose that* x *is a realization from* (17), *and let* $\phi \sim \left(\|\nu {\|}_{2}\right)\chi_{q}$. *Then*, *under* $H_{0}:\mu^{⊤}\nu =0$ *with* ν *defined in* (3),

(20) $$p_{\Sigma ,\text{selective}}=\text{pr}\left[\phi \geq {\left\|\Sigma^{-\frac{1}{2}}x^{⊤}\nu \right\|}_{2}|\overset{T}{\underset{t=0}{\cap }}\overset{n}{\underset{i=1}{\cap }}\left\{c_{i}^{\left(t\right)}\left(\Pi_{\nu }^{⊥}x+\left(\phi \frac{\nu }{\|\nu {\|}_{2}^{2}}\right){\left\{\text{dir}\left(\Sigma^{-\frac{1}{2}}x^{⊤}\nu \right)\right\}}^{⊤}\Sigma^{\frac{1}{2}}\right)=c_{i}^{\left(t\right)}\left(x\right)\right\}\right]\text{,}$$

*where* $p_{\Sigma ,\text{selective}}$ *is defined in* (19). *Furthermore*, *the test that rejects* $H_{0}:\mu^{⊤}\nu =0$ *when* $p_{\Sigma ,\text{selective}}\leq \alpha$ *controls the selective Type I error at level* α.

In addition, we can adapt the results in Section 3 to compute the set $\left\{\phi \in \mathbb{R}:\cap_{t=0}^{T}\cap_{i=1}^{n}\left\{c_{i}^{\left(t\right)}\left(\Pi_{\nu }^{⊥}x+\left(\phi \nu /\|\nu {\|}_{2}^{2}\right){\left\{\text{dir}\left(\Sigma^{-\frac{1}{2}}x^{⊤}\nu \right)\right\}}^{⊤}\Sigma^{\frac{1}{2}}\right)=c_{i}^{\left(t\right)}\left(x\right)\right\}\right\}$ by modifying the results in Lemmas 3 and 4. Details are in Section A.5 of the Appendix.

### 4.2 Unknown variance

When σ is unknown, we can plug in an estimate $\hat{\sigma }$ in (9):

(21) $${\hat{p}}_{\text{selective}}\left(\hat{\sigma }\right)=\text{pr}\left[\phi \left(\hat{\sigma }\right)\geq {\left\|x^{⊤}\nu \right\|}_{2}|\overset{T}{\underset{t=0}{\cap }}\overset{n}{\underset{i=1}{\cap }}\left\{c_{i}^{\left(t\right)}\left(x^{\prime }\left(\phi \left(\hat{\sigma }\right)\right)\right)=c_{i}^{\left(t\right)}\left(x\right)\right\}\right],$$

where $\phi \left(\hat{\sigma }\right)\sim \left(\hat{\sigma }\|\nu {\|}_{2}\right)\chi_{q}$. If we use a consistent estimator of σ, then a test based on the p-value in (21) provides selective Type I error control (5) asymptotically.

**Proposition 7** *For* q=1,2, … , *suppose that* $X^{\left(q\right)}\sim ℳ{𝒩}_{n\times q}\left(\mu^{\left(q\right)},I_{n},\sigma^{2}I_{q}\right)$. *Let* $x^{\left(q\right)}$ *be a realization from* $X^{\left(q\right)}$ *and let* $c_{i}^{\left(t\right)}\left(\cdot \right)$ *be the cluster to which the* i *th observation is assigned during the tth iteration of step 3b*. *in* Algorithm 1. *Consider the sequence of null hypotheses* $H_{0}^{\left(q\right)}:\mu^{{\left(q\right)}^{⊤}}\nu^{\left(q\right)}=0_{q}$ , *where* $\nu^{\left(q\right)}$ *defined in* (3) *is the contrast vector resulting from applying* k-*means clustering on* $x^{\left(q\right)}$. *Suppose that* (*i*) $\hat{\sigma }$ *is a consistent estimator of* σ, *i.e*., *for all* ϵ>0, ${\text{lim}}_{q\to \infty }\text{pr}\left(\left|\hat{\sigma }\left(X^{\left(q\right)}\right)-\sigma \right|\geq \epsilon \right)=0$; *and (ii) there exists* δ∈(0,1) *such that* ${\text{lim}}_{q\to \infty }\;{\text{pr}}_{H_{0}^{\left(q\right)}}\left[\cap_{t=0}^{T}\cap_{i=1}^{n}\left\{c_{i}^{\left(t\right)}\left(X^{\left(q\right)}\right)=c_{i}^{\left(t\right)}\left(x^{\left(q\right)}\right)\right\}\right]>\delta$. *Then, for all* α∈(0,1), *we have that* ${\text{lim}}_{q\to \infty }{\text{pr}}_{H_{0}^{\left(q\right)}}\left[{\hat{p}}_{\text{selective}}\left(\hat{\sigma }\right)\leq \alpha |\cap_{t=0}^{T}\cap_{i=1}^{n}\left\{c_{i}^{\left(t\right)}\left(X^{\left(q\right)}\right)=c_{i}^{\left(t\right)}\left(x^{\left(q\right)}\right)\right\}\right]=\alpha$.

In practice, we propose to use the following estimator of σ (Huber, 1981):

(22) $${\hat{\sigma }}_{\text{MED}}\left(x\right)={\left\{\underset{1\leq i\leq n,1\leq j\leq q}{\text{median}}\left({\tilde{x}}_{ij}^{2}\right)/M_{\chi_{1}^{2}}\right\}}^{1/2},$$

where $\tilde{x}$ is obtained from subtracting the median of each column in x, and $M_{\chi_{1}^{2}}$ is the median of the $\chi_{1}^{2}$ distribution. If μ is sparse, i.e., $\sum_{i=1}^{n}\sum_{j=1}^{q}1\left\{\mu_{ij}\neq 0\right\}$ is small, then (22) is consistent with appropriate assumptions; see Appendix A.7.

## 5. Simulation study

Throughout this section, we consider testing the null hypothesis $H_{0}:\mu^{⊤}\nu =0_{q}$ versus $H_{1}:\mu^{⊤}\nu \neq 0_{q}$, where, unless otherwise stated, ν defined in (3) is based on a randomly-chosen pair of clusters ${\hat{𝒞}}_{1}$ and ${\hat{𝒞}}_{2}$ from k-means clustering. We consider four p-values: $p_{\text{Naive}}$ in (4), $p_{\text{selective}}$ in (9), ${\hat{p}}_{\text{selective}}$ in (21) with ${\hat{\sigma }}_{\text{MED}}$ defined in (22), and ${\hat{p}}_{\text{selective}}$ in (21) with ${\hat{\sigma }}_{\text{Sample}}={\left\{\sum_{i=1}^{n}\sum_{j=1}^{q}{\left(x_{ij}-{\bar{x}}_{j}\right)}^{2}/\left(nq-q\right)\right\}}^{1/2}$, where ${\bar{x}}_{j}=\sum_{i=1}^{n}x_{ij}/n$. In the simulations that follow, we compare the selective Type I error (5) and power of the tests that reject $H_{0}$ when these p-values are less than α=0.05.

### 5.1. Selective Type I error under the global null

We generate data from (1) with $\mu =0_{n\times q}$; therefore, $H_{0}$ in (2) holds for any pair of estimated clusters. We simulate 3,000 datasets with n=150, σ=1, and q=2,10,50,100.

**Figure 3:**
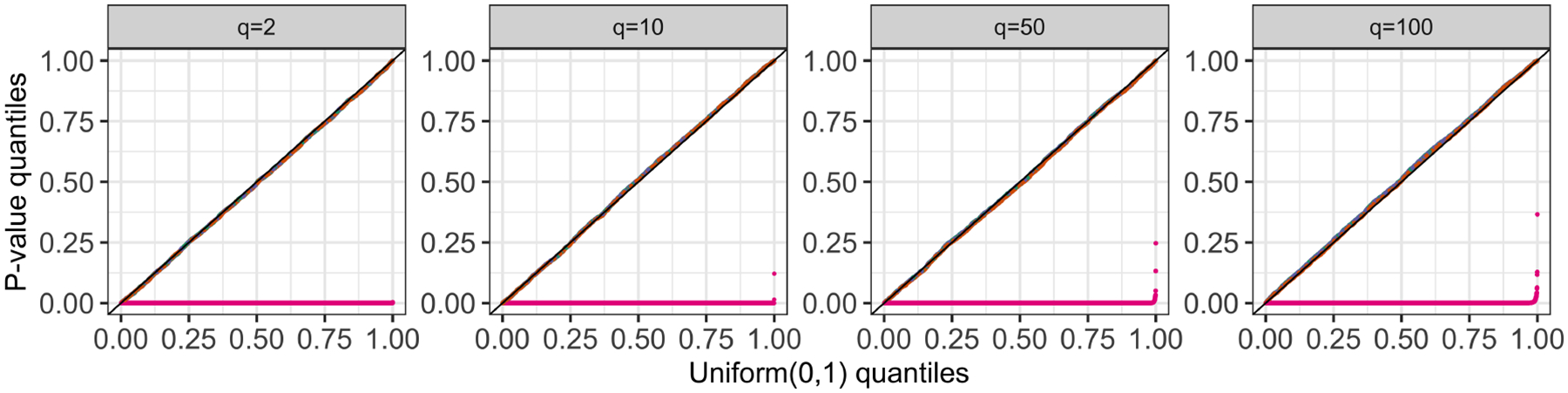
Quantile-quantile plots for $p_{\text{Naive}}$ (pink), $p_{\text{selective}}$ (green), ${\hat{p}}_{\text{selective}}\left({\hat{\sigma }}_{\text{MED}}\right)$ (orange), and ${\hat{p}}_{\text{selective}}\left({\hat{\sigma }}_{\text{Sample}}\right)$ (purple) under (1) with $\mu =0_{n\times q}$, stratified by q.

For each simulated dataset, we apply k-means clustering with K=3 , and then compute $p_{\text{Naive}}$, $p_{\text{selective}}$, ${\hat{p}}_{\text{selective}}\left({\hat{\sigma }}_{\text{MED}}\right)$ and ${\hat{p}}_{\text{selective}}\left({\hat{\sigma }}_{\text{Sample}}\right)$ for a randomly-chosen pair of clusters. Figure 3 displays the observed p-value quantiles versus the Uniform (0, 1) quantiles. We see that for all values of q, (i) the naive p-values in (4) are stochastically smaller than a Uniform (0, 1) random variable, and the test based on $p_{\text{Naive}}$ leads to an inflated Type I error rate; (ii) tests based $p_{\text{selective}}$, ${\hat{p}}_{\text{selective}}\left({\hat{\sigma }}_{\text{MED}}\right)$, and ${\hat{p}}_{\text{selective}}\left({\hat{\sigma }}_{\text{Sample}}\right)$ control the selective Type I error rate in the sense of (5).

### 5.2 Conditional power and detection probability

In this section, we show that the tests based on our proposal ($p_{\text{selective }}$, ${\hat{p}}_{\text{selective}}\left({\hat{\sigma }}_{\text{MED }}\right)$, and ${\hat{p}}_{\text{selective}}\left({\hat{\sigma }}_{\text{Sample}}\right)$) have substantial power to reject $H_{0}$ when it is not true. We generate data from (1) with n=150 and

(23) $$\mu_{1}=\;\ldots \;=\mu_{\frac{n}{3}}=\left[\begin{array}{c} -\frac{\delta }{2}\\ 0_{q-1} \end{array}\right],\mu_{\frac{n}{3}+1}=\;\ldots \;=\mu_{\frac{2n}{3}}=\left[\begin{array}{c} 0_{q-1}\\ \frac{\sqrt{3\delta }}{2} \end{array}\right],\mu_{\frac{2n}{3}+1}=\;\ldots \;=\mu_{n}=\left[\begin{array}{c} \frac{\delta }{2}\\ 0_{q-1} \end{array}\right].$$

Here, we can think of ${𝒞}_{1}=\left\{1,\;\ldots \;,n/3\right\}$, ${𝒞}_{2}=\left\{\left(n/3\right)+1,\;\ldots \;,\left(2n/3\right)\right\}$, ${𝒞}_{3}=\left\{\left(2n/3\right)+1,\;\ldots \;,n\right\}$ as the “true clusters”. Moreover, these clusters are equidistant in the sense that the pairwise distance between each pair of population means is |δ|. Recall that we test $H_{0}$ in (2) for a pair of estimated clusters ${\hat{𝒞}}_{1}$ and ${\hat{𝒞}}_{2}$, which may not be true clusters. Hence, we will separately consider the *conditional power* and *detection probability* of our proposed tests (Gao et al., 2022; Jewell et al., 2022; Hyun et al., 2021). The conditional power is the probability of rejecting $H_{0}$ in (2), given that ${\hat{𝒞}}_{1}$ and ${\hat{𝒞}}_{2}$ are true clusters. Given M simulated datasets with true clusters $\left\{{𝒞}_{1},\;\ldots \;,{𝒞}_{L}\right\}$, we estimate it as

(24) $$\text{Conditional power }=\frac{\sum_{m=1}^{M}1\left\{\left\{{\hat{𝒞}}_{1}^{\left(m\right)},{\hat{𝒞}}_{2}^{\left(m\right)}\right\}\subseteq \left\{{𝒞}_{1},\;\ldots \;,{𝒞}_{L}\right\},p^{\left(m\right)}\leq \alpha \right\}}{\sum_{m=1}^{M}1\left\{\left\{{\hat{𝒞}}_{1}^{\left(m\right)},{\hat{𝒞}}_{2}^{\left(m\right)}\right\}\subseteq \left\{{𝒞}_{1},\;\ldots \;,{𝒞}_{L}\right\}\right\}},$$

where, and $p^{\left(m\right)}$ and ${\hat{𝒞}}_{1}^{\left(m\right)}$, ${\hat{𝒞}}_{2}^{\left(m\right)}$ correspond to the p-value and clusters under consideration for the mth simulated dataset. Because the quantity in (24) conditions on the event that ${\hat{𝒞}}_{1}$ and ${\hat{𝒞}}_{2}$ are true clusters, we also estimate how often that event occurs:

(25) $$\text{Detection probability }=\overset{M}{\underset{m=1}{\sum }}1\left\{\left\{{\hat{𝒞}}_{1}^{\left(m\right)},{\hat{𝒞}}_{2}^{\left(m\right)}\right\}\subseteq \left\{{𝒞}_{1},\;\ldots \;,{𝒞}_{L}\right\}\right\}/M.$$

**Figure 4:**
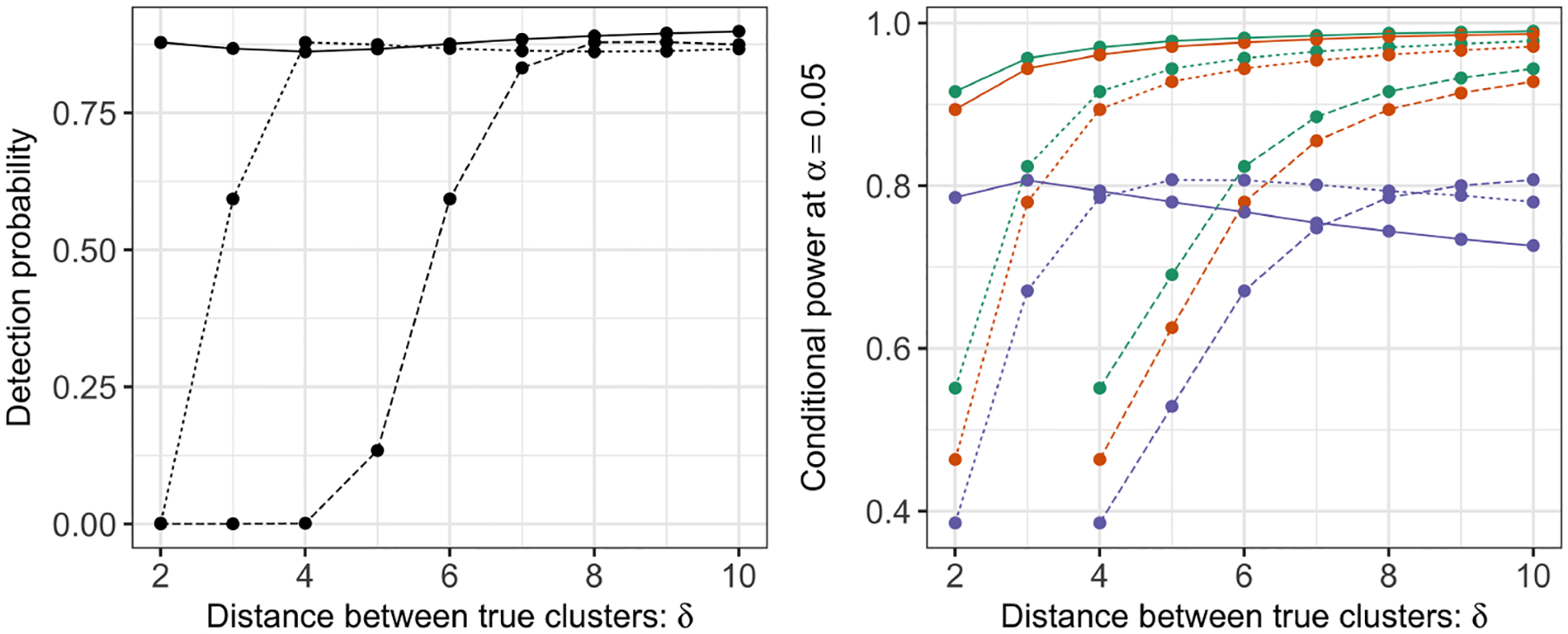
Left: The detection probability (25) for k-means clustering with K=3 under model (1) with μ defined in (23), and σ=0.25 (solid lines), 0.5 (dashed lines), and 1 (long-dashed lines). *Right*: The conditional power (24) at α=0.05 for the tests based on $p_{\text{selective }}$ (green), ${\hat{p}}_{\text{selective}}\left({\hat{\sigma }}_{\text{MED}}\right)$ (orange), and ${\hat{p}}_{\text{selective}}\left({\hat{\sigma }}_{\text{Sample}}\right)$ (purple), under model (1) with μ defined in (23) and σ=0.25,0.5,1. The conditional power is not displayed for δ=2,3,σ=1 because the true clusters were never recovered in simulation.

We generate M=200,000 datasets from (23) with q=10, σ=0.25,0.5,1, and δ=2,3, … ,10. For each simulated dataset, we apply k-means clustering with K=3 and reject $H_{0}:\mu^{⊤}\nu =0_{q}$ if $p_{\text{selective }}$, ${\hat{p}}_{\text{selective}}\left({\hat{\sigma }}_{\text{MED}}\right)$, or ${\hat{p}}_{\text{selective}}\left({\hat{\sigma }}_{\text{Sample}}\right)$ is less than α=0.05. In Figure 4, the left panel displays the detection probability (25) of k-means clustering as a function of δ in (23), and the right panel displays the conditional power (24) for the tests based on $p_{\text{selective}}$, ${\hat{p}}_{\text{selective}}\left({\hat{\sigma }}_{\text{MED}}\right)$, and ${\hat{p}}_{\text{selective}}\left({\hat{\sigma }}_{\text{Sample}}\right)$. Under model (1), the detection probability and conditional power increase as a function of δ in (23) for all values of σ. For a given value of δ, a larger value of σ leads to lower detection probability and conditional power. The conditional power is not displayed for δ=2,3, σ=1 because the true clusters were never recovered in simulation. Moreover, for a given value of δ and σ, the test based on $p_{\text{selective}}$ has the highest conditional power, followed closely by the test based on ${\hat{p}}_{\text{selective}}\left({\hat{\sigma }}_{\text{MED}}\right)$. Using ${\hat{\sigma }}_{\text{Sample}}$ in ${\hat{p}}_{\text{selective}}$ leads to a less powerful test, especially for large values of δ. This is because ${\hat{\sigma }}_{\text{Sample}}$ is a conservative estimator of σ in (1), and its bias is an increasing function of δ, the distance between true clusters. By contrast, ${\hat{\sigma }}_{\text{MED}}$ is a consistent estimator under model (23) (see Appendix A.7).

As an alternative to the conditional power in (24), in Appendix A.8, we consider a notion of power that does not condition on having correctly estimated the true clusters.

## 6. Real data applications

### 6.1 MNIST Dataset (Lecun et al., 1998)

Here, we apply our method to the MNIST dataset (Lecun et al., 1998), which consists of 60,000 gray-scale images of handwritten digits. Each image has an accompanying label in {0, 1, …, 9}, and is stored as a 28 × 28 matrix that takes on values in [0, 255]. We first divide the entries of all the images by 255. Next, since there is no variation in the peripheral pixels of the images (Gallaugher and McNicholas, 2018), which violates model (1), we add an independent perturbation 𝒩(0,0.01) to each element of the image. Finally, we vectorize each image to obtain a vector $x_{i}\in {\mathbb{R}}^{784}$.

We first construct a “no cluster” dataset by randomly sampling 1,500 images of the 0s; thus, n=1,500 and q=784. To de-correlate the pixels in each image, we whitened the data (see Section 4.1) using ${\hat{\Sigma }}^{-\frac{1}{2}}=U{\left(\Lambda +0.01I_{n}\right)}^{-\frac{1}{2}}U^{⊤}$ as in prior work (Coates and Ng, 2012), where $U\Lambda U^{⊤}$ is the eigenvalue decomposition of the sample covariance matrix.

**Figure 5:**
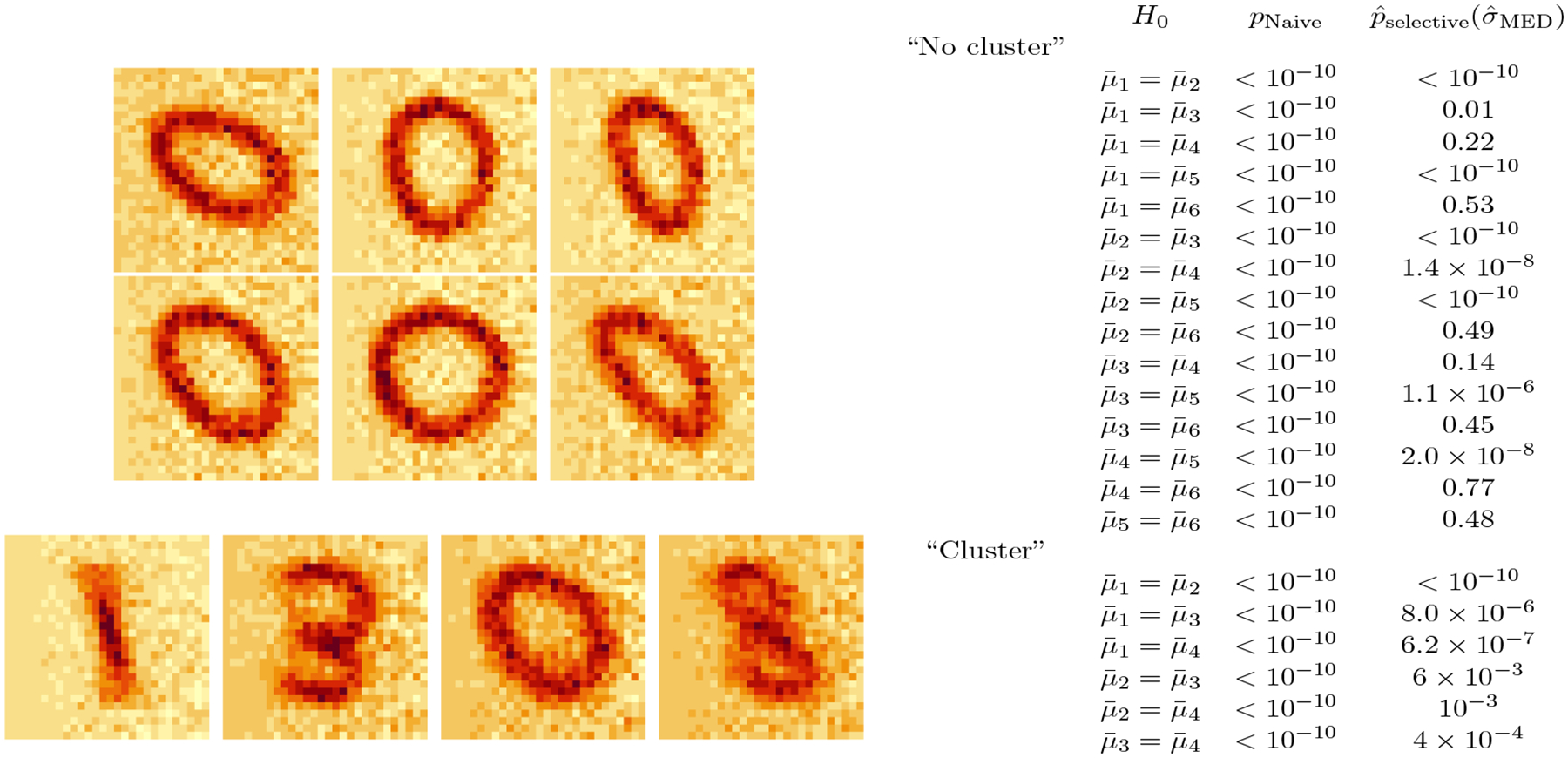
*Top left*: Centroids of six clusters from the “no cluster” dataset ${\hat{𝒞}}_{1}$ to ${\hat{𝒞}}_{6}$ from left to right, top to bottom). *Bottom left*: Same as top left, but for the “cluster” dataset. *Right*: We test the null hypothesis of no difference between each pair of cluster centroids using $p_{\text{Naive}}$ and ${\hat{p}}_{\text{selective}}\left({\hat{\sigma }}_{\text{MED}}\right)$. Here, ${\bar{\mu }}_{i}=\sum_{j\in {\hat{𝒞}}_{i}}\mu_{j}/\left|{\hat{𝒞}}_{i}\right|$.

We apply k-means clustering with K=6. The centroids are displayed in the top left panel of Figure 5. For each pair of estimated clusters, we compute the p-values $p_{\text{Naive}}$ and ${\hat{p}}_{\text{selective}}\left({\hat{\sigma }}_{\text{MED}}\right)$ (see Figure 5). The naïve p-values are extremely small for all pairs of clusters under consideration, despite the resemblance of the centroids. By contrast, our approach yields modest p-values, congruent with the visual resemblance of the centroids. In addition, for the most part, the pairs for which ${\hat{p}}_{\text{selective}}\left({\hat{\sigma }}_{\text{MED}}\right)$ is small are visually quite different (e.g., clusters 1 and 2, clusters 1 and 5, and clusters 4 and 5).

To demonstrate the power of the test based on ${\hat{p}}_{\text{selective}}\left({\hat{\sigma }}_{\text{MED}}\right)$, we also generated a “cluster” dataset by sampling 500 images each from digits 0, 1, 3, and 8 ; thus, n=2,000 and q=784. We again whitened the data to obtain uncorrelated features. After applying k-means clustering with K=4, we obtain four clusters that roughly correspond to four digits: cluster 1, 94.0% digit 1; cluster 2, 72.4% digit 3; cluster 3, 83.6% digit 0; cluster 4, 62.4% digit 8 (see the bottom left panel of Figure 5). Results from testing for a difference in means for each pair of clusters using $p_{\text{Naive}}$ and ${\hat{p}}_{\text{selective}}\left({\hat{\sigma }}_{\text{MED}}\right)$ are in Figure 5. Both sets of p-values are small on this “cluster” dataset.

### 6.2 Single-cell RNA-sequencing data (Zheng et al., 2017)

In this section, we apply our proposal to single-cell RNA-sequencing data collected by Zheng et al. (2017). Single-cell RNA-sequencing quantifies gene expression abundance at the resolution of single cells, thereby revealing cell-to-cell heterogeneity in transcription and allowing for the identification of cell types and marker genes. In practice, biologists often cluster the cells to identify putative cell types, and then perform a differential expression analysis, i.e., they test for a difference in gene expression between two clusters (Stuart et al., 2019; Lähnemann et al., 2020; Grün et al., 2015). Because this approach ignores the fact that the clusters were estimated from the same data used for testing, it does not control the selective Type I error.

Zheng et al. (2017) profiled 68,000 peripheral blood mononuclear cells, and classified them based on their match to the expression profiles of 11 reference transcriptomes from known cell types. We consider the classified cell types to be the “ground truth”, and use this information to demonstrate that our proposal in Section 2 yields reasonable results.

As in prior work (Gao et al., 2022; Duò et al., 2018), we first excluded cells with low numbers of expressed genes or total counts, as well as cells in which a large percentage of the expressed genes are mitochondrial. We then divided the counts for each cell by the total sum of counts in that cell. Finally, we applied a log_2_ transformation with a pseudo-count of 1 to the expression data, and considered only the subset of 500 genes with the largest average expression levels pre-normalization. We applied the aforementioned pre-processing pipeline separately to memory T cells (N=10,224) and a mixture of five types of cells (memory T cells, B cells, naive T cells, natural killer cells, and monocytes; N=43,259).

To investigate the selective Type I error in the absence of true clusters, we first constructed a “no cluster” dataset by randomly sampling 1,000 out of 10,224 memory T cells after pre-processing (thus, n=1,000 and q=500). Since the gene expression levels are highly correlated, we first whitened the data as described in Section 4.1 by plugging in ${\hat{\Sigma }}^{-\frac{1}{2}}=U{\left(\Lambda +0.01I_{n}\right)}^{-\frac{1}{2}}U^{⊤}$ (Coates and Ng, 2012), where $U\Lambda U^{⊤}$ is the eigenvalue decomposition of the sample covariance matrix.

**Figure 8:**
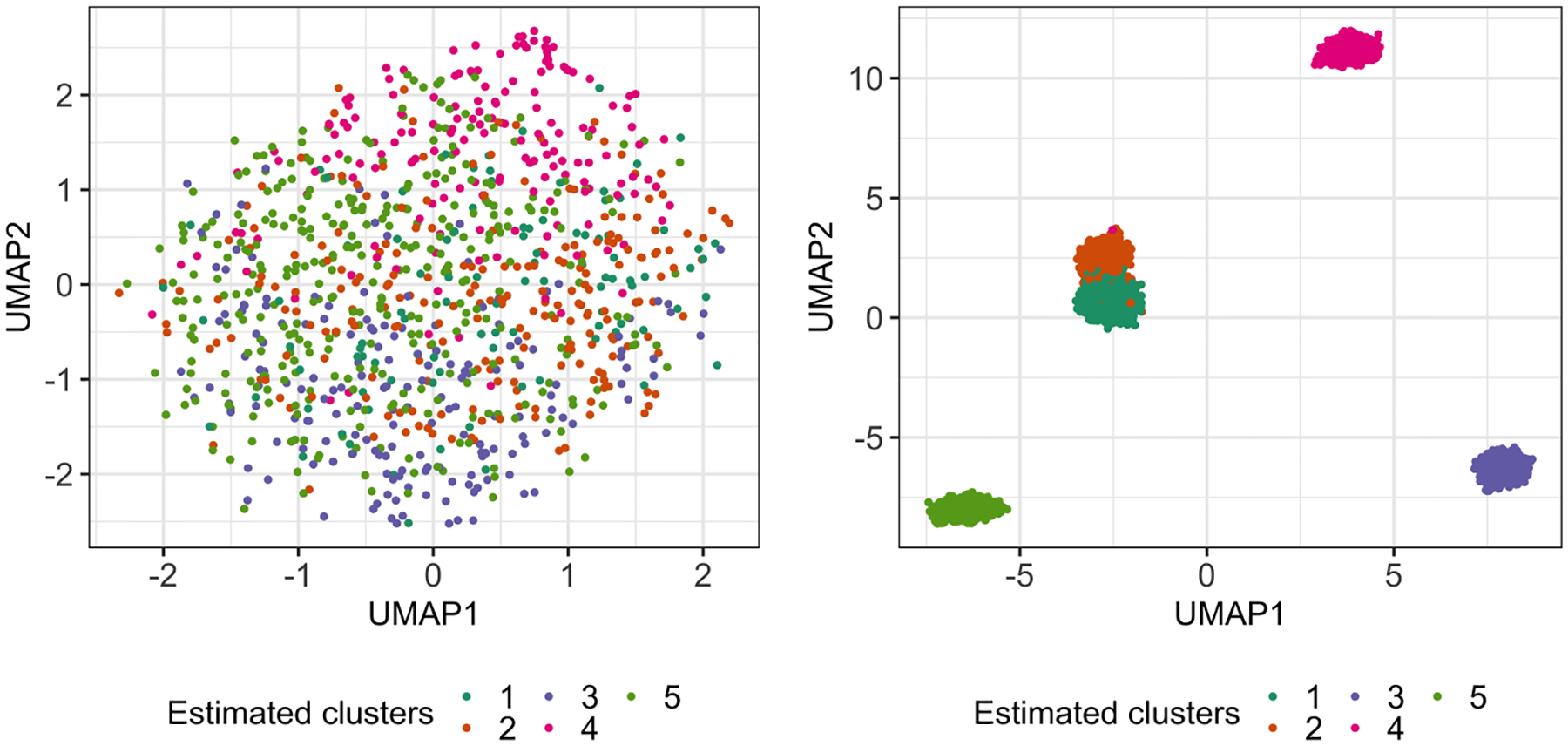
*Left*: The two-dimensional UMAP embedding (McInnes et al., 2018) of the “no cluster” dataset after preprocessing (as described in Section 6.2), colored by the estimated cluster membership via k-means clustering. *Right*: Same as left, but for the “cluster” dataset.

We applied k-means clustering to the transformed data with K=5, and obtained five clusters consisting of 97, 223, 172,165, and 343 cells, respectively (see Figure 8 left panel in Appendix A.9). For each pair of estimated clusters, we computed the p-values $p_{\text{Naive}}$ and ${\hat{p}}_{\text{selective}}\left({\hat{\sigma }}_{\text{MED}}\right)$. The results are displayed in the top panel of Table 1. On this dataset, the naive p-values are extremely small for all pairs of estimated clusters, while our proposed p-values are quite large. In particular, at α=0.05, the test based on $p_{\text{Naive}}$ concludes that all five estimated clusters correspond to distinct cell types (even after multiplicity correction). By contrast, our approach does not reject most of the null hypotheses; i.e., it finds no difference between expression levels of the estimated clusters. Because this “no cluster” dataset consists only of memory T cells, we believe that conclusion based on ${\hat{p}}_{\text{selective}}\left({\hat{\sigma }}_{\text{MED}}\right)$ aligns better with the underlying biology.

Next, we construct a “cluster” dataset by randomly sampling 400 each of memory T cells, B cells, naive T cells, natural killer cells, and monocytes from the 43, 259 cells; thus, n=2,000 and q=500. After whitening the data, we applied k-means clustering to obtain five clusters. We see that these clusters approximately correspond to the five different cell types (cluster 1: 82.5% naive T cells; cluster 2: 95.3% memory T cells; cluster 3: 99.2% B cells; cluster 4: 91.5% nature killer cells; cluster 5: 83.3% monocytes); estimated clusters are visualized in the right panel of Figure 8 in Appendix A.9. We evaluate the p-values $p_{\text{Naive}}$ and ${\hat{p}}_{\text{selective}}\left({\hat{\sigma }}_{\text{MED}}\right)$ for all pairs of estimated clusters, and display results in the bottom panel of Table 1. Both sets of p-values are extremely small on this dataset, which suggests that the test based on our p-value has substantial power to reject the null hypothesis when it does not hold.

## 7. Discussion

We have proposed a test for a difference in means between two clusters estimated from k-means clustering, under (1). Methods developed in this paper are implemented in the R package Kmeans Inference, available at https://github.com/yiqunchen/Kmeans Inference. Data and code for reproducing the results in this paper can be found at https://github.com/yiqunchen/KmeansInference-experiments. Next, we outline a few directions for future research.

While the p-value in (9) leads to selective Type I error control, it conditions on more information than is used to construct the hypothesis in (2). In practice, data analysts likely only make use of the final cluster assignments (leading to the p-value in (8)), as opposed to all the intermediate assignments (leading to the p-value in (9)). Empirically, conditioning on too much information results in a loss of power (Fithian et al., 2014; Jewell et al., 2022; Liu et al., 2018). In future work, we will investigate the possibility of leveraging recent developments in selective inference (Chen et al., 2022; Le Duy and Takeuchi, 2021; Jewell et al., 2022) to compute the “ideal” p-value (8). Another line of future work is to extend our test for a pairwise difference in means to a difference among multiple groups (Kimes et al., 2017; Suzuki and Shimodaira, 2006). This might further provide a way to determine the number of clusters in k-means clustering.

We could also consider extending our proposal to other data generating models. The normality assumption in (1) is critical to the proof of Proposition 1, because it guarantees that under $H_{0}$ in (2), ${\left\|X^{⊤}\nu \right\|}_{2}$, $\text{dir}\left(X^{⊤}\nu \right)$, and $\Pi_{\nu }^{⊥}X$ are pairwise independent. However, this normality assumption is often violated in practice; for instance, in single-cell genomics, the data are count-valued and the variance of gene expression levels varies drastically with the mean expression levels of that gene (Stuart et al., 2019; Eling et al., 2018). This has motivated some authors to work with alternative models for gene expression including Poisson (Witten, 2011), negative binomial (Risso et al., 2018), and curved normal (Lin et al., 2021). To extend our framework to other exponential family distributions, we may be able to leverage recent proposals to decompose X into f(X) and g(X) such that both f(X) and g(X)∣f(X) have a known, computationally-tractable distribution (Rasines and Alastair Young, 2021; Leiner et al., 2021).

## Figures and Tables

**Figure 6:**
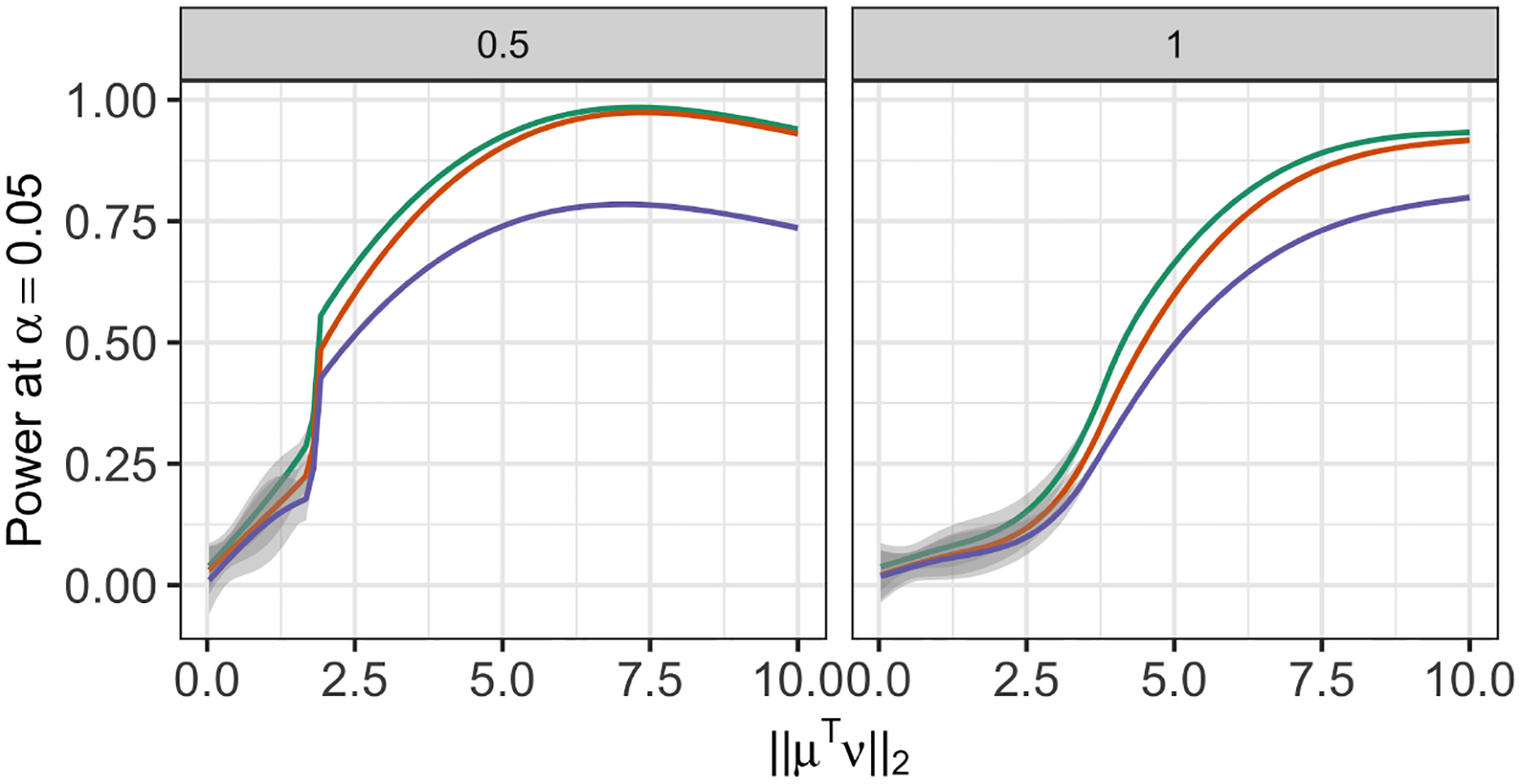
*Left* : Additional analysis of the data in Section 5.2 with σ=0.5. We fit a regression spline to display the power of the tests based on $p_{\text{selective}}$ (green line), ${\hat{p}}_{\text{selective}}\left({\hat{\sigma }}_{\text{MED}}\right)$ (orange line), and ${\hat{p}}_{\text{selective}}\left({\hat{\sigma }}_{\text{Sample}}\right)$ (purple line) as a function of ${\left\|\mu^{⊤}\nu \right\|}_{2}$. *Right*: Same as left, but for σ=1.

**Figure 7:**
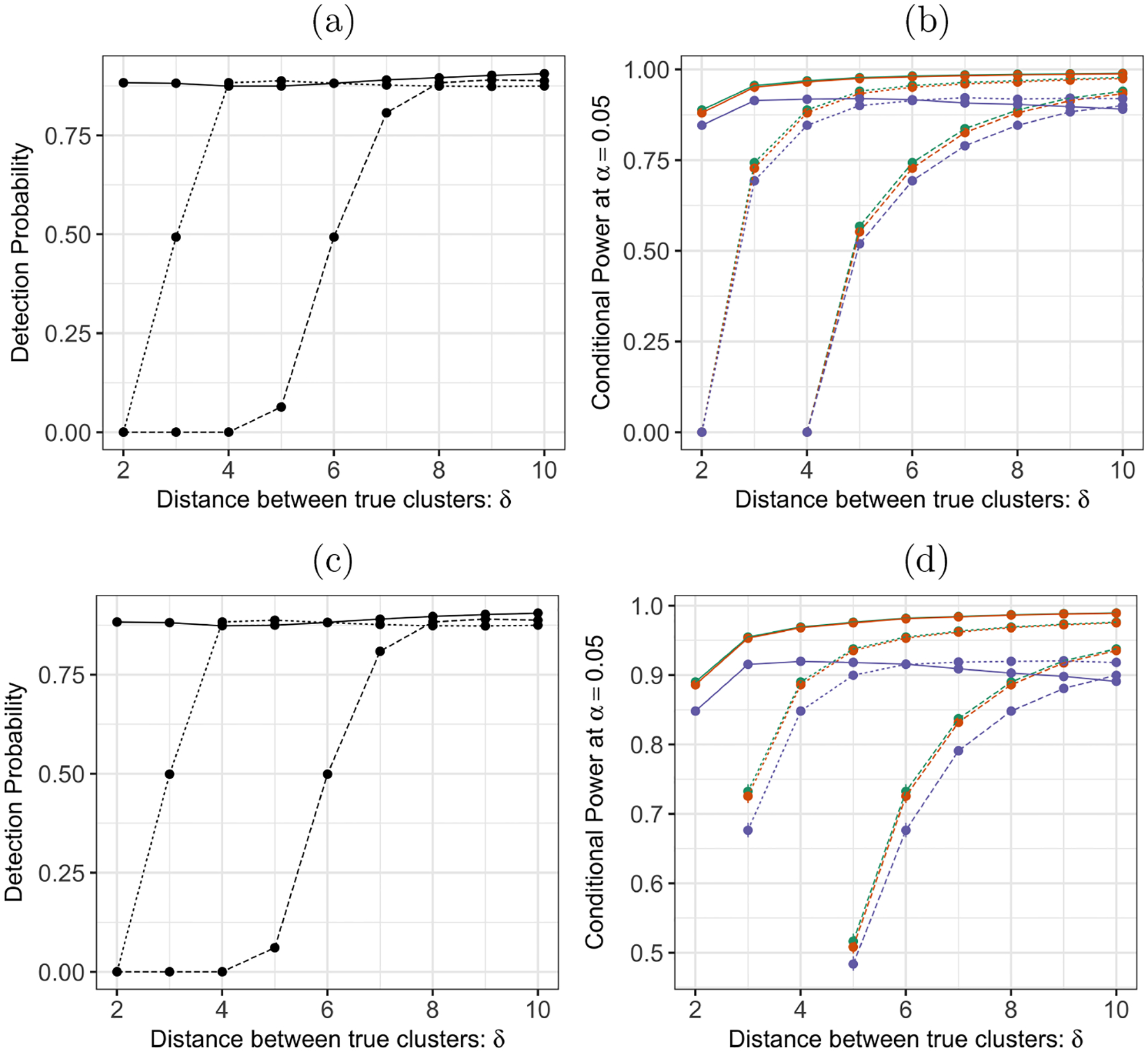
(*a*): Detection probability defined in (25) for k-means clustering with K=3 under model (1) with n=150, q=50, and μ in (A.54), across $\delta ={\left\|\theta_{i}-\theta_{j}\right\|}_{2}$ in (A.54) and σ=0.25 (solid lines), 0.5 (dashed lines), and 1 (long-dashed lines). (b): The conditional power (24) at α=0.05 for the tests based on $p_{\text{selective}}$ (green), ${\hat{p}}_{\text{selective}}\left({\hat{\sigma }}_{\text{MED}}\right)$ (orange), and ${\hat{p}}_{\text{selective}}\left({\hat{\sigma }}_{\text{Sample}}\right)$ (purple), under model (1) with n=150, q=50, and μ in (A.54). (c): Same as (a), but for μ in (23). (d): Same as (b), but for μ in (23).

**Table 1:** P-values $p_{\text{Naive}}$ in (4) and ${\hat{p}}_{\text{selective}}$ in (21) with ${\hat{\sigma }}_{\text{MED}}$ defined in (22) corresponding to the null hypothesis that the means of two estimated clusters are equal, for each pair of estimated clusters in the “no cluster” (top) and the “cluster” datasets (bottom).

$H_{0}$	${\bar{\mu }}_{1}={\bar{\mu }}_{2}$	${\bar{\mu }}_{1}={\bar{\mu }}_{3}$	${\bar{\mu }}_{1}={\bar{\mu }}_{4}$	${\bar{\mu }}_{1}={\bar{\mu }}_{5}$	${\bar{\mu }}_{2}={\bar{\mu }}_{3}$	${\bar{\mu }}_{2}={\bar{\mu }}_{4}$	${\bar{\mu }}_{2}={\bar{\mu }}_{5}$	${\bar{\mu }}_{3}={\bar{\mu }}_{4}$	${\bar{\mu }}_{3}={\bar{\mu }}_{5}$	${\bar{\mu }}_{4}={\bar{\mu }}_{5}$
$p_{\text{Naive}}$	< 10^−10^	< 10^−10^	< 10^−10^	< 10^−10^	< 10^−10^	< 10^−10^	< 10^−10^	< 10^−10^	< 10^−10^	< 10^−10^
${\hat{p}}_{\text{selective}}\left({\hat{\sigma }}_{\text{MED}}\right)$	0.30	0.310	0.43	0.12	0.12	0.:002	0.:10	0.005	0.04	0.05
$H_{0}$	${\bar{\mu }}_{1}={\bar{\mu }}_{2}$	${\bar{\mu }}_{1}={\bar{\mu }}_{3}$	${\bar{\mu }}_{1}={\bar{\mu }}_{4}$	${\bar{\mu }}_{1}={\bar{\mu }}_{5}$	${\bar{\mu }}_{2}={\bar{\mu }}_{3}$	${\bar{\mu }}_{2}={\bar{\mu }}_{4}$	${\bar{\mu }}_{2}={\bar{\mu }}_{5}$	${\bar{\mu }}_{3}={\bar{\mu }}_{4}$	${\bar{\mu }}_{3}={\bar{\mu }}_{5}$	${\bar{\mu }}_{4}={\bar{\mu }}_{5}$
$p_{\text{Naive}}$	< 10^−10^	< 10^−10^	< 10^−10^	< 10^−10^	< 10^−10^	< 10^−10^	< 10^−10^	< 10^−10^	< 10^−10^	< 10^−10^
${\hat{p}}_{\text{selective}}\left({\hat{\sigma }}_{\text{MED}}\right)$	4.0 × 10^−4^	< 10^−10^	< 10^−10^	< 10^−10^	< 10^−10^	< 10^−10^	< 10^−10^	< 10^−10^	5.0 × 10^−8^	< 10^−10^

## Appendix A. Appendix

### A.1 Proof of Proposition 1

The proof of Proposition 1 is similar to the proof of Theorem 1 in Gao et al. (2022), the proof of Theorem 3.1 in Loftus and Taylor (2015), the proof of Lemma 1 in Yang et al. (2016), and the proof of Theorem 3.1 in Chen and Bien (2020).

For any non-zero $\nu \in {\mathbb{R}}^{n}$ and $X\in {\mathbb{R}}^{n\times q}$, we have that

(A.26)
$$X=\Pi_{\nu }^{⊥}X+\left({\text{I}}_{n}-\Pi_{\nu }^{⊥}\right)X=\Pi_{\nu }^{⊥}X+\frac{\nu \nu^{⊤}X}{\|\nu {\|}_{2}^{2}}=\Pi_{\nu }^{⊥}X+\left(\frac{{\left\|X^{⊤}\nu \right\|}_{2}}{\|\nu {\|}_{2}^{2}}\right)\nu {\left\{\text{dir}\left(X^{⊤}\nu \right)\right\}}^{⊤}.$$

**Lemma 8** *Under* (1) *and* $H_{0}:\mu^{⊤}\nu =0_{q}$, *we have that* ${\left\|X^{⊤}\nu \right\|}_{2}$, $\Pi_{\nu }^{⊥}X$, *and* $\text{dir}\left(X^{⊤}\nu \right)$ *are pairwise independent*.

**Proof** We first prove that $X^{⊤}\nu$ is independent of $\Pi_{\nu }^{⊥}X$. The definition of $\Pi_{\nu }^{⊥}$ implies that $\Pi_{\nu }^{⊥}\nu =0_{n}$, and it follows from the properties of the matrix normal distribution that $\Pi_{\nu }^{⊥}X$ and $X^{⊤}\nu$ are independent. Therefore, ${\left\|X^{⊤}\nu \right\|}_{2}$ and $\text{dir}\left(X^{⊤}\nu \right)$ are independent of $\Pi_{\nu }^{⊥}X$ as well, since both are functions of $X^{⊤}\nu$.

Next, we will show that ${\left\|X^{⊤}\nu \right\|}_{2}$ and $\text{dir}\left(X^{⊤}\nu \right)$ are independent. Under (1) and $H_{0}:\mu^{⊤}\nu =0_{q}$, we have that $X^{⊤}\nu \sim 𝒩\left(0_{q},\sigma^{2}\|\nu {\|}_{2}^{2}I_{q}\right)$. It follows that $X^{⊤}\nu$ is rotationally invariant, and therefore ${\left\|X^{⊤}\nu \right\|}_{2}$ is independent of $\text{dir}\left(X^{⊤}\nu \right)$ (see, e.g., Proposition 4.1 and Corollary 4.3 of Bilodeau and Brenner (1999)). ■

We now proceed to prove the statement in (10). Recalling the definition of $p_{\text{selective}}$ in (9), under $H_{0}:\mu^{⊤}\nu =0_{q}$ with ν defined in (3), we have that

(A.27)
$$p_{\text{selective}}={\text{pr}}_{H_{0}}\left[{\left\|X^{⊤}\nu \right\|}_{2}\geq {\left\|x^{⊤}\nu \right\|}_{2}|\cap_{t=0}^{T}\cap_{i=1}^{n}\left\{c_{i}^{\left(t\right)}\left(X\right)=c_{i}^{\left(t\right)}\left(x\right)\right\},\Pi_{\nu }^{⊥}X=\Pi_{\nu }^{⊥}x,\text{ dir}\left(X^{⊤}\nu \right)=\text{dir}\left(x^{⊤}\nu \right)\right]\;\overset{a.}{=}{\text{pr}}_{H_{0}}\left[{\left\|X^{⊤}\nu \right\|}_{2}\geq {\left\|x^{⊤}\nu \right\|}_{2}|\overset{T}{\underset{t=0}{\cap }}\overset{n}{\underset{i=1}{\cap }}\left\{c_{i}^{\left(t\right)}\left(\Pi_{\nu }^{⊥}X+\left(\frac{{\left\|X^{⊤}\nu \right\|}_{2}}{\|\nu {\|}_{2}^{2}}\right)\nu {\left\{\text{dir}\left(X^{⊤}\nu \right)\right\}}^{⊤}\right)=c_{i}^{\left(t\right)}(x)\right\},\Pi_{\nu }^{⊥}X=\Pi_{\nu }^{⊥}x,\text{dir}\left(X^{⊤}\nu \right)=\text{dir}\left(x^{⊤}\nu \right)\right]\;\overset{b.}{=}{\text{pr}}_{H_{0}}\left[{\left\|X^{⊤}\nu \right\|}_{2}\geq {\left\|x^{⊤}\nu \right\|}_{2}|\overset{T}{\underset{t=0}{\cap }}\overset{n}{\underset{i=1}{\cap }}\left\{c_{i}^{\left(t\right)}\left(\Pi_{\nu }^{⊥}x+\left(\frac{{\left\|X^{⊤}\nu \right\|}_{2}}{\|\nu {\|}_{2}^{2}}\right)\nu {\left\{\text{dir}\left(x^{⊤}\nu \right)\right\}}^{⊤}\right)=c_{i}^{\left(t\right)}(x)\right\},\Pi_{\nu }^{⊥}X=\Pi_{\nu }^{⊥}x,\text{ dir}\left(X^{⊤}\nu \right)=\text{dir}\left(x^{⊤}\nu \right)\right]\;\overset{c.}{=}{\text{pr}}_{H_{0}}\left[{\left\|X^{⊤}\nu \right\|}_{2}\geq {\left\|x^{⊤}\nu \right\|}_{2}|\overset{T}{\underset{t=0}{\cap }}\overset{n}{\underset{i=1}{\cap }}\left\{c_{i}^{\left(t\right)}\left(\Pi_{\nu }^{⊥}x+\left(\frac{{\left\|X^{⊤}\nu \right\|}_{2}}{\|\nu {\|}_{2}^{2}}\right)\nu {\left\{\text{dir}\left(x^{⊤}\nu \right)\right\}}^{⊤}\right)=c_{i}^{\left(t\right)}(x)\right\}\right]\;\overset{d.}{=}{\text{pr}}_{H_{0}}\left[{\left\|X^{⊤}\nu \right\|}_{2}\geq {\left\|x^{⊤}\nu \right\|}_{2}|\overset{T}{\underset{t=0}{\cap }}\overset{n}{\underset{i=1}{\cap }}\left\{c_{i}^{\left(t\right)}\left(x^{\prime }\left({\left\|X^{⊤}\nu \right\|}_{2}\right)\right)=c_{i}^{\left(t\right)}(x)\right\}\right].$$

Here, step a. follows from substituting X with the expression in (A.26), and step b. follows from replacing $\Pi_{\nu }^{⊥}X$ and $\text{dir}\left(X^{⊤}\nu \right)$ with $\Pi_{\nu }^{⊥}x$ and $\text{dir}\left(x^{⊤}\nu \right)$, respectively. Next, in step c., we used Lemma 8. Finally, step d. follows from the definition of $x^{\prime }\left(\phi \right)$ in (11).

Note that under (1) and $H_{0}:\mu^{⊤}\nu =0_{q}$, we have that $X^{⊤}\nu_{2}\sim \sigma \|\nu {\|}_{2}\chi_{q}$, which concludes the proof of (10).

It remains to show that the test that rejects $H_{0}:\mu^{⊤}\nu =0$ when $p_{\text{selective}}\leq \alpha$ controls the selective Type I error at level α, in the sense of (5). First of all, recall that we decided to test the null hypothesis in (2) based on the output of Algorithm 1. Therefore, $p_{\text{selective}}$ controls the selective Type I error at level α if, for any $c_{i}^{\left(T\right)}\left(x\right),i=1,\;\ldots \;,n$,

(A.28)
$${\text{pr}}_{H_{0}}\left[\text{reject }H_{0}\text{ at level }\alpha |\overset{n}{\underset{i=1}{\cap }}\left\{c_{i}^{\left(T\right)}\left(X\right)=c_{i}^{\left(T\right)}\left(x\right)\right\}\right]\leq \alpha ,\;\forall \alpha \in \left(0,1\right).$$

To prove (A.28), we first note that the following holds for any α∈(0,1):

(A.29)
$${\text{pr}}_{H_{0}}\left[p_{\text{selective}}\left({\left\|X^{⊤}\nu \right\|}_{2}\right)\leq \alpha |\overset{T}{\underset{t=0}{\cap }}\overset{n}{\underset{i=1}{\cap }}\left\{c_{i}^{\left(t\right)}(X)=c_{i}^{\left(t\right)}(x)\right\},\Pi_{\nu }^{⊥}X=\Pi_{\nu }^{⊥}x,\text{ dir}\left(X^{⊤}\nu \right)=\text{dir}\left(x^{⊤}\nu \right)\right]\;\overset{a.}{=}{\text{pr}}_{H_{0}}\left[p_{\text{selective}}\left({\left\|X^{⊤}\nu \right\|}_{2}\right)\leq \alpha |\overset{T}{\underset{t=0}{\cap }}\overset{n}{\underset{i=1}{\cap }}\left\{c_{i}^{\left(t\right)}\left(\Pi_{\nu }^{⊥}X+\left(\frac{{\left\|X^{⊤}\nu \right\|}_{2}}{\|\nu {\|}_{2}^{2}}\right)\nu {\left\{\text{dir}\left(X^{⊤}\nu \right)\right\}}^{⊤}\right)=c_{i}^{\left(t\right)}(x)\right\},\Pi_{\nu }^{⊥}X=\Pi_{\nu }^{⊥}x,\text{ dir}\left(X^{⊤}\nu \right)=\text{dir}\left(x^{⊤}\nu \right)\right]\;\overset{b.}{=}{\text{pr}}_{H_{0}}\left[p_{\text{selective}}\left({\left\|X^{⊤}\nu \right\|}_{2}\right)\leq \alpha |\overset{T}{\underset{t=0}{\cap }}\overset{n}{\underset{i=1}{\cap }}\left\{c_{i}^{\left(t\right)}\left(\Pi_{\nu }^{⊥}x+\left(\frac{{\left\|X^{⊤}\nu \right\|}_{2}}{\|\nu {\|}_{2}^{2}}\right)\nu {\left\{\text{dir}\left(x^{⊤}\nu \right)\right\}}^{⊤}\right)=c_{i}^{\left(t\right)}(x)\right\},\Pi_{\nu }^{⊥}X=\Pi_{\nu }^{⊥}x,\text{ dir}\left(X^{⊤}\nu \right)=\text{dir}\left(x^{⊤}\nu \right)\right]\;\overset{c.}{=}{\text{pr}}_{H_{0}}\left[p_{\text{selective}}\left({\left\|X^{⊤}\nu \right\|}_{2}\right)\leq \alpha |\overset{T}{\underset{t=0}{\cap }}\overset{n}{\underset{i=1}{\cap }}\left\{c_{i}^{\left(t\right)}\left(\Pi_{\nu }^{⊥}x+\left(\frac{{\left\|X^{⊤}\nu \right\|}_{2}}{\|\nu {\|}_{2}^{2}}\right)\nu {\left\{\text{dir}\left(x^{⊤}\nu \right)\right\}}^{⊤}\right)=c_{i}^{\left(t\right)}(x)\right\}\right]\;\overset{d.}{=}{\text{pr}}_{H_{0}}\left[p_{\text{selective}}\left({\left\|X^{⊤}\nu \right\|}_{2}\right)\leq \alpha |\overset{T}{\underset{t=0}{\cap }}\overset{n}{\underset{i=1}{\cap }}\left\{c_{i}^{\left(t\right)}\left(x^{\prime }\left({\left\|X^{⊤}\nu \right\|}_{2}\right)\right)=c_{i}^{\left(t\right)}(x)\right\}\right]\;\overset{e.}{=}{\text{pr}}_{H_{0}}\left[1-F_{q}^{{𝒮}_{T}}\left({\left\|X^{⊤}\nu \right\|}_{2}\right)\leq \alpha |\overset{T}{\underset{t=0}{\cap }}\overset{n}{\underset{i=1}{\cap }}\left\{c_{i}^{\left(t\right)}\left(x^{\prime }\left({\left\|X^{⊤}\nu \right\|}_{2}\right)\right)=c_{i}^{\left(t\right)}(x)\right\}\right]\;\overset{f.}{=}\alpha .$$

Here, steps a. through d. follow from the same line of argument in (A.27). Moreover, (10) implies that, for a given sequence of cluster assignments $c_{i}^{\left(T\right)}\left(x\right)$, i=1, … ,n, $p_{\text{selective}}$ is the survival function of a $\chi_{q}$ random variable, truncated to the set ${𝒮}_{T}$ defined in (12). Letting $F_{q}^{{𝒮}_{T}}\left(\cdot \right)$ denote the cumulative distribution function of this truncated $\chi_{q}$ random variable, we arrive at step e. Finally, to prove f., we first note that under $H_{0}$, the conditional cumulative distribution function of ${\left\|X^{⊤}\nu \right\|}_{2}$ given $\cap_{t=0}^{T}\cap_{i=1}^{n}\left\{c_{i}^{\left(t\right)}\left(x^{\prime }\left({\left\|X^{⊤}\nu \right\|}_{2}\right)\right)=c_{i}^{\left(t\right)}\left(x\right)\right\}$ is exactly $F_{q}^{{𝒮}_{T}}$. The equality, therefore, follows from the probability integral transform, which states that for a continuous random variable Z, $F_{Z}\left(Z\right)$ follows the Uniform(0,1) distribution.

Finally, we have that

$${\text{pr}}_{H_{0}}\left[p_{\text{selective}}\left({\left\|X^{⊤}\nu \right\|}_{2}\right)\leq \alpha |\overset{n}{\underset{i=1}{\cap }}\left\{c_{i}^{\left(T\right)}(X)=c_{i}^{\left(T\right)}(x)\right\}\right]\;={\text{E}}_{H_{0}}\left[1\left\{p_{\text{selective}}\left({\left\|X^{⊤}\nu \right\|}_{2}\right)\leq \alpha \right\}|\overset{n}{\underset{i=1}{\cap }}\left\{c_{i}^{\left(T\right)}(X)=c_{i}^{\left(T\right)}(x)\right\}\right]\;\overset{a.}{=}{\text{E}}_{H_{0}}\left({\text{E}}_{H_{0}}\left[1\left\{p_{\text{selective}}\left({\left\|X^{⊤}\nu \right\|}_{2}\right)\leq \alpha \right\}\overset{T}{\underset{t=0}{\cap }}\overset{n}{\underset{i=1}{\cap }}\left\{c_{i}^{\left(t\right)}(X)=c_{i}^{\left(t\right)}(x)\right\},\Pi_{\nu }^{⊥}X=\Pi_{\nu }^{⊥}x,\text{ dir}\left(X^{⊤}\nu \right)=\text{dir}\left(x^{⊤}\nu \right)\right]|\overset{n}{\underset{i=1}{\cap }}\left\{c_{i}^{\left(T\right)}(X)=c_{i}^{\left(T\right)}(x)\right\}\right)\;\overset{b.}{=}{\text{E}}_{H_{0}}\left[\alpha |\overset{n}{\underset{i=1}{\cap }}\left\{c_{i}^{\left(T\right)}(X)=c_{i}^{\left(T\right)}(x)\right\}\right]\;=\alpha .$$

In the proof above, a. follows from the tower property of conditional expectation, and b. is a direct consequence of (A.29).

Therefore, we conclude that the test based on $p_{\text{selective}}$ controls the selective Type I error in (5), which completes the proof of Proposition 1.

### A.2 Proof of Proposition 2

We will derive the expression for ${𝒮}_{T}$ in Proposition 2 using an induction argument. For a positive integer K, we let [K] denote the set {1, … ,K}.

The following two claims (Lemmas 9 and 10) serve as the “base cases” for the proof.

**Lemma 9** *Recall that $c_{i}^{\left(t\right)}\left(x\right)$ denotes the cluster to which the ith observation is assigned during the tth iteration of step 3b*. *of* Algorithm 1 *applied to data* x, *and that* $m_{k}^{\left(0\right)}\left(x\right)$ *denotes the kth centroid sampled from* x *during step 1 of* Algorithm 1. *For* ${𝒮}_{0}$ *defined as*

(A.30)
$${𝒮}_{0}=\left\{\phi \in \mathbb{R}:\overset{n}{\underset{i=1}{\cap }}\left\{c_{i}^{\left(0\right)}\left(x^{\prime }(\phi )\right)=c_{i}^{\left(0\right)}(x)\right\}\right\},$$

we have that

(A.31)
$${𝒮}_{0}=\overset{n}{\underset{i=1}{\cap }}\overset{K}{\underset{k=1}{\cap }}\left\{\phi :{\left\|{\left[x^{\prime }(\phi )\right]}_{i}-m_{c_{i}^{\left(0\right)}(x)}^{\left(0\right)}\left(x^{\prime }(\phi )\right)\right\|}_{2}^{2}\leq {\left\|{\left[x^{\prime }(\phi )\right]}_{i}-m_{k}^{\left(0\right)}\left(x^{\prime }(\phi )\right)\right\|}_{2}^{2}\right\}.$$

**Proof** We first prove that the set in (A.30) is a subset of the set in (A.31). For an arbitrary $\phi_{0}\in (\text{A}.30)$ and 1≤i≤n, we have that

$$c_{i}^{\left(0\right)}\left(x^{\prime }\left(\phi_{0}\right)\right)=\underset{1\leq k\leq K}{\text{argmin}}{\left\|{\left[x^{\prime }\left(\phi_{0}\right)\right]}_{i}-m_{k}^{\left(0\right)}\left(x^{\prime }\left(\phi_{0}\right)\right)\right\|}_{2}^{2}\;\overset{a.}{\Rightarrow }{\left\|{\left[x^{\prime }\left(\phi_{0}\right)\right]}_{i}-m_{c_{i}^{\left(0\right)}\left(x^{\prime }\left(\phi_{0}\right)\right)}^{\left(0\right)}\left(x^{\prime }\left(\phi_{0}\right)\right)\right\|}_{2}^{2}\leq {\left\|{\left[x^{\prime }\left(\phi_{0}\right)\right]}_{i}-m_{k}^{\left(0\right)}\left(x^{\prime }\left(\phi_{0}\right)\right)\right\|}_{2}^{2},\forall k\in [K]\overset{b.}{\Rightarrow }{\left\|{\left[x^{\prime }\left(\phi_{0}\right)\right]}_{i}-m_{c_{i}^{\left(0\right)}(x)}^{\left(0\right)}\left(x^{\prime }\left(\phi_{0}\right)\right)\right\|}_{2}^{2}\leq {\left\|{\left[x^{\prime }\left(\phi_{0}\right)\right]}_{i}-m_{k}^{\left(0\right)}\left(x^{\prime }\left(\phi_{0}\right)\right)\right\|}_{2}^{2},\forall k\in [K].$$

Here, the first line follows from the definition of $c_{i}^{\left(0\right)}$ in step 2 of Algorithm 1, and step a. follows from the definition of the argmin function. Step b. follows from the assumption that $\phi_{0}\in \left(\text{A}.30\right)$ satisfies $c_{i}^{\left(0\right)}\left(x^{\prime }\left(\phi_{0}\right)\right)=c_{i}^{\left(0\right)}\left(x\right)$. Because this holds for an arbitrary 1≤i≤n, we have proven that $\phi_{0}\in (\text{A}.30)\Rightarrow \phi_{0}\in \left(\text{A}.31\right)$; or equivalently, (A.30) ⊆ (A.31).

We proceed to prove the other direction. For an arbitrary $\phi_{0}\in \left(\text{A}.31\right)$ and an arbitrary 1≤i≤n, we have that

$${\left\|{\left[x^{\prime }\left(\phi_{0}\right)\right]}_{i}-m_{c_{i}^{\left(0\right)}(x)}^{\left(0\right)}\left(x^{\prime }\left(\phi_{0}\right)\right)\right\|}_{2}^{2}\leq {\left\|{\left[x^{\prime }\left(\phi_{0}\right)\right]}_{i}-m_{k}^{\left(0\right)}\left(x^{\prime }\left(\phi_{0}\right)\right)\right\|}_{2}^{2},\forall k\in [K]\;\overset{a.}{\Rightarrow }c_{i}^{\left(0\right)}(x)=\underset{1\leq k\leq K}{\text{argmin}}{\left\|{\left[x^{\prime }\left(\phi_{0}\right)\right]}_{i}-m_{k}^{\left(0\right)}\left(x^{\prime }\left(\phi_{0}\right)\right)\right\|}_{2}^{2}\;\overset{b.}{\Rightarrow }c_{i}^{\left(0\right)}(x)=c_{i}^{\left(0\right)}\left(x^{\prime }\left(\phi_{0}\right)\right).$$

Here, step a. follows from the definition of argmin, and step b. follows from combining the definition of $c_{i}^{\left(0\right)}\left(x^{\prime }\left(\phi \right)\right)$ in step 2 of Algorithm 1. We conclude that $\phi_{0}\in \left(\text{A}.31\right)\Rightarrow \phi_{0}\in \left(\text{A}.30\right)$.

Combining these two directions, we have proven that (A.31) = (A.30). ■

**Lemma 10** *Recall that* $c_{i}^{\left(t\right)}\left(x\right)$ *denotes the cluster to which the ith observation is assigned in the* t*th iteration of step 3b*. *of* Algorithm 1 *applied to data* x, *and that* $m_{k}^{\left(0\right)}\left(x\right)$ *denotes the* k*th centroid sampled from* x *during step 1 of* Algorithm 1. *For* ${𝒮}_{1}$ *defined as*

(A.32)
$${𝒮}_{1}=\left\{\phi \in \mathbb{R}:\overset{1}{\underset{t=0}{\cap }}\overset{n}{\underset{i=1}{\cap }}\left\{c_{i}^{\left(t\right)}\left(x^{\prime }(\phi )\right)=c_{i}^{\left(t\right)}(x)\right\}\right\},$$

*and $w_{i}^{\left(t\right)}\left(k\right)$ defined in* (14), *we have that*

(A.33)
$${𝒮}_{1}=\overset{n}{\underset{i=1}{\cap }}\overset{K}{\underset{k=1}{\cap }}\left\{\phi :{\left\|{\left[x^{\prime }(\phi )\right]}_{i}-m_{c_{i}^{\left(0\right)}(x)}^{\left(0\right)}\left(x^{\prime }(\phi )\right)\right\|}_{2}^{2}\leq {\left\|{\left[x^{\prime }(\phi )\right]}_{i}-m_{k}^{\left(0\right)}\left(x^{\prime }(\phi )\right)\right\|}_{2}^{2}\right\}\cap \left(\overset{n}{\underset{i=1}{\cap }}\overset{K}{\underset{k=1}{\cap }}\left\{\phi :{\left\|{\left[x^{\prime }(\phi )\right]}_{i}-\sum_{i^{\prime }=1}^{n}w_{i^{\prime }}^{\left(0\right)}\left(c_{i}^{\left(1\right)}(x)\right){\left[x^{\prime }(\phi )\right]}_{i^{\prime }}\right\|}_{2}^{2}\leq {\left\|{\left[x^{\prime }(\phi )\right]}_{i}-\sum_{i^{\prime }=1}^{n}w_{i^{\prime }}^{\left(0\right)}(k){\left[x^{\prime }(\phi )\right]}_{i^{\prime }}\right\|}_{2}^{2}\right\}\right).$$

**Proof** We first prove that (A.32)⊆(A.33). For an arbitrary $\phi_{0}\in \left(\text{A}.32\right)$ and an arbitrary 1≤i≤n, we have that

$$c_{i}^{\left(1\right)}\left(x^{\prime }\left(\phi_{0}\right)\right)=\underset{1\leq k\leq K}{\text{argmin}}{\left\|{\left[x^{\prime }\left(\phi_{0}\right)\right]}_{i}-m_{k}^{\left(1\right)}\left(x^{\prime }\left(\phi_{0}\right)\right)\right\|}_{2}^{2}\;\overset{a.}{\Rightarrow }c_{i}^{\left(1\right)}(x)=\underset{1\leq k\leq K}{\text{argmin}}{\left\|{\left[x^{\prime }\left(\phi_{0}\right)\right]}_{i}-m_{k}^{\left(1\right)}\left(x^{\prime }\left(\phi_{0}\right)\right)\right\|}_{2}^{2}\;\overset{b.}{\Rightarrow }c_{i}^{\left(1\right)}(x)=\underset{1\leq k\leq K}{\text{argmin}}{\left\|{\left[x^{\prime }\left(\phi_{0}\right)\right]}_{i}-\frac{\sum_{i^{\prime }=1}^{n}1\left\{c_{i^{\prime }}^{\left(0\right)}\left(x^{\prime }\left(\phi_{0}\right)\right)=k\right\}{\left[x^{\prime }\left(\phi_{0}\right)\right]}_{i^{\prime }}}{\sum_{i^{\prime }=1}^{n}1\left\{c_{i^{\prime }}^{\left(0\right)}\left(x^{\prime }\left(\phi_{0}\right)\right)=k\right\}}\right\|}_{2}^{2}\;\overset{c.}{\Rightarrow }{\left\|{\left[x^{\prime }\left(\phi_{0}\right)\right]}_{i}-\frac{\sum_{i^{\prime }=1}^{n}1\left\{c_{z^{\prime }}^{\left(0\right)}\left(x^{\prime }\left(\phi_{0}\right)\right)=c_{i}^{\left(1\right)}(x)\right\}{\left[x^{\prime }\left(\phi_{0}\right)\right]}_{i^{\prime }}}{\sum_{i^{\prime }=1}^{n}1\left\{c_{i^{\prime }}^{\left(0\right)}\left(x^{\prime }\left(\phi_{0}\right)\right)=c_{i}^{\left(1\right)}(x)\right\}}\right\|}_{2}^{2}\leq {\left\|{\left[x^{\prime }\left(\phi_{0}\right)\right]}_{i}-\frac{\sum_{i^{\prime }=1}^{n}1\left\{c_{i^{\prime }}^{\left(0\right)}\left(x^{\prime }\left(\phi_{0}\right)\right)=k\right\}{\left[x^{\prime }\left(\phi_{0}\right)\right]}_{i^{\prime }}}{\sum_{i^{\prime }=1}^{n}1\left\{c_{i^{\prime }}^{\left(0\right)}\left(x^{\prime }\left(\phi_{0}\right)\right)=k\right\}}\right\|}_{2}^{2},\forall k\in [K]\;\overset{d.}{\Rightarrow }{\left\|{\left[x^{\prime }\left(\phi_{0}\right)\right]}_{i}-\frac{\sum_{i^{\prime }=1}^{n}1\left\{c_{i^{\prime }}^{\left(0\right)}(x)=c_{i}^{\left(1\right)}(x)\right\}{\left[x^{\prime }\left(\phi_{0}\right)\right]}_{i^{\prime }}}{\sum_{i^{\prime }=1}^{n}1\left\{c_{i^{\prime }}^{\left(0\right)}(x)=c_{i}^{\left(1\right)}(x)\right\}}\right\|}_{2}^{2}\leq {\left\|{\left[x^{\prime }\left(\phi_{0}\right)\right]}_{i}-\frac{\sum_{i^{\prime }=1}^{n}1\left\{c_{i^{\prime }}^{\left(0\right)}(x)=k\right\}{\left[x^{\prime }\left(\phi_{0}\right)\right]}_{i^{\prime }}}{\sum_{i^{\prime }=1}^{n}1\left\{c_{i^{\prime }}^{\left(0\right)}(x)=k\right\}}\right\|}_{2}^{2},\forall k\in [K]\;\overset{e.}{\Rightarrow }{\left\|{\left[x^{\prime }\left(\phi_{0}\right)\right]}_{i}-\sum_{i^{\prime }=1}^{n}w_{i^{\prime }}^{\left(0\right)}\left(c_{i}^{\left(1\right)}(x)\right){\left[x^{\prime }\left(\phi_{0}\right)\right]}_{i^{\prime }}\right\|}_{2}^{2}\leq {\left\|{\left[x^{\prime }\left(\phi_{0}\right)\right]}_{i}-\sum_{i^{\prime }=1}^{n}w_{i^{\prime }}^{\left(0\right)}(k){\left[x^{\prime }\left(\phi_{0}\right)\right]}_{i^{\prime }}\right\|}_{2}^{2},\forall k\in [K].$$

In the equations above, the first line follows from step 3b. of Algorithm 1 with t=0. Next, step a. follows from the definition of (A.32), which implies that $c_{i}^{\left(1\right)}\left(x^{\prime }\left(\phi_{0}\right)\right)=c_{i}^{\left(1\right)}\left(x\right)$. Step b. is a direct consequence of step 3a. of Algorithm 1 with t=0. In steps c. and d., we used the definitions of the argmin function and (A.32). Finally, we apply the definition of $w_{i}^{\left(t\right)}$ in (14) to get e. Because this holds for an arbitrary 1≤i≤n, $\phi_{0}\in \left(\text{A}.32\right)$ implies that $\phi_{0}$ is an element of the second set in the intersection in (A.33).

Moreover, $\phi_{0}\in \left(\text{A}.32\right)$ implies that $\phi_{0}\in \left(\text{A}.30\right)$, which, according to Lemma 9, further implies that $\phi_{0}$ is an element of the first set in the intersection in (A.33). To summarize, we have proven that $\phi_{0}\in \left(\text{A}.32\right)\Rightarrow \phi_{0}\in \left(\text{A}.33\right)$, and as a result, (A.32) ⊆ (A.33).

Next, we prove that the set in (A.33) is a subset of the set in (A.32). For an arbitrary $\phi_{0}\in \left(\text{A}.33\right)$ and an arbitrary 1≤i≤n, we have that

$$\phi_{0}\in (\text{A}.33)\overset{a.}{\Rightarrow }{\left\|{\left[x^{\prime }\left(\phi_{0}\right)\right]}_{i}-\frac{\sum_{i^{\prime }=1}^{n}1\left\{c_{i^{\prime }}^{\left(0\right)}(x)=c_{i}^{\left(1\right)}(x)\right\}{\left[x^{\prime }\left(\phi_{0}\right)\right]}_{i^{\prime }}}{\sum_{i^{\prime }=1}^{n}1\left\{c_{i^{\prime }}^{\left(0\right)}(x)=c_{i}^{\left(1\right)}(x)\right\}}\right\|}_{2}^{2}\leq {\left\|{\left[x^{\prime }\left(\phi_{0}\right)\right]}_{i}-\frac{\sum_{i^{\prime }=1}^{n}1\left\{c_{i^{\prime }}^{\left(0\right)}(x)=k\right\}{\left[x^{\prime }\left(\phi_{0}\right)\right]}_{i^{\prime }}}{\sum_{i^{\prime }=1}^{n}1\left\{c_{i^{\prime }}^{\left(0\right)}(x)=k\right\}}\right\|}_{2}^{2},\forall k\in [K]\;\overset{b.}{\Rightarrow }{\left\|{\left[x^{\prime }\left(\phi_{0}\right)\right]}_{i}-\frac{\sum_{i^{\prime }=1}^{n}1\left\{c_{i^{\prime }}^{\left(0\right)}\left(x^{\prime }\left(\phi_{0}\right)\right)=c_{i}^{\left(1\right)}(x)\right\}{\left[x^{\prime }\left(\phi_{0}\right)\right]}_{i^{\prime }}}{\sum_{i^{\prime }=1}^{n}1\left\{c_{i^{\prime }}^{\left(0\right)}\left(x^{\prime }\left(\phi_{0}\right)\right)=c_{i}^{\left(1\right)}(x)\right\}}\right\|}_{2}^{2}\leq {\left\|{\left[x^{\prime }\left(\phi_{0}\right)\right]}_{i}-\frac{\sum_{i^{\prime }=1}^{n}1\left\{c_{i^{\prime }}^{\left(0\right)}\left(x^{\prime }\left(\phi_{0}\right)\right)=k\right\}{\left[x^{\prime }\left(\phi_{0}\right)\right]}_{i^{\prime }}}{\sum_{i^{\prime }=1}^{n}1\left\{c_{i^{\prime }}^{\left(0\right)}\left(x^{\prime }\left(\phi_{0}\right)\right)=k\right\}}\right\|}_{2}^{2},\forall k\in [K]\;\overset{c.}{\Rightarrow }c_{i}^{\left(1\right)}(x)=\underset{1\leq k\leq K}{\text{argmin}}{\left\|{\left[x^{\prime }\left(\phi_{0}\right)\right]}_{i}-\frac{\sum_{i^{\prime }=1}^{n}1\left\{c_{i^{\prime }}^{\left(0\right)}\left(x^{\prime }\left(\phi_{0}\right)\right)=k\right\}{\left[x^{\prime }\left(\phi_{0}\right)\right]}_{i^{\prime }}}{\sum_{i^{\prime }=1}^{n}1\left\{c_{i^{\prime }}^{\left(0\right)}\left(x^{\prime }\left(\phi_{0}\right)\right)=k\right\}}\right\|}_{2}^{2}\;\overset{d.}{\Rightarrow }c_{i}^{\left(1\right)}(x)=\underset{1\leq k\leq K}{\text{argmin}}{\left\|{\left[x^{\prime }\left(\phi_{0}\right)\right]}_{i}-m_{k}^{\left(1\right)}\left(x^{\prime }\left(\phi_{0}\right)\right)\right\|}_{2}^{2}\;\overset{e.}{\Rightarrow }c_{i}^{\left(1\right)}(x)=c_{i}^{\left(1\right)}\left(x^{\prime }\left(\phi_{0}\right)\right).$$

Here, step a. follows from the definition of (A.33). In step b., we first apply Lemma 9, which implies that (A.33) ⊆ (A.31). Therefore, $\phi_{0}\in \left(\text{A}.33\right)\Rightarrow c_{i}^{\left(0\right)}\left(x\right)=c_{i}^{\left(0\right)}\left(x^{\prime }\left(\phi_{0}\right)\right)$, for all i=1, … ,n, k=1, … ,K, yielding the desired equality. Next, step c. follows from the definition of the argmin function. Finally, steps d. and e. follow directly from the definitions of $m_{k}^{\left(t\right)}$ and $c_{i}^{\left(t\right)}$ in steps 3a. and 3b. of Algorithm 1, respectively.

Because the result above holds for an arbitrary i, we have that $\phi_{0}\in \left(\text{A}.33\right)\Rightarrow c_{i}^{\left(1\right)}\left(x\right)=c_{i}^{\left(1\right)}\left(x^{\prime }\left(\phi \right)\right)$, i=1, … ,n. Combining this result with the observation that (A.33) ⊆ (A.31), we have that (A.33) ⊆ (A.32), which concludes the proof. ■

Next, we will prove the inductive step in the proof of Proposition 2, which relies on the following claim.

**Lemma 11** *Recall that* $c_{i}^{\left(t\right)}\left(x\right)$ *denotes the cluster to which the ith observation is assigned in the th iteration of* Algorithm 1 *applied to the data* x, *and that* $m_{k}^{\left(0\right)}\left(x\right)$ *denotes the kth centroid sampled from* x *during initialization*. *For some* $1\leq \tilde{T}\leq T-1$, *define*

(A.34)
$${𝒮}_{\tilde{T}}=\left\{\phi \in \mathbb{R}:\overset{\tilde{T}}{\underset{t=0}{\cap }}\overset{n}{\underset{i=1}{\cap }}\left\{c_{i}^{\left(t\right)}\left(x^{\prime }(\phi )\right)=c_{i}^{\left(t\right)}(x)\right\}\right\}.$$

*Suppose that the following holds for* $\tilde{T}$:

(A.35)
$${𝒮}_{\bar{T}}=\overset{n}{\underset{i=1}{\cap }}\overset{K}{\underset{k=1}{\cap }}\left\{\phi :{\left\|{\left[x^{\prime }(\phi )\right]}_{i}-m_{c_{i}^{\left(0\right)}(x)}^{\left(0\right)}\left(x^{\prime }(\phi )\right)\right\|}_{2}^{2}\leq {\left\|{\left[x^{\prime }(\phi )\right]}_{i}-m_{k}^{\left(0\right)}\left(x^{\prime }(\phi )\right)\right\|}_{2}^{2}\right\}\cap \left(\overset{\tilde{T}}{\underset{t=1}{\cap }}\overset{n}{\underset{i=1}{\cap }}\overset{K}{\underset{k=1}{\cap }}\left\{\phi :{\left\|{\left[x^{\prime }(\phi )\right]}_{i}-\sum_{i^{\prime }=1}^{n}w_{i^{\prime }}^{\left(t-1\right)}\left(c_{i}^{\left(t\right)}(x)\right){\left[x^{\prime }(\phi )\right]}_{i^{\prime }}\right\|}_{2}^{2}\leq {\left\|{\left[x^{\prime }(\phi )\right]}_{i}-\sum_{i^{\prime }=1}^{n}w_{i^{\prime }}^{\left(t-1\right)}(k){\left[x^{\prime }(\phi )\right]}_{i^{\prime }}\right\|}_{2}^{2}\right\}\right),$$

*where* $w_{i}^{\left(t\right)}\left(\cdot \right)$ *is defined in* (14). *Then*, *for* ${𝒮}_{\tilde{T}+1}$ *defined as*

(A.36)
$${𝒮}_{\tilde{T}+1}=\left\{\phi \in \mathbb{R}:\overset{\tilde{T}+1}{\underset{t=0}{\cap }}\overset{n}{\underset{i=1}{\cap }}\left\{c_{i}^{\left(t\right)}\left(x^{\prime }(\phi )\right)=c_{i}^{\left(t\right)}(x)\right\}\right\},$$

we have that

(A.37)
$${𝒮}_{\bar{T}+1}=\overset{n}{\underset{i=1}{\cap }}\overset{K}{\underset{k=1}{\cap }}\left\{\phi :{\left\|{\left[x^{\prime }(\phi )\right]}_{i}-m_{c_{i}^{\left(0\right)}(x)}^{\left(0\right)}\left(x^{\prime }(\phi )\right)\right\|}_{2}^{2}\leq {\left\|{\left[x^{\prime }(\phi )\right]}_{i}-m_{k}^{\left(0\right)}\left(x^{\prime }(\phi )\right)\right\|}_{2}^{2}\right\}\cap \left(\overset{\tilde{T}+1}{\underset{t=1}{\cap }}\overset{n}{\underset{i=1}{\cap }}\overset{K}{\underset{k=1}{\cap }}\left\{\phi :{\left\|{\left[x^{\prime }(\phi )\right]}_{i}-\sum_{i^{\prime }=1}^{n}w_{i^{\prime }}^{\left(t-1\right)}\left(c_{i}^{\left(t\right)}(x)\right){\left[x^{\prime }(\phi )\right]}_{i^{\prime }}\right\|}_{2}^{2}\leq {\left\|{\left[x^{\prime }(\phi )\right]}_{i}-\sum_{i^{\prime }=1}^{n}w_{i^{\prime }}^{\left(t-1\right)}(k){\left[x^{\prime }(\phi )\right]}_{i^{\prime }}\right\|}_{2}^{2}\right\}\right).$$

**Proof** Using the definitions in (A.34) and (A.36), we have that

(A.38)
$${𝒮}_{\tilde{T}+1}={𝒮}_{\tilde{T}}\cap \left(\overset{n}{\underset{i=1}{\cap }}\left\{\phi \in \mathbb{R}:c_{i}^{\left(\tilde{T}+1\right)}\left(x^{\prime }(\phi )\right)=c_{i}^{\left(\tilde{T}+1\right)}(x)\right\}\right).$$

Therefore, it suffices to prove that (A.38) = (A.37), under the inductive hypothesis (A.35).

We start by proving that (A.38) ⊆ (A.37). For an arbitrary $\phi_{0}\in \left(\text{A}.38\right)$ and an arbitrary 1≤i≤n, we have that

$$c_{i}^{\left(\tilde{T}+1\right)}\left(x^{\prime }\left(\phi_{0}\right)\right)=c_{i}^{\left(\bar{T}+1\right)}(x)\overset{a.}{\Rightarrow }c_{i}^{\left(\tilde{T}+1\right)}(x)=\underset{1\leq k\leq K}{\text{argmin}}{\left\|{\left[x^{\prime }\left(\phi_{0}\right)\right]}_{i}-m_{k}^{\left(\tilde{T}+1\right)}\left(x^{\prime }\left(\phi_{0}\right)\right)\right\|}_{2}^{2}\;\overset{b.}{\Rightarrow }{\left\|{\left[x^{\prime }\left(\phi_{0}\right)\right]}_{i}-\frac{\sum_{i^{\prime }=1}^{n}1\left\{c_{i^{\prime }}^{\left(\bar{T}\right)}\left(x^{\prime }\left(\phi_{0}\right)\right)=c_{i}^{\left(\tilde{T}+1\right)}(x)\right\}{\left[x^{\prime }\left(\phi_{0}\right)\right]}_{i^{\prime }}}{\sum_{i^{\prime }=1}^{n}1\left\{c_{i^{\prime }}^{\left(\bar{T}\right)}\left(x^{\prime }\left(\phi_{0}\right)\right)=c_{i}^{\left(\tilde{T}+1\right)}(x)\right\}}\right\|}_{2}^{2}\leq {\left\|{\left[x^{\prime }\left(\phi_{0}\right)\right]}_{i}-\frac{\sum_{i^{\prime }=1}^{n}1\left\{c_{i^{\prime }}^{\left(\bar{T}\right)}\left(x^{\prime }\left(\phi_{0}\right)\right)=k\right\}{\left[x^{\prime }\left(\phi_{0}\right)\right]}_{i^{\prime }}}{\sum_{i^{\prime }=1}^{n}1\left\{c_{i^{\prime }}^{\left(\bar{T}\right)}\left(x^{\prime }\left(\phi_{0}\right)\right)=k\right\}}\right\|}_{2}^{2},\forall k\in [K]\;\overset{c.}{\Rightarrow }{\left\|{\left[x^{\prime }\left(\phi_{0}\right)\right]}_{i}-\frac{\sum_{i^{\prime }=1}^{n}1\left\{c_{i^{\prime }}^{\left(\bar{T}\right)}(x)=c_{i}^{\left(\bar{T}+1\right)}(x)\right\}{\left[x^{\prime }\left(\phi_{0}\right)\right]}_{i^{\prime }}}{\sum_{i^{\prime }=1}^{n}1\left\{c_{i^{\prime }}^{\left(\bar{T}\right)}(x)=c_{i}^{\left(\bar{T}+1\right)}(x)\right\}}\right\|}_{2}^{2}\leq {\left\|{\left[x^{\prime }\left(\phi_{0}\right)\right]}_{i}-\frac{\sum_{i^{\prime }=1}^{n}1\left\{c_{i^{\prime }}^{\left(\bar{T}\right)}(x)=k\right\}{\left[x^{\prime }\left(\phi_{0}\right)\right]}_{i^{\prime }}}{\sum_{i^{\prime }=1}^{n}1\left\{c_{i^{\prime }}^{\left(\tilde{T}\right)}(x)=k\right\}}\right\|}_{2}^{2},\forall k\in [K]\;\overset{d.}{\Rightarrow }\phi_{0}\in (\text{A}.37).$$

Here, the first statement follows from the definition of ${𝒮}_{\tilde{T}+1}$. Next, steps a. and b. follow from the definitions of $c_{i}^{\left(\tilde{T}+1\right)}$ and $m_{k}^{\left(\tilde{T}+1\right)}\left(x^{\prime }\left(\phi_{0}\right)\right)$ in steps 3b. and 3a. of Algorithm 1, respectively. In step c., we used the fact that $\phi_{0}\in \left(\text{A}.38\right)\Rightarrow \phi_{0}\in {𝒮}_{\tilde{T}}\Rightarrow c_{i}^{\tilde{T}}\left(x^{\prime }\left(\phi_{0}\right)\right)=c_{i}^{\tilde{T}}\left(x\right)$. Finally, d. follows from the definition of $w_{i}^{\left(t\right)}$ in (14).

We continue with the reverse direction. Applying the inductive hypothesis (A.35), together with the definition of $S_{\tilde{T}+1}$ in (A.37) and the definition of $w_{i}^{\left(t\right)}$ in (14), we have that

(A.39)
$$(\text{A}.37)=S_{\tilde{T}}\cap \left(\overset{n}{\underset{i=1}{\cap }}\overset{K}{\underset{k=1}{\cap }}\left\{\phi :{\left\|{\left[x^{\prime }(\phi )\right]}_{i}-\frac{\sum_{i^{\prime }=1}^{n}1\left\{c_{i^{\prime }}^{\left(\bar{T}\right)}(x)=c_{i}^{\left(\tilde{T}+1\right)}(x)\right\}{\left[x^{\prime }(\phi )\right]}_{i^{\prime }}}{\sum_{i^{\prime }=1}^{n}1\left\{c_{i^{\prime }}^{\left(\tilde{T}\right)}(x)=c_{i}^{\left(\tilde{T}+1\right)}(x)\right\}}\right\|}_{2}^{2}\leq {\left\|{\left[x^{\prime }(\phi )\right]}_{i}-\frac{\sum_{i^{\prime }=1}^{n}1\left\{c_{i^{\prime }}^{\left(\tilde{T}\right)}(x)=k\right\}{\left[x^{\prime }(\phi )\right]}_{i^{\prime }}}{\sum_{i^{\prime }=1}^{n}1\left\{c_{i^{\prime }}^{\left((\tilde{T}\right)}(x)=k\right\}}\right\|}_{2}^{2}\right\}\right).$$

For an arbitrary $\phi_{0}\in \left(\text{A}.37\right)$ and any 1≤i≤n, the following holds:

$${\left\|{\left[x^{\prime }\left(\phi_{0}\right)\right]}_{i}-\frac{\sum_{i^{\prime }=1}^{n}1\left\{c_{i^{\prime }}^{\left(\tilde{T}\right)}(x)=c_{i}^{\left(\tilde{T}+1\right)}(x)\right\}{\left[x^{\prime }\left(\phi_{0}\right)\right]}_{i^{\prime }}}{\sum_{i^{\prime }=1}^{n}1\left\{c_{i^{\prime }}^{\left(\tilde{T}\right)}(x)=c_{i}^{\left(\tilde{T}+1\right)}(x)\right\}}\right\|}_{2}^{2}\leq {\left\|{\left[x^{\prime }\left(\phi_{0}\right)\right]}_{i}-\frac{\sum_{i^{\prime }=1}^{n}1\left\{c_{i^{\prime }}^{\left(\tilde{T}\right)}(x)=k\right\}{\left[x^{\prime }\left(\phi_{0}\right)\right]}_{i^{\prime }}}{\sum_{i^{\prime }=1}^{n}1\left\{c_{i^{\prime }}^{\left(\tilde{T}\right)}(x)=k\right\}}\right\|}_{2}^{2},\forall k\in [K]\;\overset{a.}{\Rightarrow }{\left\|{\left[x^{\prime }\left(\phi_{0}\right)\right]}_{i}-\frac{\sum_{i^{\prime }=1}^{n}1\left\{c_{i^{\prime }}^{\left(\tilde{T}\right)}\left(x^{\prime }\left(\phi_{0}\right)\right)=c_{i}^{\left(\tilde{T}+1\right)}(x)\right\}{\left[x^{\prime }\left(\phi_{0}\right)\right]}_{i^{\prime }}}{\sum_{i^{\prime }=1}^{n}1\left\{c_{i^{\prime }}^{\left(\tilde{T}\right)}\left(x^{\prime }\left(\phi_{0}\right)\right)=c_{i}^{\left(\tilde{T}+1\right)}(x)\right\}}\right\|}_{2}^{2}\leq {\left\|{\left[x^{\prime }\left(\phi_{0}\right)\right]}_{i}-\frac{\sum_{i^{\prime }=1}^{n}1\left\{c_{i^{\prime }}^{\left(\tilde{T}\right)}\left(x^{\prime }\left(\phi_{0}\right)\right)=k\right\}{\left[x^{\prime }\left(\phi_{0}\right)\right]}_{i^{\prime }}}{\sum_{i^{\prime }=1}^{n}1\left\{c_{i^{\prime }}^{\left(\tilde{T}\right)}\left(x^{\prime }\left(\phi_{0}\right)\right)=k\right\}}\right\|}_{2}^{2},\forall k\in [K]\;\overset{b.}{\Rightarrow }c_{i}^{\left(\tilde{T}+1\right)}(x)=\underset{1\leq k\leq K}{\text{argmin}}{\left\|{\left[x^{\prime }\left(\phi_{0}\right)\right]}_{i}-m_{k}^{\left(\tilde{T}+1\right)}\left(x^{\prime }\left(\phi_{0}\right)\right)\right\|}_{2}^{2}\;\overset{c.}{\Rightarrow }c_{i}^{\left(\tilde{T}+1\right)}(x)=c_{i}^{\left(\tilde{T}+1\right)}\left(x^{\prime }\left(\phi_{0}\right)\right).$$

Here, to derive step a., we first note that by (A.39), any element $\phi_{0}$ of (A.37) is also an element of ${𝒮}_{\tilde{T}}$. Therefore, using the definition of ${𝒮}_{\tilde{T}}$ in (A.34), we have that $\cap_{t=1}^{\tilde{T}}\left\{c_{i}^{\left(t\right)}\left(x^{\prime }\left(\phi_{0}\right)\right)=c_{i}^{\left(t\right)}\left(x\right)\right\}$, and step a. follows directly. Next, steps b. and c. follow directly from steps 3a. and 3b. of Algorithm 1 with $t=\tilde{T}$. By inspecting the form of (A.38), we conclude that $\phi_{0}\in \left(\text{A}.37\right)\Rightarrow \phi_{0}\in \left(\text{A}.38\right)$.

In conclusion, we have proven that (A.37) = (A.38), which completes the proof. ■

The inductive proof of Proposition 2 follows from combining Lemmas 9, 10 and 11.

### A.3 Proof of Lemmas 3 and 4

We first prove Lemma 3, which is also Lemma 2 in Gao et al. (2022).

**Proof** We first express the inner product $\langle {\left[x^{\prime }\left(\phi \right)\right]}_{i},{\left[x^{\prime }\left(\phi \right)\right]}_{j}\rangle$ as a function of ϕ. From (11), we have that ${\left[x^{\prime }\left(\phi \right)\right]}_{i}=x_{i}+\nu_{i}\left(\frac{\phi -{\left\|x^{⊤}\nu \right\|}_{2}}{\|\nu {\|}_{2}^{2}}\right)\text{dir}\left(x^{⊤}\nu \right)=x_{i}-\nu_{i}\frac{{\left\|x^{⊤}\nu \right\|}_{2}}{\|\nu {\|}_{2}^{2}}\text{dir}\left(x^{⊤}\nu \right)+\left(\frac{\nu_{i}}{\|\nu {\|}_{2}^{2}}\phi \right)\text{dir}\left(x^{⊤}\nu \right)$.

Therefore,

$$\langle {\left[x^{\prime }(\phi )\right]}_{i},{\left[x^{\prime }(\phi )\right]}_{j}\rangle =\langle x_{i}-\nu_{i}\frac{{\left\|x^{⊤}\nu \right\|}_{2}}{\|\nu {\|}_{2}^{2}}\text{dir}\left(x^{⊤}\nu \right)+\left(\frac{\nu_{i}}{\|\nu {\|}_{2}^{2}}\phi \right)\text{dir}\left(x^{⊤}\nu \right),x_{j}-\nu_{j}\frac{{\left\|x^{⊤}\nu \right\|}_{2}}{\|\nu {\|}_{2}^{2}}\text{dir}\left(x^{⊤}\nu \right)+\left(\frac{\nu_{j}}{\|\nu {\|}_{2}^{2}}\phi \right)\text{dir}\left(x^{⊤}\nu \right)\rangle \;={\left(\frac{{\left(\nu_{i}\nu_{j}\right)}^{1/2}}{\|\nu {\|}_{2}^{2}}\phi \right)}^{2}+\langle x_{i}-\nu_{i}\frac{{\left\|x^{⊤}\nu \right\|}_{2}}{\|\nu {\|}_{2}^{2}}\text{dir}\left(x^{⊤}\nu \right),\left(\frac{\nu_{j}}{\|\nu {\|}_{2}^{2}}\right)\text{dir}\left(x^{⊤}\nu \right)\rangle \cdot \phi +\langle x_{j}-\nu_{j}\frac{{\left\|x^{⊤}\nu \right\|}_{2}}{\|\nu {\|}_{2}^{2}}\text{dir}\left(x^{⊤}\nu \right),\left(\frac{\nu_{i}}{\|\nu {\|}_{2}^{2}}\right)\text{dir}\left(x^{⊤}\nu \right)\rangle \cdot \phi +\langle x_{i}-\nu_{i}\frac{{\left\|x^{⊤}\nu \right\|}_{2}}{\|\nu {\|}_{2}^{2}}\text{dir}\left(x^{⊤}\nu \right),x_{j}-\nu_{j}\frac{{\left\|x^{⊤}\nu \right\|}_{2}}{\|\nu {\|}_{2}^{2}}\text{dir}\left(x^{⊤}\nu \right)\rangle \;={\left(\frac{{\left(\nu_{i}\nu_{j}\right)}^{1/2}}{\|\nu {\|}_{2}^{2}}\right)}^{2}\phi^{2}+\left(\frac{\nu_{j}}{\|\nu {\|}_{2}^{2}}\langle x_{i},\text{dir}\left(x^{⊤}\nu \right)\rangle +\frac{\nu_{i}}{\|\nu {\|}_{2}^{2}}\langle x_{j},\text{dir}\left(x^{⊤}\nu \right)\rangle -2\frac{\nu_{i}\nu_{j}{\left\|x^{⊤}\nu \right\|}_{2}}{\|\nu {\|}_{2}^{4}}\right)\phi +\langle x_{i}-\nu_{i}\frac{{\left\|x^{⊤}\nu \right\|}_{2}}{\|\nu {\|}_{2}^{2}}\text{dir}\left(x^{⊤}\nu \right),x_{j}-\nu_{j}\frac{{\left\|x^{⊤}\nu \right\|}_{2}}{\|\nu {\|}_{2}^{2}}\text{dir}\left(x^{⊤}\nu \right)\rangle .$$

Next, using the expression for $\langle {\left[x^{\prime }\left(\phi \right)\right]}_{i},{\left[x^{\prime }\left(\phi \right)\right]}_{j}\rangle$ above, we have that

$${\left\|{\left[x^{\prime }(\phi )\right]}_{i}-{\left[x^{\prime }(\phi )\right]}_{j}\right\|}_{2}^{2}=\langle {\left[x^{\prime }(\phi )\right]}_{i}-{\left[x^{\prime }(\phi )\right]}_{j},{\left[x^{\prime }(\phi )\right]}_{i}-{\left[x^{\prime }(\phi )\right]}_{j}\rangle \;=\langle x_{i}-x_{j}-\left(\nu_{i}-\nu_{j}\right)\frac{{\left\|x^{⊤}\nu \right\|}_{2}}{\|\nu {\|}_{2}^{2}}\text{dir}\left(x^{⊤}\nu \right)+\left(\frac{\left(\nu_{i}-\nu_{j}\right)}{\|\nu {\|}_{2}^{2}}\phi \right)\text{dir}\left(x^{⊤}\nu \right),x_{i}-x_{j}-\left(\nu_{i}-\nu_{j}\right)\frac{{\left\|x^{⊤}\nu \right\|}_{2}}{\|\nu {\|}_{2}^{2}}\text{dir}\left(x^{⊤}\nu \right)+\left(\frac{\left(\nu_{i}-\nu_{j}\right)}{\|\nu {\|}_{2}^{2}}\phi \right)\text{dir}\left(x^{⊤}\nu \right)\rangle \;={\left(\frac{\nu_{i}-\nu_{j}}{\|\nu {\|}_{2}^{2}}\right)}^{2}\phi^{2}+2\left(\frac{\nu_{i}-\nu_{j}}{\|\nu {\|}_{2}^{2}}\langle x_{i}-x_{j},\text{dir}\left(x^{⊤}\nu \right)\rangle -{\left(\frac{\nu_{i}-\nu_{j}}{\|\nu {\|}_{2}^{2}}\right)}^{2}{\left\|x^{⊤}\nu \right\|}_{2}\right)\phi +{\left\|x_{i}-x_{j}-\left(\nu_{i}-\nu_{j}\right)\frac{x^{⊤}\nu }{\|\nu {\|}_{2}^{2}}\right\|}_{2}^{2}.$$

■ This completes the proof of Lemma 3.

We continue with the proof of Lemma 4. Using the definition of $w_{i}^{\left(t-1\right)}\left(k\right)$ in (14), we have that

$${\left\|{\left[x^{\prime }(\phi )\right]}_{i}-\frac{\sum_{i^{\prime }=1}^{n}1\left\{c_{i^{\prime }}^{\left(t-1\right)}(x)=k\right\}{\left[x^{\prime }(\phi )\right]}_{i^{\prime }}}{\sum_{i^{\prime }=1}^{n}1\left\{c_{i^{\prime }}^{\left(t-1\right)}(x)=k\right\}}\right\|}_{2}^{2}={\left\|{\left[x^{\prime }(\phi )\right]}_{i}-\sum_{i^{\prime }=1}^{n}w_{i^{\prime }}^{\left(t-1\right)}(k){\left[x^{\prime }(\phi )\right]}_{i^{\prime }}\right\|}_{2}^{2},$$

where

$${\left[x^{\prime }(\phi )\right]}_{i}-\sum_{i^{\prime }=1}^{n}w_{i^{\prime }}^{\left(t-1\right)}(k){\left[x^{\prime }(\phi )\right]}_{i^{\prime }}=\left(\sum_{i^{\prime }=1}^{n}w_{i^{\prime }}^{\left(t-1\right)}(k)\frac{\nu_{i}}{\|\nu {\|}_{2}^{2}}\right)\phi +\sum_{i^{\prime }=1}^{n}w_{i^{\prime }}^{\left(t-1\right)}(k)\left(x_{i}-\nu_{i}\frac{{\left\|x^{⊤}\nu \right\|}_{2}}{\|\nu {\|}_{2}^{2}}\text{dir}\left(x^{⊤}\nu \right)\right)$$

is a linear function of ϕ. The rest of the proof follows directly from the same set of calculations in the proof of Lemma 3.

### A.4 Proof of Proposition 5

Recall that n, q, K, T denote the number of samples (see (1)), the number of features (see (1)), the number of clusters (see Algorithm 1), and the maximum number of iterations for which Algorithm 1 is run.

According to Proposition 2, to compute the set ${𝒮}_{T}$ in (12), it suffices to compute the intersection of the two sets in (15) and (16).

We first make the following observations for our timing complexity analysis:

- Observation 1: according to Lemma 3, the set in (15) is an intersection of nK quadratic inequalities.
- Observation 2: according to Lemma 4, the set in (16) is an intersection of nKT quadratic inequalities.
- Observation 3: we can solve a quadratic inequality in 𝒪(1) time using the quadratic formula.
- Observation 4: we can intersect the solution sets of N quadratic inequalities in 𝒪(N log N) time (Bourgon, 2020).

Equipped with these observations, we will analyze the timing complexity of computing the set (15). Note that the coefficients for each of the nK quadratic inequalities can be computed in 𝒪(nq) operations: first, using the property that $x^{⊤}\nu =\sum_{i\in {\hat{𝒞}}_{1}}x_{i}/\left|{\hat{𝒞}}_{1}\right|-\sum_{i\in {\hat{𝒞}}_{2}}x_{i}/\left|{\hat{𝒞}}_{2}\right|$, we can compute $x^{⊤}\nu_{2}$ and $\text{dir}\left(x^{⊤}\nu \right)$ in 𝒪(nq) operations. Then, computing the coefficients a, b, and γ in Lemma 3 takes 𝒪(1), 𝒪(q), and 𝒪(q) operations, respectively. For each inequality, obtaining the solution set requires 𝒪(1) operations (see Observation 3). Finally, intersecting the solution sets of the n(K−1) quadratic inequalities incurs another 𝒪(nK log(nK)) operations. Thus, the computational cost for (15) totals to 𝒪(nKq+nK log(nK)) operations.

Next, we analyze the cost of computing the set (16). Note that using Observation 2, we need to solve nKT quadratic inequalities. Here, for each quadratic inequality of the form in Lemma 4, it takes 𝒪(n), 𝒪(n+q), and 𝒪(n+q) operations to compute the coefficients $\tilde{a}$, $\tilde{b}$, and $\tilde{\gamma }$, respectively. Moreover, for a given iteration t and cluster number k, we only need to compute $\sum_{i^{\prime }=1}^{n}w_{i^{\prime }}^{\left(t-1\right)}\left(k\right)\nu_{i^{\prime }}$ once using 𝒪(n+q) operations once, as opposed to n times, since this formula does not depend on the index i. Therefore, obtaining the nKT solution sets will take 𝒪((n+q)KT) time. Finally, intersecting these sets using Observation 4 adds another 𝒪(nKT log(nKT)) operations.

Combining the costs for computing the set in (15) and the set in (16), we conclude that the cost for computing the set ${𝒮}_{T}$ in (12) is 𝒪((n+q)KT+nKT log(nKT)) operations.

### A.5 Proof of Proposition 6 and computation of $p_{\Sigma ,\text{selective}}$

The proof of Proposition 6 is similar to that of Proposition 1.

First note that for any non-zero $\nu \in {\mathbb{R}}^{n}$ and $X\in {\mathbb{R}}^{n\times q}$, we have that

(A.40)
$$X=\Pi_{\nu }^{⊥}X+\frac{\nu \nu^{⊤}X\Sigma^{-\frac{1}{2}}\Sigma^{\frac{1}{2}}}{\|\nu {\|}_{2}^{2}}=\Pi_{\nu }^{⊥}X+\left(\frac{{\left\|\Sigma^{-\frac{1}{2}}X^{⊤}\nu \right\|}_{2}}{\|\nu {\|}_{2}^{2}}\right)\nu {\left\{\text{dir}\left(\Sigma^{-\frac{1}{2}}X^{⊤}\nu \right)\right\}}^{⊤}\Sigma^{\frac{1}{2}}.$$

**Lemma 12** *Under* (17) *and* $H_{0}:\mu^{⊤}\nu =0_{q}$, ${\left\|\Sigma^{-\frac{1}{2}}X^{⊤}\nu \right\|}_{2}$, $\Pi_{\nu }^{⊥}X$ *and* $\text{dir}\left(\Sigma^{-\frac{1}{2}}X^{⊤}\nu \right)$ *are pairwise independent*.

**Proof** As in the proof of Lemma 8, $\Pi_{\nu }^{⊥}\nu =0_{n}$, and it follows from the property of the matrix normal distribution that $X^{⊤}\nu$ is independent of $\Pi_{\nu }^{⊥}X$. Because both ${\left\|\Sigma^{-\frac{1}{2}}X^{⊤}\nu \right\|}_{2}$ and $\text{dir}\left(\Sigma^{-\frac{1}{2}}X^{⊤}\nu \right)$ are functions of $X^{⊤}\nu$, both are independent of $\Pi_{\nu }^{⊥}X$.

Next, we will show that ${\left\|\Sigma^{-\frac{1}{2}}X^{⊤}\nu \right\|}_{2}$ and $\text{dir}\left(\Sigma^{-\frac{1}{2}}X^{⊤}\nu \right)$ are independent. Under (17) and $H_{0}:\mu^{⊤}\nu =0_{q}$, we have that $\Sigma^{-\frac{1}{2}}X^{⊤}\nu \sim 𝒩\left(0_{q},\|\nu {\|}_{2}^{2}I_{q}\right)$. It then follows that $\Sigma^{-\frac{1}{2}}X^{⊤}\nu$ is rotationally invariant, and therefore ${\left\|\Sigma^{-\frac{1}{2}}X^{⊤}\nu \right\|}_{2}$ is independent of $\text{dir}\left(\Sigma^{-\frac{1}{2}}X^{⊤}\nu \right)$ (Bilodeau and Brenner, 1999). ■

Then, recalling the definition of $p_{\Sigma ,\text{selective}}$ in (19), we have that

$$p_{\Sigma \text{,selective}}={\text{pr}}_{H_{0}}\left[{\left\|\Sigma^{-\frac{1}{2}}X^{⊤}\nu \right\|}_{2}\geq {\left\|\Sigma^{-\frac{1}{2}}x^{⊤}\nu \right\|}_{2}|\overset{T}{\underset{t=0}{\cap }}\overset{n}{\underset{i=1}{\cap }}\left\{c_{i}^{\left(t\right)}(X)=c_{i}^{\left(t\right)}(x)\right\},\Pi_{\nu }^{⊥}X=\Pi_{\nu }^{⊥}x,\text{ dir}\left(\Sigma^{-\frac{1}{2}}X^{⊤}\nu \right)=\text{dir}\left(\Sigma^{-\frac{1}{2}}x^{⊤}\nu \right)\right]\;\overset{a.}{=}{\text{pr}}_{H_{0}}\left[{\left\|\Sigma^{-\frac{1}{2}}X^{⊤}\nu \right\|}_{2}\geq {\left\|\Sigma^{-\frac{1}{2}}x^{⊤}\nu \right\|}_{2}|\overset{T}{\underset{t=0}{\cap }}\overset{n}{\underset{i=1}{\cap }}\left\{c_{i}^{\left(t\right)}\left(\Pi_{\nu }^{⊥}X+\frac{{\left\|\Sigma^{-\frac{1}{2}}X^{⊤}\nu \right\|}_{2}}{\|\nu {\|}_{2}^{2}}\nu {\left\{\text{dir}\left(\Sigma^{-\frac{1}{2}}X^{⊤}\nu \right)\right\}}^{⊤}\Sigma^{\frac{1}{2}}\right)=c_{i}^{\left(t\right)}(x)\right\},\Pi_{\nu }^{⊥}X=\Pi_{\nu }^{⊥}x,\text{dir}\left(\Sigma^{-\frac{1}{2}}X^{⊤}\nu \right)=\text{dir}\left(\Sigma^{-\frac{1}{2}}x^{⊤}\nu \right)\right]\;\overset{b.}{=}{\text{pr}}_{H_{0}}\left[{\left\|\Sigma^{-\frac{1}{2}}X^{⊤}\nu \right\|}_{2}\geq {\left\|\Sigma^{-\frac{1}{2}}x^{⊤}\nu \right\|}_{2}|\overset{T}{\underset{t=0}{\cap }}\overset{n}{\underset{i=1}{\cap }}\left\{c_{i}^{\left(t\right)}\left(\Pi_{\nu }^{⊥}x+\frac{{\left\|\Sigma^{-\frac{1}{2}}X^{⊤}\nu \right\|}_{2}}{\|\nu {\|}_{2}^{2}}\nu {\left\{\text{dir}\left(\Sigma^{-\frac{1}{2}}x^{⊤}\nu \right)\right\}}^{⊤}\Sigma^{\frac{1}{2}}\right)=c_{i}^{\left(t\right)}(x)\right\},\Pi_{\nu }^{⊥}X=\Pi_{\nu }^{⊥}x,\text{dir}\left(\Sigma^{-\frac{1}{2}}X^{⊤}\nu \right)=\text{dir}\left(\Sigma^{-\frac{1}{2}}x^{⊤}\nu \right)\right]\overset{c.}{=}{\text{pr}}_{H_{0}}\left[{\left\|\Sigma^{-\frac{1}{2}}X^{⊤}\nu \right\|}_{2}\geq {\left\|\Sigma^{-\frac{1}{2}}x^{⊤}\nu \right\|}_{2}|\overset{T}{\underset{t=0}{\cap }}\overset{n}{\underset{i=1}{\cap }}\left\{c_{i}^{\left(t\right)}\left(\Pi_{\nu }^{⊥}x+\frac{{\left\|\Sigma^{-\frac{1}{2}}X^{⊤}\nu \right\|}_{2}}{\|\nu {\|}_{2}^{2}}\nu {\left\{\text{dir}\left(\Sigma^{-\frac{1}{2}}x^{⊤}\nu \right)\right\}}^{⊤}\Sigma^{\frac{1}{2}}\right)=c_{i}^{\left(t\right)}(x)\right\}\right].$$

Here, step a. follows from substituting X with the expression in (A.40). Step b. follows from replacing $\Pi_{\nu }^{⊥}X$ and $\text{dir}\left(\Sigma^{-\frac{1}{2}}X^{⊤}\nu \right)$ with $\Pi_{\nu }^{⊥}x$ and $\text{dir}\left(\Sigma^{-\frac{1}{2}}x^{⊤}\nu \right)$, respectively. Finally, in step c., we used Lemma 12. Now, under (17) and $H_{0}:\mu^{⊤}\nu =0_{q}$, we have that ${\left\|\Sigma^{-\frac{1}{2}}X^{⊤}\nu \right\|}_{2}\sim \|\nu {\|}_{2}\chi_{q}$, which concludes the proof of (20).

It remains to show that the test that rejects $H_{0}:\mu^{⊤}\nu =0$ when $p_{\Sigma ,\text{selective}}\leq \alpha$ controls the selective Type I error, in the sense of (5). We omit the proof here, as it follows directly from the proof of Proposition 1 in Appendix A.1.

Next, we discuss how we could modify the results in Section 3 to compute the p-value $p_{\Sigma ,\text{selective}}$. First note that according to Proposition 6, it suffices to compute the set

(A.41)
$${𝒮}_{T}^{\Sigma }=\left\{\phi :\overset{T}{\underset{t=0}{\cap }}\overset{n}{\underset{i=1}{\cap }}\left\{c_{i}^{\left(t\right)}\left(\Pi_{\nu }^{⊥}x+\left(\frac{\phi }{\|\nu {\|}_{2}^{2}}\right)\nu {\left\{\text{dir}\left(\Sigma^{-\frac{1}{2}}x^{⊤}\nu \right)\right\}}^{⊤}\Sigma^{\frac{1}{2}}\right)=c_{i}^{\left(t\right)}(x)\right\}\right\}.$$

In addition, letting $\tilde{x^{\prime }}\left(\phi \right)$ denote $\Pi_{\nu }^{⊥}x+\left(\frac{\phi }{\|\nu {\|}_{2}^{2}}\right)\nu {\left\{\text{dir}\left(\Sigma^{-\frac{1}{2}}x^{⊤}\nu \right)\right\}}^{⊤}\Sigma^{\frac{1}{2}}$, we see that $\tilde{x^{\prime }}\left(\phi \right)$ is in fact a linear function of ϕ with

(A.42)
$${\left[\tilde{x^{\prime }}\left(\phi \right)\right]}_{i}=x_{i}-\nu_{i}\frac{\left\|x^{⊤}\nu_{2}\right\|}{\|\nu {\|}_{2}^{2}}\text{dir}\left(x^{⊤}\nu \right)+\left(\frac{\left\|x^{⊤}\nu_{2}\right\|}{{\left\|\Sigma^{-\frac{1}{2}}x^{⊤}\nu \right\|}_{2}}\frac{\nu_{i}}{\|\nu {\|}_{2}^{2}}\phi \right)\text{dir}\left(x^{⊤}\nu \right).$$

Therefore, a minor modification of Proposition 2 yields the following corollary.

**Corollary 13** *Suppose the* k-*means clustering algorithm (see* Algorithm 1) *with* K *clusters the data* x, *when applied to the data* x, *runs for* T *steps*. *Then*, *for the set* ${𝒮}_{T}^{\Sigma }$ *defined in* (A.41), *we have that*

(A.43)
$${𝒮}_{T}^{\Sigma }=\left(\overset{n}{\underset{i=1}{\cap }}\overset{K}{\underset{k=1}{\cap }}\left\{\phi :{\left\|{\left[{\tilde{x}}^{\prime }(\phi )\right]}_{i}-m_{c_{i}^{\left(0\right)}(x)}^{\left(0\right)}(\phi )\right\|}_{2}^{2}\leq {\left\|{\left[{\tilde{x}}^{\prime }(\phi )\right]}_{i}-m_{k}^{\left(0\right)}(\phi )\right\|}_{2}^{2}\right\}\right)\cap \left(\overset{T}{\underset{t=1}{\cap }}\overset{n}{\underset{i=1}{\cap }}\overset{K}{\underset{k=1}{\cap }}\left\{\phi :{\left\|{\left[{\tilde{x}}^{\prime }(\phi )\right]}_{i}-\sum_{i^{\prime }=1}^{n}w_{i^{\prime }}^{\left(t-1\right)}\left(c_{i^{\prime }}^{\left(t-1\right)}(x)\right){\left[{\tilde{x}}^{\prime }(\phi )\right]}_{i^{\prime }}\right\|}_{2}^{2}\leq {\left\|{\left[{\tilde{x}}^{\prime }(\phi )\right]}_{i}-\sum_{i^{\prime }=1}^{n}w_{i^{\prime }}^{\left(t-1\right)}(k){\left[{\tilde{x}}^{\prime }(\phi )\right]}_{i^{\prime }}\right\|}_{2}^{2}\right\}\right).$$

We also have the following extensions of Lemmas 3 and 4, which enable efficient computation of the expressions in Corollary 13.

**Lemma 14 (Section 4.2 in** Gao et al. (2022)) *For* $\tilde{x^{\prime }}\left(\phi \right)$ *in* (A.42) *and* ν *in* (3), ${\left\|{\left[\tilde{x^{\prime }}\left(\phi \right)\right]}_{i}-{\left[\tilde{x^{\prime }}\left(\phi \right)\right]}_{j}\right\|}_{2}^{2}=a^{\prime }\phi^{2}+b^{\prime }\phi +\gamma^{\prime }$, *where* $a^{\prime }={\left(\frac{{\left\|x^{⊤}\nu \right\|}_{2}}{{\left\|\Sigma^{-\frac{1}{2}}x^{⊤}\nu \right\|}_{2}}\right)}^{2}{\left(\frac{\nu_{i}-\nu_{j}}{\|\nu {\|}_{2}^{2}}\right)}^{2}$, $b^{\prime }=2\left(\frac{{\left\|x^{⊤}\nu \right\|}_{2}}{{\left\|\Sigma^{-\frac{1}{2}}x^{⊤}\nu \right\|}_{2}}\right)\left(\frac{\nu_{i}-\nu_{j}}{\|\nu {\|}_{2}^{2}}x_{i}-x_{j},\text{dir}\left(x^{⊤}\nu \right)-{\left(\frac{\nu_{i}-\nu_{j}}{\|\nu {\|}_{2}^{2}}\right)}^{2}{\left\|x^{⊤}\nu \right\|}_{2}\right)$. *and* $\gamma^{\prime }={\left\|x_{i}-x_{j}-\left(\nu_{i}-\nu_{j}\right)\frac{x^{⊤}\nu }{\|\nu {\|}_{2}^{2}}\right\|}_{2}^{2}$.

**Lemma 15** *For* $\tilde{x^{\prime }}\left(\phi \right)$ *in* (A.42), ν *in* (3), *and* $w_{i}^{\left(t\right)}\left(k\right)$ *in* (14), ${\left\|{\left[\tilde{x^{\prime }}\left(\phi \right)\right]}_{i}-\sum_{i^{\prime }=1}^{n}w_{i^{\prime }}^{\left(t-1\right)}\left(k\right){\left[\tilde{x^{\prime }}\left(\phi \right)\right]}_{i^{\prime }}\right\|}_{2}^{2}=\tilde{a^{\prime }}\phi^{2}+\tilde{b^{\prime }}\phi +{\tilde{\gamma }}^{\prime }$, *where*

$${\tilde{a}}^{\prime }=\frac{1}{\|\nu {\|}_{2}^{4}}{\left(\frac{{\left\|x^{⊤}\nu \right\|}_{2}}{{\left\|\Sigma^{-\frac{1}{2}}x^{⊤}\nu \right\|}_{2}}\right)}^{2}{\left(\nu_{i}-\sum_{i^{\prime }=1}^{n}w_{i^{\prime }}^{\left(t-1\right)}(k)\nu_{i^{\prime }}\right)}^{2},{\tilde{b}}^{\prime }=\left(\frac{2{\left\|x^{⊤}\nu \right\|}_{2}}{\|\nu {\|}_{2}^{2}{\left\|\Sigma^{-\frac{1}{2}}x^{⊤}\nu \right\|}_{2}}\right)\left\{\left(\nu_{i}-\sum_{i^{\prime }=1}^{n}w_{i^{\prime }}^{\left(t-1\right)}(k)\nu_{i^{\prime }}\right)\langle x_{i}-\sum_{i^{\prime }=1}^{n}w_{i^{\prime }}^{\left(t-1\right)}(k)x_{i^{\prime }},\text{dir}\left(x^{⊤}\nu \right)\rangle -\frac{{\left\|x^{⊤}\nu \right\|}_{2}}{\|\nu {\|}_{2}^{4}}{\left(\nu_{i}-\sum_{i^{\prime }=1}^{n}w_{i^{\prime }}^{\left(t-1\right)}(k)\nu_{i^{\prime }}\right)}^{2}\right\},$$

and

$${\tilde{\gamma }}^{\prime }={\left\|x_{i}-\sum_{i^{\prime }=1}^{n}w_{i^{\prime }}^{\left(t-1\right)}(k)x_{i^{\prime }}-\left(\nu_{i}-\sum_{i^{\prime }=1}^{n}w_{i^{\prime }}^{\left(t-1\right)}(k)\nu_{i^{\prime }}\right)\frac{x^{⊤}\nu }{\|\nu {\|}_{2}^{2}}\right\|}_{2}^{2}.$$

Proofs of Lemmas 14 and 15 follow from the same set of calculations in the proofs of Lemmas 3 and 4 in Appendix A.3.

### A.6 Proof of Proposition 7

Proof of Proposition 7 is similar to the proof of Lemma 1 in Markovic et al. (2017) and the proof of Lemma 7 in Tibshirani et al. (2018).

We first present an auxiliary lemma.

**Lemma 16** *For any* $c_{i}^{\left(t\right)}\left(x\right)$, i=1, … ,n; t=1, … ,T, ${\hat{p}}_{\text{selective}}\left(\hat{\sigma }\right)$ *defined in* (21) *is a continuous and monotonically increasing function of* $\hat{\sigma }$.

**Proof** By the definition in (21), we have that

(A.44)
$${\hat{p}}_{\text{selective}}(\hat{\sigma })=\frac{\int_{{\left\|x^{⊤}\nu \right\|}_{2}}^{\infty }{\left(\frac{1}{2}\right)}^{q/2-1}\frac{t^{q-1}}{\Gamma (q/2)}\|\nu {\|}_{2}^{-q}{\hat{\sigma }}^{-q}\text{ exp}\left(-\frac{t^{2}}{2{\hat{\sigma }}^{2}\|\nu {\|}_{2}^{2}}\right)1\left\{t\in {𝒮}_{T}\right\}dt}{\int_{0}^{\infty }{\left(\frac{1}{2}\right)}^{q/2-1}\frac{t^{q-1}}{\Gamma (q/2)}\|\nu {\|}_{2}^{-q}{\hat{\sigma }}^{-q}\text{ exp}\left(-\frac{t^{2}}{2{\hat{\sigma }}^{2}\|\nu {\|}_{2}^{2}}\right)1\left\{t\in {𝒮}_{T}\right\}dt},$$

where ${𝒮}_{T}$ defined in (12) is a function of $c_{i}^{\left(t\right)}\left(x\right)$, i=1, … ,n; t=1, … ,T. By inspection, (A.44) is a continuous function of $\hat{\sigma }$, because the product or ratio of two continuous functions is still continuous. It remains to show that (A.44) is increasing in $\hat{\sigma }$. This follows directly from Lemma S3. of Gao et al. (2022).

Provided that $\hat{\sigma }$ converges to σ in probability, we can combine Lemma 16 and the continuous mapping theorem to see that ${\hat{p}}_{\text{selective}}\left(\hat{\sigma }\right)$ converges to $p_{\text{selective}}\left(\sigma \right)$ in probability, i.e., for all ϵ>0, ${\text{lim}}_{q\to \infty }\text{pr}\left(\left|{\hat{p}}_{\text{selective}}\left(\hat{\sigma }\right)-p_{\text{selective}}\left(\sigma \right)\right|\geq \epsilon \right)=0$. Next, letting $A_{q}$ denote the event $\cap_{t=0}^{T}\cap_{i=1}^{n}\left\{c_{i}^{\left(t\right)}\left(X^{\left(q\right)}\right)=c_{i}^{\left(t\right)}\left(x^{\left(q\right)}\right)\right\}$, we will show that under the assumptions in Proposition 7, ${\hat{p}}_{\text{selective}}\left(\hat{\sigma }\right)$ converges to $p_{\text{selective}}\left(\sigma \right)$ in probability, *conditional on* $A_{q}$. For any ϵ>0, we have that

$$\underset{q\to \infty }{\text{lim}}{\text{pr}}_{H_{0}^{\left(q\right)}}\left\{\left|{\hat{p}}_{\text{selective}}(\hat{\sigma })-p_{\text{selective}}(\sigma )\right|\leq \epsilon |A_{q}\right\}\;\overset{a.}{=}\underset{q\to \infty }{\text{lim}}\frac{{\text{pr}}_{H_{0}^{\left(q\right)}}\left\{\left|{\hat{p}}_{\text{selective}}(\hat{\sigma })-p_{\text{selective}}(\sigma )\right|\leq \epsilon ,A_{q}\right\}}{{\text{pr}}_{H_{0}^{\left(q\right)}}\left(A_{q}\right)}\;\overset{b.}{\geq }\underset{q\to \infty }{\text{lim}}\frac{{\text{pr}}_{H_{0}^{\left(q\right)}}\left(A_{q}\right)-{\text{pr}}_{H_{0}^{\left(q\right)}}\left\{\left|{\hat{p}}_{\text{selective}}(\hat{\sigma })-p_{\text{selective}}(\sigma )\right|>\epsilon \right\}}{{\text{pr}}_{H_{0}^{\left(q\right)}}\left(A_{q}\right)}\;\overset{c.}{=}\frac{{\text{lim}}_{q\to \infty }\;{\text{pr}}_{H_{0}^{\left(q\right)}}\left(A_{q}\right)-{\text{lim}}_{q\to \infty }\;{\text{pr}}_{H_{0}^{\left(q\right)}}\left\{\left|{\hat{p}}_{\text{selective}}(\hat{\sigma })-p_{\text{selective}}(\sigma )\right|>\epsilon \right\}}{{\text{lim}}_{q\to \infty }\;{\text{pr}}_{H_{0}^{\left(q\right)}}\left(A_{q}\right)}\;\overset{d.}{=}\frac{\delta }{\delta }=1.$$

Here, step a. follows from Bayes rule, and the observation that the denominator is non-zero for finite q. In step b., we used the lower bound that for events A,B defined on the same probability space, $\text{pr}\left(A\cap B\right)=\text{pr}\left(A\right)-\text{pr}\left(A\setminus B\right)\geq \text{pr}\left(A\right)-\text{pr}\left(B^{C}\right)$. Next, c. follows from distributing the limit, which is valid because of the assumption that ${\text{lim}}_{q\to \infty }{\text{pr}}_{H_{0}^{\left(q\right)}}\left(A_{q}\right)=\delta >0$; finally, d. follows from the fact that ${\hat{p}}_{\text{selective}}\left(\hat{\sigma }\right)$ converges to $p_{\text{selective}}\left(\sigma \right)$ in probability for any sequence of $\mu^{\left(q\right)}$, q=1,2,…, which implies the convergence under $H_{0}:\mu^{{\left(q\right)}^{⊤}}\nu^{\left(q\right)}=0$ as well.

Finally, we have that

(A.45)
$$\underset{q\to \infty }{\text{lim}}{\text{pr}}_{H_{0}^{\left(q\right)}}\left\{{\hat{p}}_{\text{selective}}\left(\hat{\sigma }\right)\leq \alpha |A_{q}\right\}\overset{a.}{=}\underset{q\to \infty }{\text{lim}}{\text{pr}}_{H_{0}^{\left(q\right)}}\left\{p_{\text{selective}}\left(\sigma \right)\leq \alpha |A_{q}\right\}\overset{b.}{=}\underset{q\to \infty }{\text{lim}}\alpha =\alpha .$$

Here, step a. follows from ${\hat{p}}_{\text{selective}}\left(\hat{\sigma }\right)$ converging to $p_{\text{selective}}\left(\sigma \right)$ in probability, conditional on $A_{q}$. Step b. follows from the fact that the result of Proposition 1 applies for any positive integer q. This completes the proof of Proposition 7.

Proposition 7 assumes that we have a consistent estimator $\hat{\sigma }$ of σ. In Appendix A.7, we analyze different estimators of σ in (1), and prove that, under appropriate sparsity assumptions on μ in (1), ${\hat{\sigma }}_{\text{MED}}$ in (22) is a consistent estimator for σ.

As an alternative, we can also use an asymptotically conservative estimator of σ as in Gao et al. (2022). This leads to an asymptotically conservative p-value; details are stated in Corollary 17.

**Corollary 17** *For* q=1,2,…, *suppose that* $X^{\left(q\right)}\sim ℳ{𝒩}_{n\times q}\left(\mu^{\left(q\right)},I_{n},\sigma^{2}I_{q}\right)$. *Let* $x^{\left(q\right)}$ *be a realization from* $X^{\left(q\right)}$ *and* $c_{i}^{\left(t\right)}\left(\cdot \right)$ *be the cluster to which the ith observation is assigned during the th iteration of step 3b*. *of* Algorithm 1. *Consider the sequence of null hypotheses* $H_{0}^{\left(q\right)}:\mu^{{\left(q\right)}^{⊤}}\nu^{\left(q\right)}=0_{q}$, *where* $\nu^{\left(q\right)}$ *defined in* (3) *is the contrast vector resulting from applying* k-*means clustering on* $x^{\left(q\right)}$. *Suppose that (i)* $\hat{\sigma }$ *is an asymptotically conservative estimator of* σ, *i*.*e*., ${\text{lim}}_{q\to \infty }\text{pr}\left(\hat{\sigma }\left(X^{\left(q\right)}\right)\geq \sigma \right)=1$; *and* (ii) *there exists δ∈(0,1) such that* ${\text{lim}}_{q\to \infty }{\text{pr}}_{H_{0}^{\left(q\right)}}\left[\cap_{t=0}^{T}\cap_{i=1}^{n}\left\{c_{i}^{\left(t\right)}\left(X^{\left(q\right)}\right)=c_{i}^{\left(t\right)}\left(x^{\left(q\right)}\right)\right\}\right]>\delta$. *Then*, ∀α∈(0,1), *we have that* ${\text{lim}}_{q\to \infty }{\text{pr}}_{H_{0}^{\left(q\right)}}\left[{\hat{p}}_{\text{selective}}\left(\hat{\sigma }\right)\leq \alpha |\cap_{t=0}^{T}\cap_{i=1}^{n}\left\{c_{i}^{\left(t\right)}\left(X^{\left(q\right)}\right)=c_{i}^{\left(t\right)}\left(x^{\left(q\right)}\right)\right\}\right]\leq \alpha$.

We omit the proof of Corollary 17, as it follows directly from combining the proof of Proposition 7 and the fact that ${\hat{p}}_{\text{selective}}\left(\hat{\sigma }\right)$ is a monotonically increasing function of $\hat{\sigma }$ (see Lemma 16).

Finally, we remark that, in principle, the result in Proposition 7 can be extended to an unknown covariance matrix Σ. However, estimating Σ is challenging, especially when q is comparable to, or larger than, n (Rousseeuw, 1987; Bickel and Levina, 2008; Avella-Medina et al., 2018). It may be possible to leverage recent advances in robust covariance matrix estimation (e.g., Han and Liu (2014); Chen et al. (2018); Belomestny et al. (2019)) to obtain a consistent estimator of Σ under model (17).

### A.7 Estimating σ in (1)

Proposition 7 states that, under appropriate assumptions, a consistent estimator of σ in (1) leads to asymptotic selective Type I error control. In this section, we analyze the asymptotic behavior of the two variance estimators considered in Section 5, ${\hat{\sigma }}_{\text{MED}}^{2}$ and ${\hat{\sigma }}_{\text{Sample}}^{2}$. In particular, we prove that under model (1) and a sparsity assumption on μ (defined in (1)), a close analog of ${\hat{\sigma }}_{\text{MED}}^{2}$ in (22) that does not subtract the column median is a consistent estimator of $\sigma^{2}$. Moreover, we prove that ${\hat{\sigma }}_{\text{Sample}}^{2}$ is a conservative estimator of $\sigma^{2}$, and characterize its exact bias.

We first introduce an auxiliary result that specifies the rate of convergence for a median-based estimator of the variance in the sparse vector model (Comminges et al., 2021). For a vector $\theta \in {\mathbb{R}}^{n}$, we use $\|\theta {\|}_{0}$ to denote its $\ell_{0}$ norm, i.e. $\|\theta {\|}_{0}=\sum_{i=1}^{n}1\left\{\theta_{i}\neq 0\right\}$.

**Lemma 18 (Proposition 6 in** Comminges et al. (2021)) *Consider the model*

(A.46)
$$Y_{i}=\theta_{i}+\sigma \xi_{i},\;i=1,\;\ldots \;,d,$$

*where* σ *is unknown*, *and the independently and identically distributed noise* $\xi_{i}$ *satisfies that* (i) $\text{E}\left(\xi_{i}\right)=0$; (ii) $\text{E}\left(\xi_{i}^{2}\right)=1$; *and* (iii) $\text{E}\left({\left|\xi_{i}\right|}^{2+\epsilon }\right)<\infty$ *for some* ϵ>0. *We further assume that the signal* θ *is s-sparse*, *i.e*., $\|\theta {\|}_{0}\leq s$. *Denoting by* $M_{\xi_{1}^{2}}$ *the median of* $\xi_{1}^{2}$, *we consider the following estimator of* $\sigma^{2}$:

(A.47)
$${\bar{\sigma }}_{\text{MED}}^{2}=\text{median}\left(Y_{1}^{2},\;\ldots \;,Y_{d}^{2}\right)/M_{\xi_{1}^{2}}.$$

*Then*, *there exist constants* γ∈(0,1/8), C>0 *depending only on the cumulative distribution function of* $\xi_{1}$ *such that for all integers* s *and* d *satisfying* 1≤s<γd,

(A.48)
$$\underset{\sigma >0}{\text{sup}}\underset{\|\theta {\|}_{0}\leq s}{\text{sup}}\frac{1}{\sigma^{2}}\text{E}\left\{\left|{\bar{\sigma }}_{\text{MED}}^{2}-\sigma^{2}\right|\right\}\leq C\text{ max}\left(\frac{1}{d^{1/2}},\frac{s}{d}\right).$$

Building on Lemma 18, in Corollary 19, we analyze the properties of an estimator closely related to ${\hat{\sigma }}_{\text{MED}}^{2}$ in (22). In particular, this estimator ${\tilde{\sigma }}_{\text{MED}}^{2}$ does not subtract the median of each column in the input data. While ${\hat{\sigma }}_{\text{MED}}^{2}$ and ${\tilde{\sigma }}_{\text{MED}}^{2}$ are very similar provided that μ is sparse, we expect ${\hat{\sigma }}_{\text{MED}}^{2}$ to perform better empirically in scenarios where μ is sparse *up to a constant shift*, i.e., there exists a matrix C such that (i) each column of C takes on the same value; and (ii) μ+C is sparse.

**Corollary 19** *Under model* (1), *consider*

(A.49)
$${\tilde{\sigma }}_{\text{MED}}^{2}\left(X\right)=\left\{\underset{1\leq i\leq n,1\leq j\leq q}{\text{median}}\left(X_{ij}^{2}\right)\right\}/M_{\chi_{1}^{2}},$$

*where* $M_{\chi_{1}^{2}}$ *is the median of the* $\chi_{1}^{2}$ *distribution*. *Then, there exist constants* $\gamma_{0}\in \left(0,1/8\right)$, $c_{0}>0$ *such that for all integers* s *and* q *satisfying* $1\leq s<\gamma_{0}q$,

(A.50)
$$\underset{\sigma >0}{\text{sup}}\underset{\underset{1\leq i\leq n}{\text{max}}{\left\|\mu_{i}\right\|}_{0}\leq s}{\text{sup}}\frac{1}{\sigma^{2}}\text{E}\left\{\left|{\tilde{\sigma }}_{MED}^{2}-\sigma^{2}\right|\right\}\leq c_{0}\text{ max}\left\{\frac{1}{{\left(nq\right)}^{1/2}},\frac{s}{q}\right\}.$$

**Proof** First note that (1) can be re-written into the form of (A.46):

(A.51)
$$X_{ij}=\mu_{ij}+\sigma \xi_{ij},\;i=1,\;\ldots \;,n,j=1,\;\ldots \;,q,$$

where $\xi_{ij}$ is independently and identically distributed as 𝒩(0,1). Therefore, the estimator ${\tilde{\sigma }}_{\text{MED}}^{2}\left(X\right)$ in (A.49) is the estimator (A.47) applied to the model (A.51). Moreover, $\underset{1\leq i\leq n}{\text{max}}{\left\|\mu_{i}\right\|}_{0}\leq s$ implies that $\sum_{i=1}^{n}\sum_{j=1}^{q}1\left\{\mu_{ij}\neq 0\right\}\leq ns$. Applying Lemma 18, we have that

$$\underset{\sigma >0}{\text{sup}}\underset{\underset{1\leq i\leq n}{\text{max}}{\left\|\mu_{i}\right\|}_{0}\leq s}{\text{sup}}\frac{1}{\sigma^{2}}\text{E}\left\{\left|{\tilde{\sigma }}_{\text{MED}}^{2}(X)-\sigma^{2}\right|\right\}\leq c_{0}\text{max}\left\{\frac{1}{{\left(nq\right)}^{1/2}},\frac{ns}{nq}\right\}=c_{0}\text{ max}\left\{\frac{1}{{\left(nq\right)}^{1/2}},\frac{s}{q}\right\},$$

where $c_{0}$ is some universal constant. ■

In words, Corollary 19 states that under model (1), the rate of convergence of ${\tilde{\sigma }}_{\text{MED}}^{2}$ in mean (and therefore, in probability) is $\text{max}\left\{1/{\left(nq\right)}^{1/2},s/q\right\}$. In particular, ${\tilde{\sigma }}_{\text{MED}}^{2}$ is a consistent estimator of $\sigma^{2}$ provided that s/q→0 as q→∞.

Next, we investigate the property of the sample variance estimator ${\hat{\sigma }}_{\text{Sample}}^{2}$.

**Proposition 20** *Under model* (1), *for* ${\hat{\sigma }}_{\text{Sample}}^{2}\left(X\right)=\sum_{i=1}^{n}\sum_{j=1}^{q}{\left(X_{ij}-{\bar{X}}_{j}\right)}^{2}/\left(nq-q\right)$, *we have that*

(A.52)
$$\text{E}\left\{{\hat{\sigma }}_{\text{Sample}}^{2}\left(X\right)\right\}-\sigma^{2}=\frac{1}{2n\left(n-1\right)q}\overset{q}{\underset{j=1}{\sum }}\overset{n}{\underset{i=1}{\sum }}\overset{n}{\underset{i^{\prime }=1}{\sum }}{\left(\mu_{ij}-\mu_{i^{\prime }j}\right)}^{2}.$$

*Moreover*, *for any integers* s *and* q *such that* ns≤q, *we have that*, *for some constant* ${\tilde{c}}_{0}$,

(A.53)
$$\underset{\sigma >0}{\text{sup}}\underset{\underset{1\leq i\leq n}{\text{max}}{\left\|\mu_{i}\right\|}_{0}\leq s}{\text{sup}}\frac{1}{\sigma^{2}}\text{E}\left\{\left|{\hat{\sigma }}_{\text{Sample}}^{2}\left(X\right)-\sigma^{2}\right|\right\}\geq {\tilde{c}}_{0}\frac{s}{q}.$$

**Proof** We start with the proof of (A.52). Under (1), the following holds:

$$\text{E}\left\{{\hat{\sigma }}_{\text{Sample}}^{2}(X)\right\}=\text{E}\left\{\sum_{i=1}^{n}\sum_{j=1}^{q}{\left(X_{ij}-{\bar{X}}_{j}\right)}^{2}/(nq-q)\right\}\;=\frac{1}{(n-1)q}\text{E}\left[\sum_{i=1}^{n}\sum_{j=1}^{q}\left\{X_{ij}^{2}-{\left({\bar{X}}_{j}\right)}^{2}\right\}\right]\;=\frac{1}{(n-1)q}\sum_{i=1}^{n}\sum_{j=1}^{q}\left[\sigma^{2}+\mu_{ij}^{2}-\left\{\frac{\sigma^{2}}{n}+\frac{1}{n^{2}}{\left(\sum_{i^{\prime }=1}^{n}\mu_{i^{\prime }j}\right)}^{2}\right\}\right]\;=\sigma^{2}+\frac{1}{n^{2}(n-1)q}\sum_{i=1}^{n}\sum_{j=1}^{q}\left\{n^{2}\mu_{ij}^{2}-{\left(\sum_{i^{\prime }=1}^{n}\mu_{i^{\prime }j}\right)}^{2}\right\}\;=\sigma^{2}+\frac{1}{n(n-1)q}\sum_{j=1}^{q}\left\{\left(\sum_{i=1}^{n}n\mu_{ij}^{2}\right)-{\left(\sum_{i^{\prime }=1}^{n}\mu_{i^{\prime }j}\right)}^{2}\right\}\;=\sigma^{2}+\frac{1}{2n(n-1)q}\sum_{j=1}^{q}\sum_{i=1}^{n}\sum_{i^{\prime }=1}^{n}{\left(\mu_{ij}-\mu_{i^{\prime }j}\right)}^{2}.$$

Here, the last equality follows from Langrange’s identity, which states that $\left(\sum_{i=1}^{n}a_{i}^{2}\right)\left(\sum_{i=1}^{n}b_{i}^{2}\right)-{\left(\sum_{i=1}^{n}a_{i}b_{i}\right)}^{2}=1/2\sum_{i=1}^{n}\sum_{i^{\prime }=1}^{n}{\left(a_{i}b_{i^{\prime }}-a_{i^{\prime }}b_{i}\right)}^{2}$.

To prove the second statement, we consider a specific matrix $\tilde{\mu }\in {\mathbb{R}}^{n\times q}$ with exactly ns≤q non-zero entries. In addition, each column of $\tilde{\mu }$ has at most one non-zero entry and each row of $\tilde{\mu }$ has exactly s non-zero entries. This is possible because *ns* is assumed to be less than q. Finally, we assume that the square of the minimal non-zero entry of $\tilde{\mu }$, $\underset{i,j:{\tilde{\mu }}_{ij}\neq 0}{\text{min}}{\tilde{\mu }}_{ij}^{2}$, is lower bounded by some universal constant M. Then, we have that

$$\underset{\sigma >0}{\text{sup}}\underset{\underset{1\leq i\leq n}{\text{max}}{\left\|\mu_{i}\right\|}_{0}\leq s}{\text{sup}}\frac{1}{\sigma^{2}}\text{E}\left\{\left|{\hat{\sigma }}_{\text{Sample}}^{2}(X)-\sigma^{2}\right|\right\}\;\overset{a.}{\geq }\underset{\sigma >0}{\text{sup}}\frac{1}{\sigma^{2}}{\text{E}}_{X\sim ℳ𝒩\left(\tilde{\mu },{\text{I}}_{n},\sigma^{2}{\text{I}}_{q}\right)}\left\{\left|{\hat{\sigma }}_{\text{Sample}}^{2}(X)-\sigma^{2}\right|\right\}\;\overset{b.}{\geq }\underset{\sigma >0}{\text{sup}}\frac{1}{\sigma^{2}}{\text{E}}_{X\sim ℳ𝒩\left(\tilde{\mu },{\text{I}}_{n},\sigma^{2}{\text{I}}_{q}\right)}\left\{{\hat{\sigma }}_{\text{Sample}}^{2}(X)-\sigma^{2}\right\}\;\overset{c.}{\geq }\underset{\sigma >0}{\text{sup}}\frac{1}{\sigma^{2}}\frac{1}{2n(n-1)q}\sum_{j=1}^{q}\sum_{i=1}^{n}\sum_{i^{\prime }=1}^{n}{\left({\tilde{\mu }}_{ij}-{\tilde{\mu }}_{i^{\prime }j}\right)}^{2}\geq \underset{\sigma >0}{\text{sup}}\frac{1}{\sigma^{2}}\frac{1}{2n(n-1)q}\sum_{j=1}^{q}\sum_{i=1}^{n}\sum_{i^{\prime }=1}^{n}1\left\{{\tilde{\mu }}_{ij}\neq 0\right\}1\left\{{\tilde{\mu }}_{i^{\prime }j}=0\right\}{\left({\tilde{\mu }}_{ij}-{\tilde{\mu }}_{i^{\prime }j}\right)}^{2}\;\overset{d.}{\geq }\underset{\sigma >0}{\text{sup}}\frac{1}{\sigma^{2}}\frac{M(n-1)ns}{2n(n-1)q}\geq {\tilde{c}}_{0}\frac{s}{q}.$$

Here, a. follows from picking any $\tilde{\mu }$ satisfying the conditions outlined above, since by construction, $\underset{1\leq i\leq n}{\text{max}}{\left\|{\tilde{\mu }}_{i}\right\|}_{0}=s$. Steps b. and c. follow from the inequality E(|X|)≥E(X) and the expression for $\text{E}\left\{{\hat{\sigma }}_{\text{Sample}}^{2}\left(X\right)\right\}$ in (A.52), respectively. Finally, to prove d., we note that for each of the ns columns with exactly one non-zero element, there are n−1 pairs of $\left(i,i^{\prime }\right)$, i=1, … ,n; $i^{\prime }=1,\;\ldots \;,n$ such that the product $1\left\{{\tilde{\mu }}_{ij}\neq 0\right\}1\left\{{\tilde{\mu }}_{i^{\prime }j}=0\right\}$ is non-zero. Moreover, each of pair contributes at least M by the assumption that ${\text{min}}_{i,j:{\tilde{\mu }}_{ij}\neq 0}{\tilde{\mu }}_{ij}^{2}\geq M$. ■

Contrasting the results in Corollary 19 and Proposition 20, we note that, under (1), the convergence of ${\hat{\sigma }}_{\text{MED}}^{2}$ depends critically on the sparsity parameter s (or, equivalently, the $\ell_{0}$ norm of $\mu_{i}$), whereas the convergence of ${\hat{\sigma }}_{\text{Sample}}^{2}$ is determined by $\sum_{j=1}^{q}\sum_{i=1}^{n}\sum_{i^{\prime }=1}^{n}{\left(\mu_{ij}-\mu_{i^{\prime }j}\right)}^{2}$. Thus, in scenarios where the underlying means $\mu_{i},i=1,\ldots ,n$ are sparse (e.g., (23) in Section 5), we expect ${\tilde{\sigma }}_{\text{MED}}^{2}$ (and therefore its “centered” analog ${\hat{\sigma }}_{\text{MED}}^{2}$ in (22)) to be a less conservative estimator of $\sigma^{2}$. As a result, we expect the test based on ${\hat{p}}_{\text{selective}}\left({\hat{\sigma }}_{\text{MED}}\right)$ to be more powerful than that based on ${\hat{p}}_{\text{selective}}\left({\hat{\sigma }}_{\text{Sample}}\right)$, as shown in Figure 4 of Section 5.

### A.8 Additional power comparisons

In Section 5.2, we compared the conditional power of the tests based on $p_{\text{selective}}$, ${\hat{p}}_{\text{selective}}\left({\hat{\sigma }}_{\text{MED}}\right)$, and ${\hat{p}}_{\text{selective}}\left({\hat{\sigma }}_{\text{Sample}}\right)$ under (23). Here, we conduct two additional analyses.

In the first analysis, we consider a different notion of power that does not condition on ${\hat{𝒞}}_{1}$ and ${\hat{𝒞}}_{2}$ being true clusters. In this case, comparing the power of the tests requires a bit of care, because the effect size ${\left\|\mu^{⊤}\nu \right\|}_{2}$ may differ across simulated datasets from the same data-generating distribution. As a result, we consider the power of the tests as a function of ${\left\|\mu^{⊤}\nu \right\|}_{2}$. We fit a regression spline using the gam function in the R package mgcv (Wood, 2017) to obtain a smooth estimate of power on the same simulated datasets from Section 5.2. The results are in Figure 6. The power of the tests that reject $H_{0}$ if $p_{\text{selective}}$, ${\hat{p}}_{\text{selective}}\left({\hat{\sigma }}_{\text{MED}}\right)$, or ${\hat{p}}_{\text{selective}}\left({\hat{\sigma }}_{\text{Sample}}\right)$ is less than α=0.05 increases as $\mu^{⊤}\nu_{2}$ increases. For a given value of $\mu^{⊤}\nu_{2}$ and σ, the test based on $p_{\text{selective}}$ has the highest power, followed by that based on ${\hat{p}}_{\text{selective}}\left({\hat{\sigma }}_{\text{MED}}\right)$; the test based on ${\hat{p}}_{\text{selective}}\left({\hat{\sigma }}_{\text{Sample}}\right)$ has the lowest power.

In the second analysis, we consider the conditional power (defined in (24)) of the tests based on $p_{\text{selective}}$, ${\hat{p}}_{\text{selective}}\left({\hat{\sigma }}_{\text{MED}}\right)$, and ${\hat{p}}_{\text{selective}}\left({\hat{\sigma }}_{\text{Sample}}\right)$ under a different data generating model than (23). We generate data from (1) with n=150 and

(A.54)
$$\mu_{1}=\ldots =\mu_{\frac{n}{3}}=\left[\begin{array}{c} \theta_{1}\\ 0_{0.9q} \end{array}\right],\mu_{\frac{n}{3}+1}=\ldots =\mu_{\frac{2n}{3}}=\left[\begin{array}{c} \theta_{2}\\ 0_{0.9q} \end{array}\right],\mu_{\frac{2n}{3}+1}=\ldots =\mu_{n}=\left[\begin{array}{c} \theta_{3}\\ 0_{0.9q} \end{array}\right],$$

where, q is taken to be a multiple of 10, and for δ>0, $\theta \in {\mathbb{R}}^{3\times 0.1q}$ has orthogonal rows, with ${\left\|\theta_{i}\right\|}_{2}^{2}=\delta /2$ for i=1,2,3. As in Section 5.2, we can think of ${𝒞}_{1}=\left\{1,\;\ldots \;,n/3\right\}$, ${𝒞}_{2}=\left\{\left(n/3\right)+1,\;\ldots \;,\left(2n/3\right)\right\}$, ${𝒞}_{3}=\left\{\left(2n/3\right)+1,\;\ldots \;,n\right\}$ as “true clusters”. Under (A.54), the pairwise distance between each pair of true clusters is δ.

We generate M=100,000 datasets from (A.54) with q=50, σ=0.25,0.5,1, and δ=2,3, … ,10. For each simulated dataset, we apply k-means clustering with K=3 and reject $H_{0}:\mu^{⊤}\nu =0_{q}$ if $p_{\text{selective}}$, ${\hat{p}}_{\text{selective}}\left({\hat{\sigma }}_{\text{MED}}\right)$, or ${\hat{p}}_{\text{selective}}\left({\hat{\sigma }}_{\text{Sample}}\right)$ is less than α=0.05. Figure 7(a) displays the detection probability (25) of k-means clustering as a function of δ in (A.54). Under model (1), the detection probability increases as a function of δ in (A.54) across all values of σ. For a given value of δ, a larger value of σ leads to lower detection probability. Figure 7(b) displays the conditional power (24) for the tests based on $p_{\text{selective}}$, ${\hat{p}}_{\text{selective}}\left({\hat{\sigma }}_{\text{MED}}\right)$, and . For some combinations of δ and σ, the conditional power is not displayed, because the true clusters are never recovered in simulation. For all tests and values of σ under consideration, conditional power is an increasing function of δ. For a given test and a value of δ, smaller σ leads to higher conditional power. Moreover, for the same values of δ and σ, the test based on $p_{\text{selective}}$ has the highest conditional power, followed closely by the test based of ${\hat{p}}_{\text{selective}}\left({\hat{\sigma }}_{\text{MED}}\right)$. Using ${\hat{p}}_{\text{selective}}\left({\hat{\sigma }}_{\text{Sample}}\right)$ leads to a less powerful test, especially for larger values of δ. As a comparison, we included the detection probability and conditional power under model (23) with q=50 in panels (c) and (d) of Figure 7. The tests under consideration behave qualitatively similarly as a function of δ and σ. Under (23), we observe an even larger gap between the power of the test based on ${\hat{p}}_{\text{selective}}\left({\hat{\sigma }}_{\text{Sample}}\right)$ and the power of the test based on ${\hat{p}}_{\text{selective}}\left({\hat{\sigma }}_{\text{MED}}\right)$.

### A.9 Additional results for real data applications

In this section, we visualize the estimated clusters for the single cell RNA-sequencing data in Section 6.2.

## References

[R1] Aizarani Nadim, Saviano Antonio, Sagar, Mailly Laurent, Durand Sarah, Herman Josip S, Pessaux Patrick, Baumert Thomas F, and Grün Dominic. A human liver cell atlas reveals heterogeneity and epithelial progenitors. Nature, 572(7768):199–204, August 2019. 31292543 10.1038/s41586-019-1373-2 PMC6687507

[R2] Aloise Daniel, Deshpande Amit, Hansen Pierre, and Popat Preyas. NP-hardness of Euclidean sum-of-squares clustering. Machine Learning, 75(2):245–248, May 2009.

[R3] Arthur David and Vassilvitskii Sergei. k-means++: the advantages of careful seeding. In Proceedings of the Eighteenth Annual ACM-SIAM Symposium On Discrete Algorithms, SODA ‘07, pages 1027–1035, USA, January 2007. Society for Industrial and Applied Mathematics.

[R4] Avella-Medina Marco, Battey Heather S, Fan Jianqing, and Li Quefeng. Robust estimation of high-dimensional covariance and precision matrices. Biometrika, 105(2):271–284, March 2018. 30337763 10.1093/biomet/asy011 PMC6188670

[R5] Aw Alan J, Spence Jeffrey P, and Song Yun S. A flexible and robust non-parametric test of exchangeability. arXiv:2109.15261, September 2021.

[R6] Balakrishnan Sivaraman, Wainwright Martin J, and Yu Bin. Statistical guarantees for the EM algorithm: From population to sample-based analysis. The Annals of Statistics, 45 (1):77–120, February 2017.

[R7] Bell Anthony J and Sejnowski Terrence J. The “independent components” of natural scenes are edge filters. Vision Research, 37(23):3327–3338, December 1997. 9425547 10.1016/s0042-6989(97)00121-1 PMC2882863

[R8] Belomestny Denis, Trabs Mathias, and Tsybakov Alexandre B. Sparse covariance matrix estimation in high-dimensional deconvolution. Bernoulli, 25(3):1901–1938, 2019. doi: 10.3150/18-BEJ1040A. URL 10.3150/18-BEJ1040A.

[R9] Benjamini Yuval, Taylor Jonathan, and Irizarry Rafael A. Selection-corrected statistical inference for region detection with high-throughput assays. Journal of the American Statistical Association, 114(527):1351–1365, July 2019. 36312875 10.1080/01621459.2018.1498347 PMC9615469

[R10] Bickel Peter J and Levina Elizaveta. Covariance regularization by thresholding. The Annals of Statistics, 36(6):2577–2604, December 2008.

[R11] Bilodeau Martin and Brenner David. Theory of Multivariate Statistics. Springer, New York, NY, 1999.

[R12] Bourgon Richard. intervals: Tools for working with points and intervals. https://cran.rstudio.com/web/packages/intervals/index.html, 2020. Accessed: 2022-2-11.

[R13] Button Katherine S. Double-dipping revisited. Nature Neuroscience, 22(5):688–690, May 2019. 31011228 10.1038/s41593-019-0398-z

[R14] Cai Diana, Campbell Trevor, and Broderick Tamara. Finite mixture models do not reliably learn the number of components. arXiv:2007.04470, July 2020.

[R15] Tony T Cai Jing Ma, and Zhang Linjun. CHIME: Clustering of high-dimensional gaussian mixtures with EM algorithm and its optimality. The Annals of Statistics, 47(3):12341267, June 2019.

[R16] Charkhi Ali and Claeskens Gerda. Asymptotic post-selection inference for the Akaike information criterion. Biometrika, 105(3):645–664, June 2018.

[R17] Chen Hanfeng, Chen Jiahua, and Kalbfleisch John D. Testing for a finite mixture model with two components. J. R. Stat. Soc. Series B Stat. Methodol, 66(1):95–115, February 2004.

[R18] Chen Jiahua and Li Pengfei. Hypothesis test for normal mixture models: The EM approach. The Annals of Statistics, 37(5A):2523–2542, October 2009.

[R19] Chen Mengjie, Gao Chao, and Ren Zhao. Robust covariance and scatter matrix estimation under Huber’s contamination model. Annals of Statistics, 46(5), October 2018.

[R20] Chen Shuxiao and Bien Jacob. Valid inference corrected for outlier removal. J. Comput. Graph. Stat, 29(2):323–334, April 2020.

[R21] Chen Yiqun, Jewell Sean, and Witten Daniela. More powerful selective inference for the graph fused lasso. Journal of Computational and Graphical Statistics, pages 1–11, 2022. 10.1080/10618600.2022.2097246 PMC10798806 38250478

[R22] Chen Yiqun T, Jewell Sean W, and Witten Daniela M. Quantifying uncertainty in spikes estimated from calcium imaging data. Biostatistics, October 2021. 10.1093/biostatistics/kxab034 PMC10449000 34654923

[R23] Chung Neo Christopher. Statistical significance of cluster membership for unsupervised evaluation of cell identities. Bioinformatics, 36(10):3107–3114, May 2020. 32142108 10.1093/bioinformatics/btaa087 PMC7214036

[R24] Chung Neo Christopher and Storey John D. Statistical significance of variables driving systematic variation in high-dimensional data. Bioinformatics, 31(4):545–554, February 2015. 25336500 10.1093/bioinformatics/btu674 PMC4325543

[R25] Coates Adam and Ng Andrew Y. Learning feature representations with K-Means. In Montavon Grégoire, Orr Geneviève B, and Müller Klaus-Robert, editors, Neural Networks: Tricks of the Trade: Second Edition, pages 561–580. Springer Berlin Heidelberg, Berlin, Heidelberg, 2012.

[R26] Comminges L, Collier O, Ndaoud M, and Tsybakov AB. Adaptive robust estimation in sparse vector model. Annals of Statistics, 49(3), June 2021.

[R27] Dobriban Edgar. Permutation methods for factor analysis and PCA. Annals of Statistics, 48(5), October 2020.

[R28] Doughty Tyler and Kerkhoven Eduard. Extracting novel hypotheses and findings from RNA-seq data. FEMS Yeast Res, 20(2), March 2020. 10.1093/femsyr/foaa007 PMC7029681 32009158

[R29] Duò Angelo, Robinson Mark D, and Soneson Charlotte. A systematic performance evaluation of clustering methods for single-cell RNA-seq data. F1000Research, 7:1141, July 2018. 30271584 10.12688/f1000research.15666.1 PMC6134335

[R30] Duy VNL, Toda H, Sugiyama R, and Takeuchi I. Computing valid p-value for optimal changepoint by selective inference using dynamic programming. arXiv:2002.09132, 2020.

[R31] Eling Nils, Richard Arianne C, Richardson Sylvia, Marioni John C, and Vallejos Catalina A. Correcting the mean-variance dependency for differential variability testing using single-cell RNA sequencing data. Cell Systems, 7(3):284–294.e12, September 2018. 30172840 10.1016/j.cels.2018.06.011 PMC6167088

[R32] Fithian William, Sun Dennis, and Taylor Jonathan. Optimal inference after model selection. arXiv:1410.2597, October 2014.

[R33] Friederich Pascal, Krenn Mario, Tamblyn Isaac, and Aspuru-Guzik Alan. Scientific intuition inspired by machine learning generated hypotheses. arXiv:2010.14236, October 2020.

[R34] Gallaugher Michael P B and McNicholas Paul D. Finite mixtures of skewed matrix variate distributions. Pattern Recognition, 80:83–93, August 2018.

[R35] Gao Lucy L, Bien Jacob, and Witten Daniela. Selective inference for hierarchical clustering. Journal of the American Statistical Association, pages 1–27, December 2022. 38660582 10.1080/01621459.2022.2116331 PMC11036349

[R36] Grün Dominic, Lyubimova Anna, Kester Lennart, Wiebrands Kay, Basak Onur, Sasaki Nobuo, Clevers Hans, and van Oudenaarden Alexander. Single-cell messenger RNA sequencing reveals rare intestinal cell types. Nature, 525(7568):251–255, September 2015. 26287467 10.1038/nature14966

[R37] Guha Aritra, Ho Nhat, and Nguyen Xuanlong. On posterior contraction of parameters and interpretability in bayesian mixture modeling. arXiv:1901.05078, January 2019.

[R38] Han Fang and Liu Han. Scale-invariant sparse PCA on high dimensional meta-elliptical data. Journal of the American Statistical Association, 109(505):275–287, January 2014. 24932056 10.1080/01621459.2013.844699 PMC4051512

[R39] Hartigan JA and Wong MA. Algorithm AS 136: A k-means clustering algorithm. Journal of the Royal Statistical Society. Series C, Applied statistics, 28(1):100–108, 1979.

[R40] Hastie Trevor., Hastie Trevor., Tibshirani Robert., Friedman, and J H. The Elements of Statistical Learning : data mining, inference, and prediction. Springer, New York, 2001.

[R41] Horn Roger A and Johnson Charles R. Matrix Analysis. Cambridge University Press, 2nd edition edition, October 2012.

[R42] Huber Peter J. Robust Statistics. John Wiley & Sons, 1981.

[R43] Hung Kenneth and Fithian William. Statistical methods for replicability assessment. The Annals of Applied Statistics, 14(3):1063–1087, September 2020.

[R44] Hyun Sangwon, G’Sell Max, and Tibshirani Ryan J. Exact post-selection inference for the generalized lasso path. Electron. J. Stat, 12(1):1053–1097, 2018.

[R45] Hyun Sangwon, Lin Kevin Z, G’Sell Max, and Tibshirani Ryan J. Post-selection inference for changepoint detection algorithms with application to copy number variation data. Biometrics, January 2021. 10.1111/biom.13422 33434289

[R46] Jewell Sean, Fearnhead Paul, and Witten Daniela. Testing for a change in mean after changepoint detection. To appear in J. R. Stat. Soc. Series B Stat. Methodol, 2022. 10.1111/rssb.12501 PMC9678373 36419504

[R47] Jin Jiashun and Wang Wanjie. Influential features PCA for high dimensional clustering. The Annals of Statistics, 44(6):2323–2359, December 2016.

[R48] Kerr MK and Churchill GA. Bootstrapping cluster analysis: assessing the reliability of conclusions from microarray experiments. Proceedings of the National Academy of Sciences of the United States of America, 98(16):8961–8965, July 2001. 11470909 10.1073/pnas.161273698 PMC55356

[R49] Kimes Patrick K, Liu Yufeng, Hayes David Neil, and Marron James Stephen. Statistical significance for hierarchical clustering. Biometrics, 73(3):811–821, September 2017. 28099990 10.1111/biom.12647 PMC5708128

[R50] Kriegeskorte Nikolaus, Simmons W Kyle, Bellgowan Patrick S F, and Baker Chris I. Circular analysis in systems neuroscience: the dangers of double dipping. Nature Neuroscience, 12(5):535–540, May 2009. 19396166 10.1038/nn.2303 PMC2841687

[R51] Lähnemann David, Köster Johannes, Szczurek Ewa, McCarthy Davis J, Hicks Stephanie C, Robinson Mark D, Vallejos Catalina A, Campbell Kieran R, Beerenwinkel Niko, Mahfouz Ahmed, Pinello Luca, Skums Pavel, Stamatakis Alexandros, Stephan-Otto Attolini Camille, Aparicio Samuel, Baaijens Jasmijn, Balvert Marleen, de Barbanson Buys, Cappuccio Antonio, Corleone Giacomo, Dutilh Bas E, Florescu Maria, Guryev Victor, Holmer Rens, Jahn Katharina, Lobo Thamar Jessurun, Keizer Emma M, Khatri Indu, Kielbasa Szymon M, Korbel Jan O, Kozlov Alexey M, Kuo Tzu-Hao, Lelieveldt Boudewijn P F, Mandoiu Ion I, Marioni John C, Marschall Tobias, Mölder Felix, Niknejad Amir, Raczkowski Lukasz, Reinders Marcel, de Ridder Jeroen, Saliba Antoine-Emmanuel, Somarakis Antonios, Stegle Oliver, Theis Fabian J, Yang Huan, Zelikovsky Alex, McHardy Alice C, Raphael Benjamin J, Shah Sohrab P, and Schönhuth Alexander. Eleven grand challenges in single-cell data science. Genome Biology, 21(1):31, February 2020. 32033589 10.1186/s13059-020-1926-6 PMC7007675

[R52] Le Duy Vo Nguyen and Takeuchi Ichiro. More powerful conditional selective inference for generalized lasso by parametric programming. arXiv:2105.04920, May 2021.

[R53] Lecun Y, Bottou L, Bengio Y, and Haffner P. Gradient-based learning applied to document recognition. Proceedings of the IEEE, 86(11):2278–2324, November 1998.

[R54] Lee Jason D, Sun Dennis L, Sun Yuekai, and Taylor Jonathan E. Exact post-selection inference, with application to the lasso. The Annals of Statistics, 44(3):907–927, June 2016.

[R55] Leiner James, Duan Boyan, Wasserman Larry, and Ramdas Aaditya. Data blurring: sample splitting a single sample. arXiv:2112.11079, December 2021.

[R56] Li Pengfei and Chen Jiahua. Testing the order of a finite mixture. Journal of the American Statistical Association, 105(491):1084–1092, September 2010.

[R57] Lin Kevin Z, Lei Jing, and Roeder Kathryn. Exponential-family embedding with application to cell developmental trajectories for single-cell RNA-Seq data. Journal of the American Statistical Association, 116(534):457–470, April 2021. 34354320 10.1080/01621459.2021.1886106 PMC8336573

[R58] Liu Keli, Markovic Jelena, and Tibshirani Robert. More powerful post-selection inference, with application to the lasso. arXiv:1801.09037, January 2018.

[R59] Lloyd S. Least squares quantization in PCM. IEEE Trans. Inf. Theory, 28(2):129–137, September 1982.

[R60] Löffler Matthias, Zhang Anderson Y, and Zhou Harrison H. Optimality of spectral clustering in the Gaussian mixture model. The Annals of Statistics, 49(5):2506–2530, October 2021.

[R61] Loftus Joshua R and Taylor Jonathan E. Selective inference in regression models with groups of variables. arXiv preprint arXiv:1511.01478, 2015.

[R62] Lu Yu and Zhou Harrison H. Statistical and computational guarantees of Lloyd’s algorithm and its variants. arXiv:1612.02099, December 2016.

[R63] MacQueen J, and author. Some methods for classification and analysis of multivariate observations. In Proceedings of the Fifth Berkeley Symposium on Mathematical Statistics and Probability, Volume 1: Statistics, pages 281–297. University of California Press, January 1967.

[R64] Markovic Jelena, Xia Lucy, and Taylor Jonathan. Unifying approach to selective inference with applications to cross-validation. arXiv:1703.06559, 2017.

[R65] McInnes Leland, Healy John, and Melville James. UMAP: Uniform manifold approximation and projection for dimension reduction. arXiv:1802.03426, February 2018.

[R66] McLachlan Geoffrey J, Lee Sharon X, and Rathnayake Suren I. Finite mixture models. Annual Review of Statistics and Its Application, March 2019.

[R67] Nobile Agostino. On the posterior distribution of the number of components in a finite mixture. The Annals of Statistics, 32(5):2044–2073, October 2004.

[R68] Pollice Robert, Dos Passos Gomes Gabriel, Aldeghi Matteo, Hickman Riley J, Krenn Mario, Lavigne Cyrille, Lindner-D’Addario Michael, Nigam Akshatkumar, Ser Cher Tian, Yao Zhenpeng, and Aspuru-Guzik Alán. Data-Driven strategies for accelerated materials design. Accounts of Chemical Research, 54(4):849–860, February 2021. 33528245 10.1021/acs.accounts.0c00785 PMC7893702

[R69] Rasines Daniel G and Young G Alastair. Splitting strategies for post-selection inference. arXiv:2102.02159, February 2021.

[R70] Risso Davide, Perraudeau Fanny, Gribkova Svetlana, Dudoit Sandrine, and Vert Jean-Philippe. A general and flexible method for signal extraction from single-cell RNA-seq data. Nature Communications, 9(1):1–17, January 2018. 10.1038/s41467-017-02554-5 PMC5773593 29348443

[R71] Rousseeuw Peter J. Robust Regression and Outlier Detection. Wiley, New York, 1987.

[R72] Rügamer David, Baumann Philipp F M, and Greven Sonja. Selective inference for additive and linear mixed models. Computational Statistics & Data Analysis, 167:107350, March 2022.

[R73] Schultheiss Christoph, Renaux Claude, and Bühlmann Peter. Multicarving for high-dimensional post-selection inference. Electron. J. Stat, 15(1):1695–1742, January 2021.

[R74] Stuart Tim, Butler Andrew, Hoffman Paul, Hafemeister Christoph, Papalexi Efthymia, Mauck William M 3rd, Hao Yuhan, Stoeckius Marlon, Smibert Peter, and Satija Rahul. Comprehensive integration of Single-Cell data. Cell, 177(7):1888–1902.e21, June 2019. 31178118 10.1016/j.cell.2019.05.031 PMC6687398

[R75] Suzuki Ryota and Shimodaira Hidetoshi. Pvclust: an R package for assessing the uncertainty in hierarchical clustering. Bioinformatics, 22(12):1540–1542, June 2006. 16595560 10.1093/bioinformatics/btl117

[R76] Taylor Jonathan and Tibshirani Robert. Post-selection inference for *ℓ*_1_-penalized likelihood models. The Canadian Journal of Statistics, 46(1):41–61, March 2018. 30127543 10.1002/cjs.11313 PMC6097808

[R77] Tibshirani Ryan J, Taylor Jonathan, Lockhart Richard, and Tibshirani Robert. Exact post-selection inference for sequential regression procedures. Journal of the American Statistical Association, 111(514):600–620, April 2016.

[R78] Tibshirani Ryan J, Rinaldo Alessandro, Tibshirani Rob, and Wasserman Larry. Uniform asymptotic inference and the bootstrap after model selection. The Annals of Statistics, 46(3):1255–1287, June 2018.

[R79] Wang Zhaoran, Gu Quanquan, Ning Yang, and Liu Han. High dimensional EM algorithm: Statistical optimization and asymptotic normality. Advances in Neural Information Processing Systems, 28:2512–2520, 2015. 28615917 PMC5467221

[R80] Watanabe Chihiro and Suzuki Taiji. Selective inference for latent block models. Electron. J. Stat, 15(1), January 2021.

[R81] Witten Daniela M. Classification and clustering of sequencing data using a Poisson model. The Annals of Applied Statistics, 5(4):2493–2518, December 2011.

[R82] Wood SN. Generalized Additive Models: An Introduction with R. Chapman and Hall/CRC, 2017.

[R83] Xu Rui and Wunsch Don. Clustering. John Wiley & Sons, November 2008.

[R84] Yang Fan, Barber Rina Foygel, Jain Prateek, and Lafferty John. Selective inference for group-sparse linear models. In Proceedings of the 30th International Conference on Neural Information Processing Systems, NIPS’16, pages 2477–2485, Red Hook, NY, USA, December 2016. Curran Associates Inc.

[R85] Yi Xinyang and Caramanis Constantine. Regularized EM algorithms: A unified framework and statistical guarantees. Advances in Neural Information Processing Systems, 28, 2015. PMC5388070 28408793

[R86] Zha Hongyuan, He Xiaofeng, Ding Chris, Gu Ming, and Simon Horst. Spectral relaxation for k-means clustering. In Dietterich T, Becker S, and Ghahramani Z, editors, Advances in Neural Information Processing Systems, volume 14. MIT Press, 2002.

[R87] Zhang Cun-Hui and Zhang Stephanie S. Confidence intervals for low dimensional parameters in high dimensional linear models. J. R. Stat. Soc. Series B Stat. Methodol, 76(1):217–242, 2014.

[R88] Zhang Jesse M, Kamath Govinda M, and Tse David N. Valid post-clustering differential analysis for Single-Cell RNA-Seq. Cell Systems, 9(4):383–392.e6, October 2019. 31521605 10.1016/j.cels.2019.07.012 PMC7202736

[R89] Zheng Grace X Y, Terry Jessica M, Belgrader Phillip, Ryvkin Paul, Bent Zachary W, Wilson Ryan, Ziraldo Solongo B, Wheeler Tobias D, McDermott Geoff P, Zhu Junjie, Gregory Mark T, Shuga Joe, Montesclaros Luz, Underwood Jason G, Masquelier Donald A, Nishimura Stefanie Y, Schnall-Levin Michael, Wyatt Paul W, Hindson Christopher M, Bharadwaj Rajiv, Wong Alexander, Ness Kevin D, Beppu Lan W, Deeg H Joachim, McFarland Christopher, Loeb Keith R, Valente William J, Ericson Nolan G, Stevens Emily A, Radich Jerald P, Mikkelsen Tarjei S, Hindson Benjamin J, and Bielas Jason H. Massively parallel digital transcriptional profiling of single cells. Nature Communications, 8:14049, January 2017. 10.1038/ncomms14049 PMC5241818 28091601

